# Co-targeting SOS1 enhances the antitumor effects of KRAS^G12C^ inhibitors by addressing intrinsic and acquired resistance

**DOI:** 10.1038/s43018-024-00800-6

**Published:** 2024-08-05

**Authors:** Venu Thatikonda, Hengyu Lyu, Sabine Jurado, Kaja Kostyrko, Christopher A. Bristow, Christoph Albrecht, Donat Alpar, Heribert Arnhof, Oliver Bergner, Karin Bosch, Ningping Feng, Sisi Gao, Daniel Gerlach, Michael Gmachl, Melanie Hinkel, Simone Lieb, Astrid Jeschko, Annette A. Machado, Thomas Madensky, Ethan D. Marszalek, Mikhila Mahendra, Gabriella Melo-Zainzinger, Jessica M. Molkentine, Philipp A. Jaeger, David H. Peng, Robyn L. Schenk, Alexey Sorokin, Sandra Strauss, Francesca Trapani, Scott Kopetz, Christopher P. Vellano, Mark Petronczki, Norbert Kraut, Timothy P. Heffernan, Joseph R. Marszalek, Mark Pearson, Irene C. Waizenegger, Marco H. Hofmann

**Affiliations:** 1 Boehringer Ingelheim RCV, Vienna, Austria; 2 Translational Research to Advance Therapeutics and Innovation in Oncology (TRACTION) Platform, Therapeutics Discovery Division, The University of Texas MD Anderson Cancer Center, Houston, TX USA; 3 Department of Gastrointestinal Medical Oncology, The University of Texas MD Anderson Cancer Center, Houston, TX USA; 4 Present Address: Exscientia, Vienna, Austria

**Keywords:** Cancer therapy, Cancer therapeutic resistance, Cancer

## Abstract

Combination approaches are needed to strengthen and extend the clinical response to KRAS^G12C^ inhibitors (KRAS^G12C^i). Here, we assessed the antitumor responses of KRAS^G12C^ mutant lung and colorectal cancer models to combination treatment with a SOS1 inhibitor (SOS1i), BI-3406, plus the KRAS^G12C^ inhibitor, adagrasib. We found that responses to BI-3406 plus adagrasib were stronger than to adagrasib alone, comparable to adagrasib with SHP2 (SHP2i) or EGFR inhibitors and correlated with stronger suppression of RAS-MAPK signaling. BI-3406 plus adagrasib treatment also delayed the emergence of acquired resistance and elicited antitumor responses from adagrasib-resistant models. Resistance to KRAS^G12C^i seemed to be driven by upregulation of MRAS activity, which both SOS1i and SHP2i were found to potently inhibit. Knockdown of *SHOC2*, a MRAS complex partner, partially restored response to KRAS^G12C^i treatment. These results suggest KRAS^G12C^ plus SOS1i to be a promising strategy for treating both KRAS^G12C^i naive and relapsed KRAS^G12C^-mutant tumors.

## Abstract (web summary)

Hofmann and colleagues describe the mechanism underlying the therapeutic benefit of combinatorial use of SOS1 and KRAS-G12C inhibitors in the context of KRAS-G12C mutant-driven lung and colorectal cancer.

## Main

*KRAS* alterations are frequently found in human cancers^1^, with G12C mutations occurring in 13% of non-small cell lung cancer (NSCLC) and 3% of colorectal cancer (CRC)^1^. Recent drug-discovery efforts^2^ have led to clinical trials of KRAS^G12C^-specific inhibitors (KRAS^G12C^i)^1,3^, with sotorasib (AMG 510)^4,5^ and adagrasib (MRTX849)^6^ both receiving accelerated approval in KRAS^G12C^-mutated NSCLC^7,8^. Despite this success, disease progression occurs in many patients^4,5,7^, with development of resistance to KRAS^G12C^i recapitulated in preclinical studies^9–11^. Effective combination approaches are therefore needed to improve the response to KRAS^G12C^i (refs. ^1,9^).

KRAS is a GTPase that cycles between inactive (GDP-bound) and active (GTP-bound) states. Guanine nucleotide exchange factors (GEFs) promote GTP-loading of KRAS, while an intrinsic GTPase activity, enhanced by GTPase-activating proteins, returns KRAS to the inactive state^1^. Activating mutations in *KRAS* commonly result in the persistence of the GTP-bound state, enhance downstream signaling and lead to uncontrolled cell growth^1^. Approved KRAS^G12C^i lock KRAS^G12C^ in the GDP-bound state^12^. Preclinical and clinical studies, however, have demonstrated that cancers lose sensitivity to KRAS^G12C^i and re-establish RAS-MAPK signaling through multiple mechanisms, including increased activation of mutant or wild-type (WT) RAS and/or gain of upstream/downstream bypass events^1,7,9,10,13,14^.

Combination approaches that co-target regulators upstream of KRAS, including receptor tyrosine kinases (RTKs) and SHP2, improve KRAS^G12C^i response in preclinical models^13,15–17^. Mechanistically, this approach may block pathway feedback activation and shift both mutant and WT RAS to the GDP-bound state, thus enhancing downregulation of RAS-MAPK pathway signaling by KRAS^G12C^i (refs. ^13,16^). Accordingly, KRAS^G12C^i plus SHP2 inhibitor (SHP2i; for example TNO155) or EGFR antagonist (EGFRi; for example cetuximab^18^) combination approaches are now being clinically tested^13,16,19,20^, with evidence of improved clinical response^20–22^.

In pursuit of optimizing the response to KRAS^G12C^i, we have developed two specific and potent SOS1 inhibitors (SOS1i), BI-3406 and the clinical candidate BI-1701963, which target SOS1-RAS binding^23^. SOS1 catalyzes RAS-GTP-loading as a GEF^24,25^ and serves as a key node of feedback regulation^23,24^. Accordingly, we hypothesize that co-targeting SOS1 plus KRAS^G12C^ will enrich the inactive GDP-bound KRAS^G12C^ and block RTK-mediated WT RAS activation, potentially resulting in a more durable clinical response^16^. Indeed, in KRAS^G12C^-mutant NSCLC and CRC models, co-administering SOS1i with KRAS^G12C^i enhanced the antitumor response comparably to adagrasib plus SHP2i or EGFRi, delayed the emergence of acquired resistance and reestablished antitumor responses in KRAS^G12C^i-resistant models. Finally, both SOS1i and SHP2i were found to potently inhibit upregulation of MRAS, an emerging resistance mechanism to KRAS^G12C^i.

## Results

### Combination therapy enhances antiproliferative responses

**Fig. 1:**
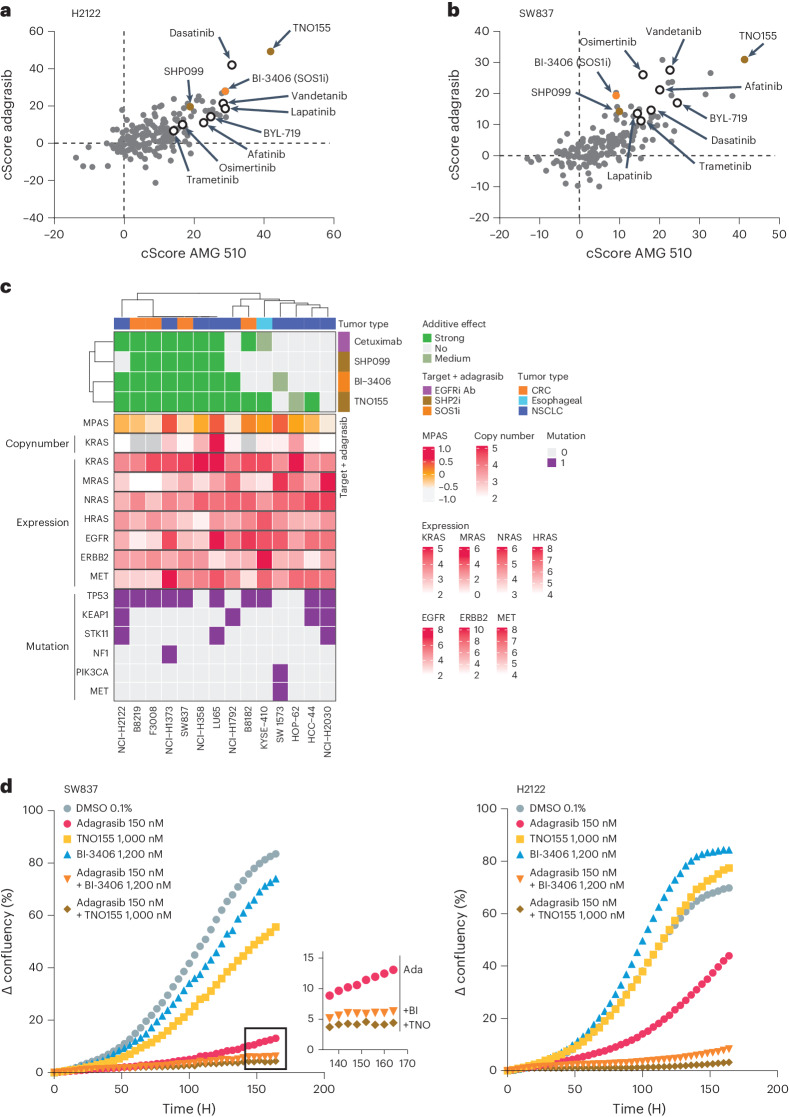
Efficacy of combination treatments in non-small cell lung cancer and colorectal cancer cell lines. **a**, Comparison of combination scores (cScore) indicating the response of KRAS^G12C^ mutant NCI-H2122 cells to the combination treatment of AMG 510 or adagrasib with 179 other small molecules for 72 h. Positive scores indicate additive effects with scores >~10 generally indicating a ‘clear additive’ effect. Empty or orange/brown colored circles highlight selected compounds for reference. *n* = 1 independent culture per cell line, 2–72 technical replicates per treatment (Supplementary Table 1). **b**, Comparison of cScores indicating the response of KRAS^G12C^ mutant SW837 cells to combination treatment of AMG 510 (sotorasib) or adagrasib for 144 h. Positive scores and empty/colored circles are explained in **a**. *n* = 1 independent culture per cell line, 2–72 technical replicates per treatment (Supplementary Table 1). **c**, Response of indicated cell lines to the combination treatment of adagrasib with a SOS1 inhibitor (BI-3406), a SHP2 inhibitor (TNO155 or SHP099) or an EGFR inhibitor (cetuximab). Cell lines are indicated as CRC (orange), esophageal cancer (light blue) or NSCLC (dark blue). MPAS, *KRAS* copy number status, *K/H/N/MRAS* expression and co-occurring mutations for each cell line are indicated. A strong additive benefit is defined with a cell growth inhibition (CGI) ≥ 50 and a Bliss CGI value ≥ 10 in ≥5 neighboring wells; medium additive benefit is defined with a CGI ≥ 30 and a Bliss CGI value ≥ 10 in ≥5 neighboring wells. *n* = 2 independent cultures per cell line. **d**, Growth kinetic of SW837 (left) or NCI-H2122 (right) cells treated with indicated single or combination treatments or vehicle control. The *y* axis indicates change in confluency relative to *t* = 0 h. Inset shows boxed area for clarity. *n* = 1 culture per curve, three technical replicates per culture. Source data

**Extended Data Fig. 1:**
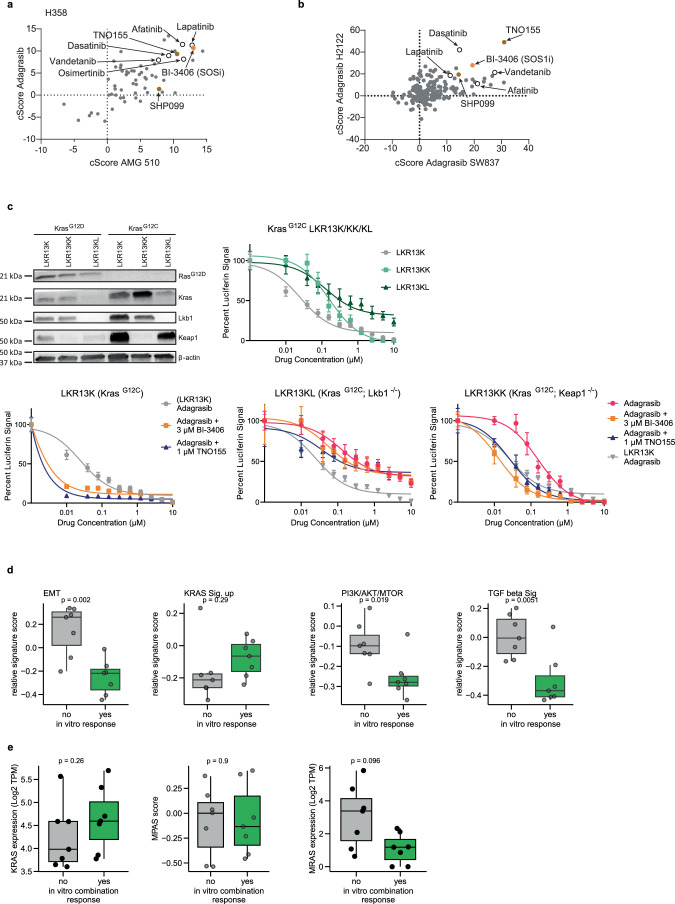
Molecular signatures of KRAS^G12C^-driven tumor cell lines. **a**, Comparison of combination scores (cScore) indicating the response of KRAS^G12C^ mutant NCI-H358 cells to combination treatment of AMG 510 (sotorasib) or adagrasib for 144 hours on a sub-panel of 61 representative compounds. Positive scores indicating additive effects with scores >~10 generally indicating a “clear additive effect.” Empty or orange/brown colored circles highlight selected compounds for reference. N = 1 independent culture per cell line. **b**, Comparison of combination scores (cScore) indicating the response of KRAS^G12C^ mutant NCI-H2122 cells and KRAS^G12C^ mutant SW837 cells to combination treatment of adagrasib. NCI-H2122 and SW837 cells were treated for 72 and 144 hrs, respectively. Positive scores and empty/colored circles are explained in (a). N = 1 independent culture per cell line. **c**, (*left, top*) Western blot analysis of the KRAS^G12D-^ and KRAS^G12C-^mutant isogenic murine lung cancer cell line, LKR13, without (LKR13K) or with Lkb1 (LKR13KL) or Keap1 knockout (LKR13KK). (*right, bottom*) Effects of adagrasib or adagrasib plus BI-3406 or TNO155 in a cell viability assay in KRAS^G12C-^mutant LKR13K, LKR13KL, and LKR13KK cells. Mean percentages of cell viability relative to the DMSO-treated control are shown. Error bars = ±SD; n = 3 independent cell cultures. **d**, Activity of hallmark cancer gene sets, as analyzed by RNA-seq, in untreated KRAS^G12C^-driven tumor cell lines found generally responsive (yes; NCI-H2122, NCI-H1373, SW837, NCI-H358, LU65) or unresponsive (no; SW 1573, HOP-62, HCC-44, NCI-H2030, NCI-H1792, KYSE-410) to adagrasib combination treatments in Fig. 1c. **e**, *KRAS* expression, *MRAS* expression, and MAPK pathway activity (MAPK Pathway Activity Score [MPAS]) in responsive (yes) and unresponsive (no) cells described in (a). **d-e:** Each boxplot shows lower and upper quartile and median line is indicated. Range of whiskers: 1.5x interquartile range (two-sided Wilcoxon rank sum test, confidence interval 0.95; P value is indicated). Each cell line, characterized in Fig. 1c, was sequenced once. Source data

A high-throughput compound screen compared the antiproliferative responses induced by KRAS^G12C^i plus a panel of 179 small molecule inhibitors (Supplementary Table 1) in the KRAS^G12C^-driven NSCLC cell line, NCI-H2122. Potent additive antiproliferative effects were observed with inhibitors blocking upstream activators of KRAS, including SOS1i (BI-3406), SHP2i (TNO155, SHP099), and ErbB family inhibitors (lapatinib and afatinib), as well as inhibitors that act downstream of KRAS, such as on the MAPK pathway (for example MEK inhibitor trametinib) or in parallel pathways (for example PI3K inhibitor BYL719) (Fig. 1a). Additive effects with similar combination partners were observed in the KRAS^G12C^-driven CRC and NSCLC cell lines, SW837 (Fig. 1b) and NCI-H358 (Supplementary Table 1 and Extended Data Fig. 1a,b), respectively. Across all cell lines, we demonstrated that combination treatment of KRAS^G12C^i plus BI-3406, TNO155/SHP099 or lapatinib/afatinib induced an additive antiproliferative response.

These findings were validated using a panel of 14 KRAS^G12C^-driven human cell lines. Adagrasib plus BI-3406 (8 of 14), TNO155 (11 of 14), cetuximab (8 of 14) or the SHP2i, SHP099 (6 of 14), induced strong additive antiproliferative effects across most, and largely the same, cell lines (Fig. 1c). In SW837 and NCI-H2122 cell lines, combination with BI-3406 or TNO155 resulted in a profound and durable antiproliferative effect, whereas the strong initial response to adagrasib attenuated over time (Fig. 1d,e). Notably, 3–6 of the 14 KRAS^G12C^-driven tumor cell lines demonstrated moderate or no response to combination treatment, suggesting the presence of primary resistance (Fig. 1c). Transcriptomic analysis revealed significant increases in cancer hallmark pathways, such as epithelial to mesenchymal transition (EMT), PI3K/AKT/mTOR and transforming growth factor (TGF)β pathway signature scores, in cell lines unresponsive to combination treatments (Extended Data Fig. 1d). MAPK activity as well as *KRAS* and *MRAS* expression was similar between responder and nonresponder cell lines, although *MRAS* expression trended lower in responders (Extended Data Fig. 1e). Finally, a supervised analysis of co-occurring mutations (for example *TP53*, *KEAP1* and *STK11*; Supplementary Table 2) failed to reveal any enrichment in responder/nonresponder cell lines (Fig. 1c).

Mutational inactivation of Lkb1 (*Stk11*) or Keap1 is clinically linked to resistance to KRAS^G12C^i. Accordingly, we assessed the sensitivity of response to KRAS^G12C^i alone or plus SOS1i/SHP2i in isogenic clones of the Kras^G12D^ murine cell line, LKR13 (ref. ^26^), converted to Kras^G12C^ and lacking either Lkb1 or Keap1. We could recapitulate that Lkb1 or Keap1 loss resulted in reduced sensitivity to adagrasib treatment (Extended Data Fig. 1c). Although SOS1i nor SHP2i combination resulted in no additional benefit in the absence of Lkb1, re-sensitization of Keap1 knockout cells to adagrasib was observed (Extended Data Fig. 1c).

**Fig. 2:**
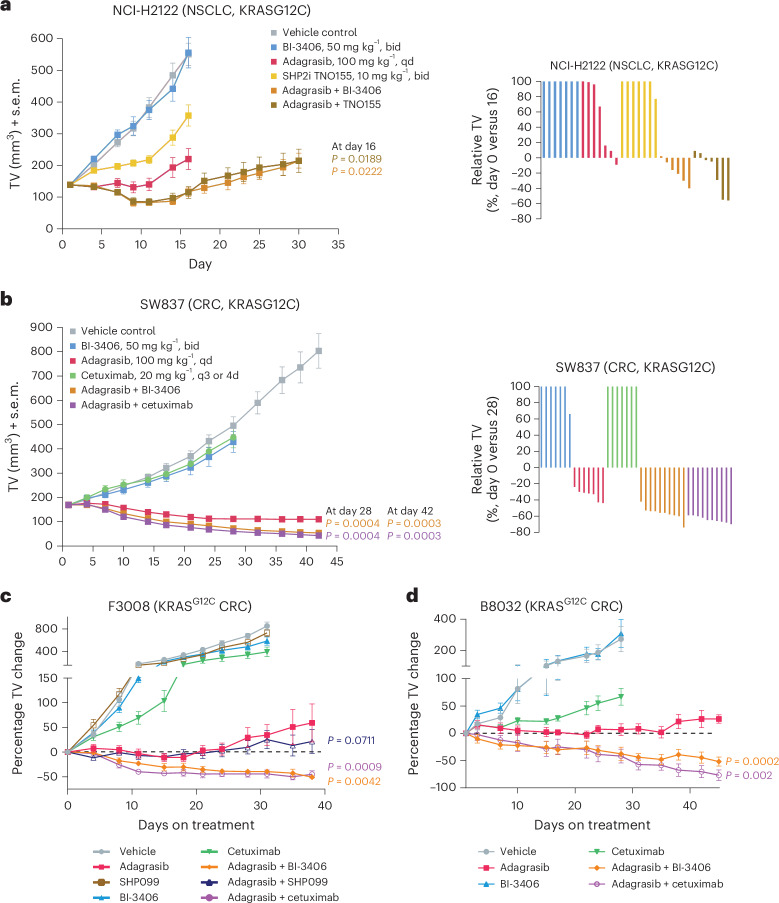
Efficacy of combination treatments in non-small cell lung cancer and colorectal cancer xenograft models. **a**, Tumor volume (TV) of mice bearing NCI-H2122 cells. Mice were treated with vehicle, BI-3406 (50 mg kg^−1^; bid, delta of 6 h; qdx5), TNO155 (10 mg kg^−1^; twice daily; qdx5), adagrasib (100 mg kg^−1^; qdx5) for 16 days, or with adagrasib plus BI-3406 or TNO155 for 30 days (left). *n* = 7 animals. On day 16, combination groups were compared to the adagrasib group using a one-sided Mann–Whitney–Wilcoxon *U*-tests adjusted for multiple comparisons using the Bonferroni–Holm Method within each subtopic. Mean ± s.e.m. shown. Relative NCI-H2122 TVs, up to one doubling (+100%), are indicated as percent change from baseline at day 16 (right). **b**, Tumor volumes of mice bearing SW837 cells (left). Mice were treated with vehicle, BI-3406 (50 mg kg^−1^; bid, delta of 6 h; qdx5), cetuximab (20 mg kg^−1^; q3 or 4d) or adagrasib (100 mg kg^−1^; qdx5) or with adagrasib plus cetuximab or BI-3406 for 42 days. *n* = 7 animals for monotherapies; *n* = 10 animals per group for combination therapies. On days 28 and 42, combination groups were compared to the adagrasib group using one-sided Mann–Whitney–Wilcoxon *U*-tests adjusted for multiple comparisons with the Bonferroni–Holm Method within each subtopic. Mean ± s.e.m. shown. Relative SW837 TVs, up to one doubling (+100%), are indicated as percent change from baseline at day 28 (right). **c**, Change in TV in NSG mice implanted with KRAS^G12C^ F3008 CRC PDX fragments and treated with vehicle, adagrasib (100 mg kg^−1^, daily), BI-3406 (50 mg kg^−1^, twice daily), SHP099 (25 mg kg^−1^, daily), cetuximab (15 mg kg^−1^, twice weekly) or adagrasib plus BI-3406, SHP099 or cetuximab. *n* = 8 animals. **d**, Change in TV in NSG mice implanted with KRAS^G12C^ B8032 CRC PDX fragments and treated with vehicle, adagrasib (100 mg kg^−1^, daily), BI-3406 (50 mg kg^−1^, twice daily), cetuximab (20 mg kg^−1^, twice weekly) or adagrasib plus BI-3406 or cetuximab. *n* = 5 animals. Comparison of combination groups to the adagrasib group was analyzed by a two-sided paired Student’s *t*-test (**c**,**d**) and resulting *P* values were adjusted for multiple comparisons with the Bonferroni–Holm method. Mean ± s.e.m. is shown. qd, once daily; bid, twice daily; q3, every 3rd day; 4d, every 4th day; qdx5, 5 days treatment per week. Source data

**Extended Data Fig. 2:**
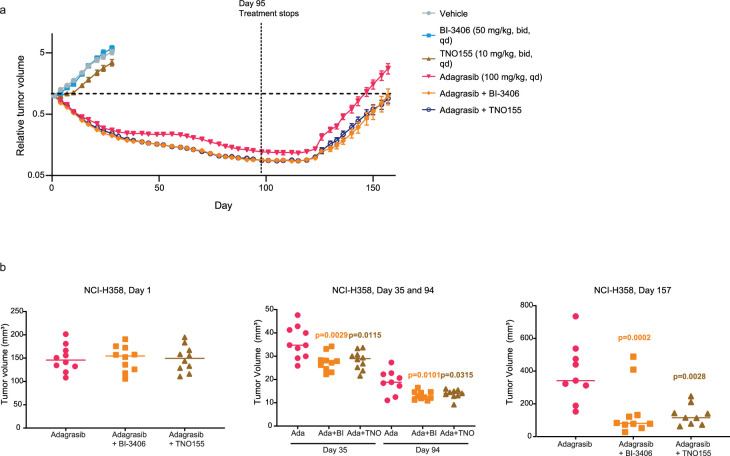
Efficacy of adagrasib single-agent and combination therapy in NCI-H358. **a**, Relative tumor volume in NCI-H358 xenograft models treated with vehicle, adagrasib (100 mg/kg, daily), BI-3406 (50 mg/kg, twice daily), TNO155 (10 mg/kg, twice weekly), or the combination of adagrasib plus BI-3406 or TNO155 for 95 days. Tumor volumes were monitored until Day 157. N = 10 animals. Mean ± SEM shown. **b**, Tumor volume of NCI-H358 models given adagrasib (ada) alone or plus BI-3406 (Ada+BI) or TNO155 (Ada+TNO) on Day 1 (*left*) or Day 35 and Day 94 (*middle*) of treatment. All mice were taken off treatment on Day 95. Tumor volume was monitored until Day 157 (*right*). Differences from the adagrasib vs combination groups were analyzed using a two-sided non-parametric Mann–Whitney-Wilcoxon U-test. Day 94 adagrasib, adagrasib+TNO155; Day 157 adagrasib, adagrasib+TNO155: n = 9 animals; all other groups: n = 10 animals. Median indicated by horizontal line. Source data

The enhanced antiproliferative effect of adagrasib plus BI-3406, TNO155 or cetuximab was further confirmed in vivo in cell line-derived xenograft (CDX) (NCI-H2122, NCI-H358 and SW837) and patient-derived xenograft (PDX) (F3008 and B8032) models. Pharmacokinetic analyses demonstrating similar exposure (relative area under the curve of combination versus single-agent within 1.35-fold) across all treatments (Supplementary Table 3), with adagrasib showing increased exposure at the 24-h time point before re-dosing, likely due to delayed gastric emptying of the combination (Supplementary Table 3). All treatments were very well tolerated (Supplementary Tables 4 and 5), with the exception of TNO155 in NSG mice; therefore, SHP099 was administered in the F3008 model. Adagrasib monotherapy resulted in modest tumor growth inhibition (TGI) (83% TGI on day 16) in the NCI-H2122 model, with initial tumor stasis followed by outgrowth (Fig. 2a), suggesting early onset of resistance. In contrast, combination treatment with adagrasib plus BI-3406 (106% TGI, the average tumor volume (TV) change from baseline (ΔTV), −18%, day 16) or TNO155 (104% TGI, average ΔTV, −13%, day 16) significantly enhanced growth inhibition (*P* = 0.0222, five out of six tumors in regression from BI-3406 combination; *P* = 0.0189, five out of seven tumors in regression from TNO155 combination) (Fig. 2a). Tumor outgrowth, albeit slow, was still observed under combination treatment (Fig. 2a). In the NCI-H358 model, treatment with combination with BI-3406 or TNO155 resulted in a 50% deeper regression compared to adagrasib monotherapy, as well as no outgrowth on treatment (Extended Data Fig. 2). In the SW837 model, combination of adagrasib plus BI-3406 (119% TGI, average ΔTV, −67.8%, day 42) or cetuximab (121% TGI, average ΔTV, −74.4%, day 42) resulted in deeper tumor regressions compared to adagrasib alone (108% TGI, average ΔTV, −35%) (Fig. 2b). Similarly, in F3008 and B8032 models, treatment with adagrasib plus BI-3406 or cetuximab resulted in prolonged tumor regression as compared to the modest antitumor activity, with tumor outgrowth observed around day 20–30 post-treatment, observed with adagrasib alone or with SHP099 (Fig. 2c,d). Overall, across all models, combination with BI-3406 led to more profound antitumor activity than induced by adagrasib alone and this was comparable to that observed upon treatment with adagrasib and SHP2i or cetuximab.

### Combination therapy enhances antitumor effects in vitro

**Fig. 3:**
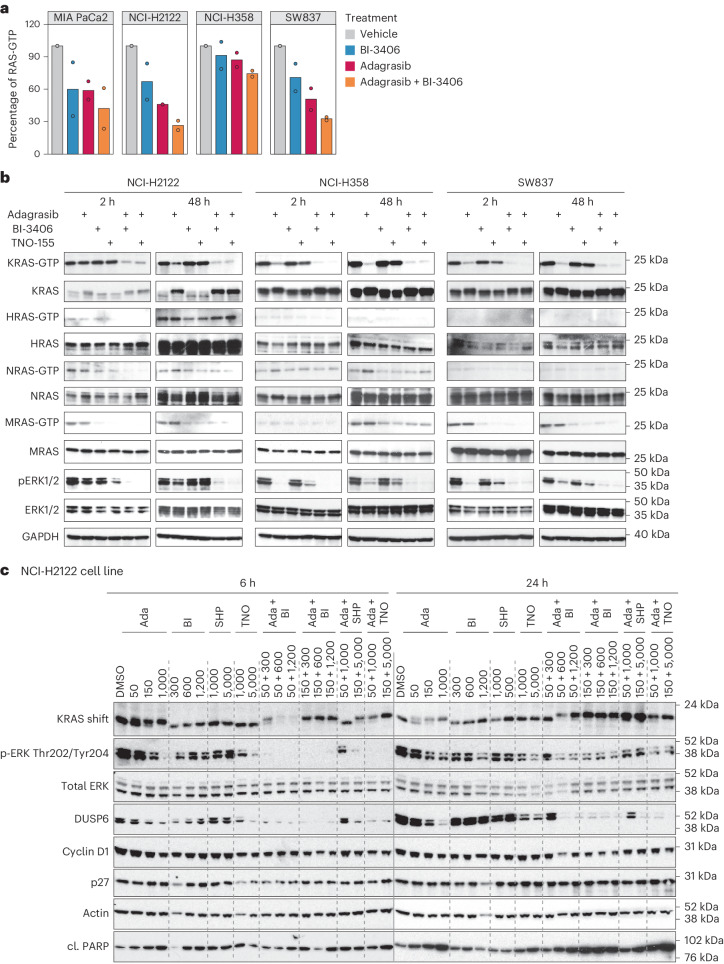
Modulation of RAS-MAPK signaling by adagrasib monotherapy or combination treatments in a KRAS^G12C^ mutant non-small cell lung cancer cell line. **a**, G-LISA plate analysis of total RAS-GTP levels across the indicated cell lines treated with vehicle (DMSO), adagrasib, BI-3406 or the combination of adagrasib plus BI-3406 for 2 h. Adagrasib concentrations used represent the IC_50_ values for each cell line: 5 nM (MIA PaCa-2), 10 nM (NCI-H358), 20 nM (SW837) or 150 nM (H2122). BI-3406 concentration was 500 nM for all cell lines. Data are shown as percentage of the vehicle control at for each cell line; *n* = 2 independent cultures. **b**, NCI-H2122, NCI-H358 and SW837 cells were treated with vehicle (DMSO), adagrasib (150 nM), BI-3406 (1200 nM), TNO155 (1000 nM), adagrasib + BI-3406 or adagrasib + TNO155 for 2 or 48 h. The cell lysates were subjected to pull down using RAF-RBD beads followed by immunoblotting for KRAS, NRAS, HRAS and MRAS. GAPDH served as the loading control. Images are representative of two independent experiments. **c**, Western blot analysis of NCI-H2122 cells treated with adagrasib, BI-3406, SHP099 or TNO155 alone or adagrasib combined with BI-3406, SHP099 or TNO155 at indicated concentrations (nM) for either 6 or 24 h. KRAS shift indicates covalent binding of compound to KRAS^G12C^ resulting in a slower migrating form of KRAS. β-Actin served as the loading control. Ada, adagrasib; BI, BI-3406; SHP, SHP099; TNO, TNO155. The image is of one experiment with the indicated compounds. The western blot was repeated with SOS1i BI-1701963 and KRAS^G12C^i BI-1823911 with similar results. DMSO, dimethylsulfoxide. Source data

The mechanistic impact of adagrasib plus BI-3406 treatment on KRAS activation and MAPK pathway activity was assessed. RAS-GTP levels were assessed in four KRAS^G12C^ cell lines receiving adagrasib, BI-3406 or in combination. Compared to monotherapies, co-treatment with BI-3406 further reduced total RAS-GTP levels (Fig. 3a), consistent with the expected activity of BI-3406 (ref. ^23^).

To determine the impact of adagrasib, BI-3406 or TNO155 alone or in combination on the activity of individual RAS isoforms, we performed active RAS pulldown experiments in three KRAS^G12C^ cell lines followed by immunoblotting for K-, N-, H- and MRAS (Fig. 3b). Treatment with adagrasib resulted in decreased levels of GTP-bound KRAS in all cell lines. Whereas, in line with previous reports^15^, increased activity of the WT RAS isoforms, including NRAS and MRAS, occurred in NCI-H2122 and NCI-H358 cells following adagrasib treatment, suggesting that upregulation of these GTPases may compensate for KRAS inhibition. In KRAS^G12C^ homozygous NCI-H2122 cells, KRAS protein levels were increased upon adagrasib treatment (Fig. 3b), indicating that upregulation of KRAS^G12C^ protein may constitute a compensatory mechanism in the absence of WT KRAS. In all three cell lines, co-treatment with BI-3406 or TNO155 deepened and prolonged the reduction in KRAS-GTP levels and counteracted upregulation of WT RAS isoform activity. In addition, both BI-3406 and TNO155 monotherapy decreased MRAS-GTP to levels below those seen in the vehicle-treated group suggesting that both inhibitors can potentiate the antitumor effect of KRAS^G12C^ inhibition through the blockade of compensatory mechanisms driven by other RAS isoforms (Fig. 3b).

**Extended Data Fig. 3:**
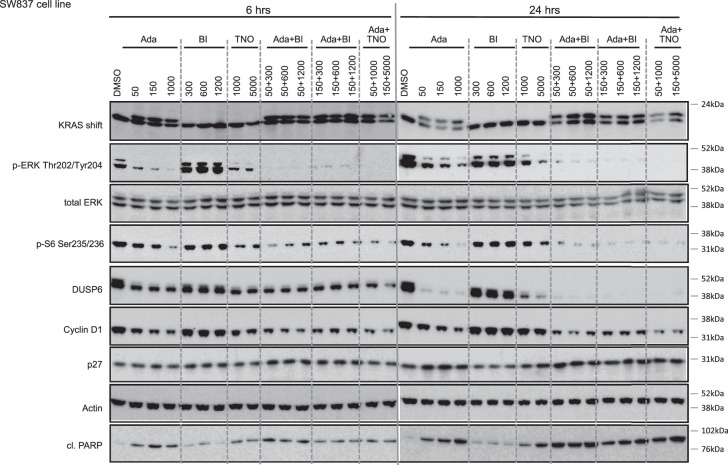
Modulation of RAS-MAPK signaling by adagrasib monotherapy or combination treatments in a colorectal cancer (CRC) cell line. Western blot analysis of the KRAS^G12C-^mutant colorectal cancer cell line, SW837, treated with adagrasib, BI-3406, or TNO155 alone, or adagrasib combined with BI-3406 or TNO155 at indicated concentrations (nM) for either 6 or 24 hours. KRAS shift indicates covalent binding of compound to KRAS^G12C^, resulting in a slower migrating form of KRAS. β-actin served as the loading control. For blots with ERK and cleaved PARP antibodies, 15 µg total protein/lane; for all other blots, 20 µg total protein/lane. Ada: adagrasib; BI: BI-3406; TNO: TNO155. Image is of one experiment with the indicated compounds. Western blot was repeated with SOS1i BI-1701963 and KRASG12Ci BI-1823911 with similar results.

The impact of combination therapies on KRAS downstream signaling was assessed. Pharmacodynamic markers of the RAS-MAPK pathway and changes in the cellular phenotype were evaluated after single-agent or combination treatment with adagrasib, BI-3406 and/or TNO155/SHP099. Study concentrations were matched to clinically relevant plasma exposures determined through clinical data^4,6,7,27^ or through preclinical and investigational new drug studies that leveraged multiple cancer models. After 6 h, adagrasib plus BI-3406 or TNO155/SHP099 combination treatment induced stronger reduction in p-ERK, compared to single agents in both cell lines (Fig. 3c and Extended Data Fig. 3). After 24 h of treatment, combination approaches or high doses of adagrasib monotherapy achieved a similar, sustained inhibition of downstream intermediates, for example DUSP6, a negative regulator of the MAPK pathway (Fig. 3c and Extended Data Fig. 3). Notably, combination therapy attenuated the rebound of p-ERK observed at 24 h in cells treated with adagrasib (Fig. 3c and Extended Data Fig. 3). These effects were associated with elevated cleaved PARP levels and a substantial reduction in cyclin D1 levels, particularly in SW837 cells after 24 h of combination treatment (Fig. 3c and Extended Data Fig. 3). Together, these findings suggest the profound suppression of RAS activation and downstream signaling induced by combination approaches was associated with cell cycle arrest and apoptosis.

### KRAS^G12C^i combination therapy inhibits RAS-MAPK signaling

Pharmacodynamic biomarker changes were correlated with tumor progression in vivo. Tumor-bearing mice were treated with either single-agent or combination treatments for 7 (24 h time point) to 8 (4 and 48 h time points) consecutive days, with samples collected at 4, 24 and 48 h after the last dose.

**Fig. 4:**
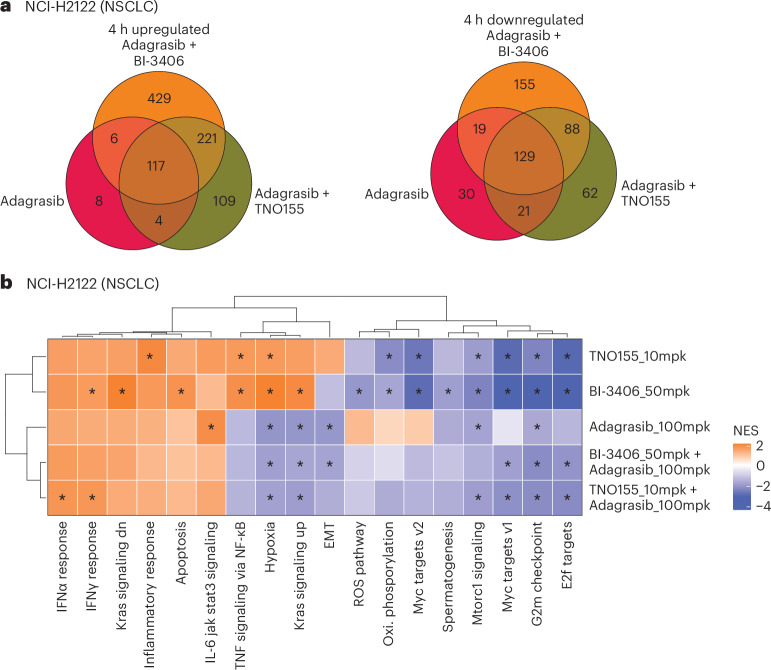
Differential gene expression induced by single-agent or combination treatments in vivo in a KRAS^G12C^-mutant non-small cell lung cancer xenograft model. All treatments were administered for 7 consecutive days, and samples were collected after the last dose. **a**, Venn diagram showing the overlap of upregulated (left) or downregulated (right) differentially expressed genes in NCI-H2122 xenograft models treated with adagrasib (100 mg kg^−1^) alone or combined with BI-3406 (50 mg kg^−1^) or TNO155 (10 mg kg^−1^) at 4 h post-last dose. *n* = 4 animals. **b**, Heatmap showing the normalized enrichment score of hallmark pathway gene sets in tumors from KRAS^G12C^-mutant NSCLC xenograft model (NCI-H2122) xenograft models treated with indicated single or combination therapies at 4 h post-last dose. NES, normalized enrichment score. Stars on the heatmap indicate adjusted *P* value < 0.05. Nominal *P* values are obtained by GSEA and adjusted for multiple comparisons using the Benjamini–Hochberg method. *n* = 4 animals. Source data

**Extended Data Fig. 4:**
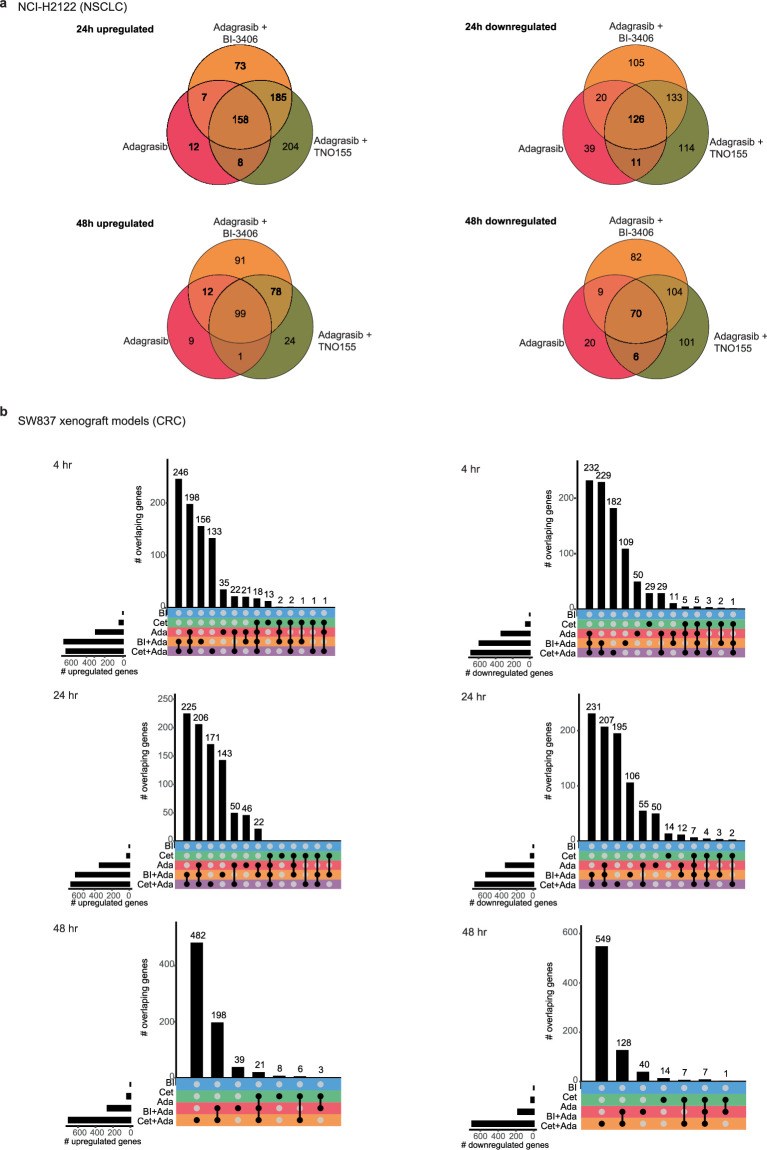
Gene modulation by single-agent or combination treatments in KRAS^G12C^–driven non-small cell lung cancer (NSCLC) and colorectal cancer (CRC) xenograft models. **a**, Venn diagram showing the overlap of upregulated (left) or downregulated (right) differentially expressed genes in NCI-H2122 xenograft models treated with adagrasib (100 mg/kg) alone or combined with BI-3406 (50 mg/kg) or TNO155 (10 mg/kg) at 24 and 48 hours post-last dose. N = 4 animals. **b**, All treatments in SW837 xenograft models were administered over seven consecutive days. Overlap of upregulated (left) or downregulated (right) genes in tumors from SW837 xenograft models across three time points after receiving one of these treatments: 50 mg/kg bid, p.o. BI-3406 (BI); 20 mg/kg twice a week, i.p. cetuximab (Cet); 100 mg/kg qd, p.o. adagrasib (Ada); 50 mg/kg BI-3406 plus 100 mg/kg adagrasib (BI + Ada); 20 mg/kg cetuximab plus 100 mg/kg adagrasib (Cet + Ada). Samples were collected at 4, 24, and 48 hours after the last dose. Sample sizes are the same as Extended Data Fig. 5b. Source data

**Extended Data Fig. 5:**
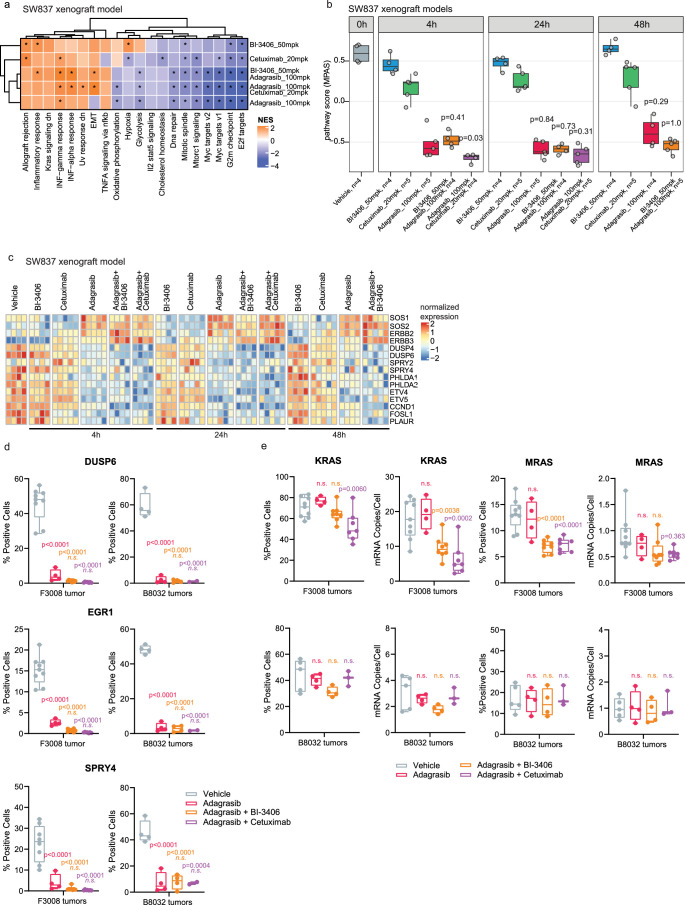
Differentially modulated pathways in a KRAS^G12C^–driven colorectal cancer (CRC) xenograft model. **a**, Heatmap showing the normalized enrichment score (NES) of hallmark pathway gene sets in tumors from SW837 xenograft models treated with indicated single or combination therapies at 4 hours post-last dose. Asterisks on the heatmap indicate adjusted p value < 0.05. Nominal p values are obtained by gene set enrichment analysis (GSEA) and adjusted for multiple comparisons using Benjamini–Hochberg method. Number of animals per group is same as Extended Data Fig. 5b. **b**, Modulation of overall MAPK pathway activity, as indicated by the MAPK Pathway Activity Score (MPAS), in tumors from SW837 xenograft models treated with indicate single-agent or combination treatments. Samples were collected at 4, 24 and 48 hours after the last dose. Boxplots show lower and upper quartiles and median line is indicated. Whiskers: 1.5 x interquartile range. Data analyzed by two-sided Wilcoxon rank sum test. Number of animals per group indicated on graph. **c**, Differential modulation of selected MAPK pathway genes in SW837 xenograft models treated with indicated single-agent or combination treatments. Samples were collected at 4, 24, or 48 hours post last dose. Sample sizes/group reported in b. Color legend: normalized expression. Number of animals per group is same as Extended Data Fig. 5b. **d,e**, RNAscope analyses of *DUSP6*, *EGR1*, and *SPRY4* expression (d) as well as *MRAS* and *KRAS* expression (e) in CRC PDX models, F3008 and B8032, at the end of tumor growth curve experiments shown in Fig. 2c,d, respectively. Comparisons to vehicle (normal text) or adagrasib group (italicized text) analyzed by one-way analysis of variance followed with Holm-Šídák’s multiple comparisons test; p < 0.05 indicates significance; n.s. = non-significance. Boxplots show lower and upper quartiles and median line is indicated. Whiskers: 1.5 x interquartile range. F3008 or B8032: n = 8 or n = 5 tumors/group, respectively. Source data

Differential gene expression analysis was first conducted on NCI-H2122 xenografts treated with adagrasib alone or plus BI-3406 or TNO155 and SW837 xenografts treated with adagrasib alone or plus BI-3406 or cetuximab. In the NCI-H2122 model, adagrasib plus BI-3406 induced the strongest overall transcriptomic change at 4 h (Fig. 4a) and 48 h (Extended Data Fig. 4a). In SW837 xenografts, adagrasib plus BI-3406 or cetuximab induced the strongest overall transcriptomic changes across all time points, with adagrasib plus cetuximab inducing stronger transcriptomic modulation compared to adagrasib plus BI-3406 (Extended Data Fig. 4b). Across all time points in both models, the largest overlap of deregulated genes, which included genes associated with the MAPK family, tumor growth and cancer progression^28–30^, was identified between adagrasib plus BI-3406 and adagrasib plus TNO155 combination treatments, suggesting similarly induced transcriptomic modulation (Fig. 4a, Extended Data Fig. 4 and Supplementary Tables 6 and 7). Follow-up gene set enrichment analysis (GSEA) revealed downregulation of pathways associated with cell growth and survival, EMT, targets of oncogenic transcription factor *MYC*, as well as genes upregulated by active *KRAS* in NCI-H2122 tumors treated with combination therapies (false discovery rate (FDR) < 0.01) (Fig. 4b). Similarly, GSEA showed downregulation of pathways associated with cell growth and survival, as well as *MYC* target pathways, in SW837 tumors treated with single-agent and combination therapies (Extended Data Fig. 5a).

**Fig. 5:**
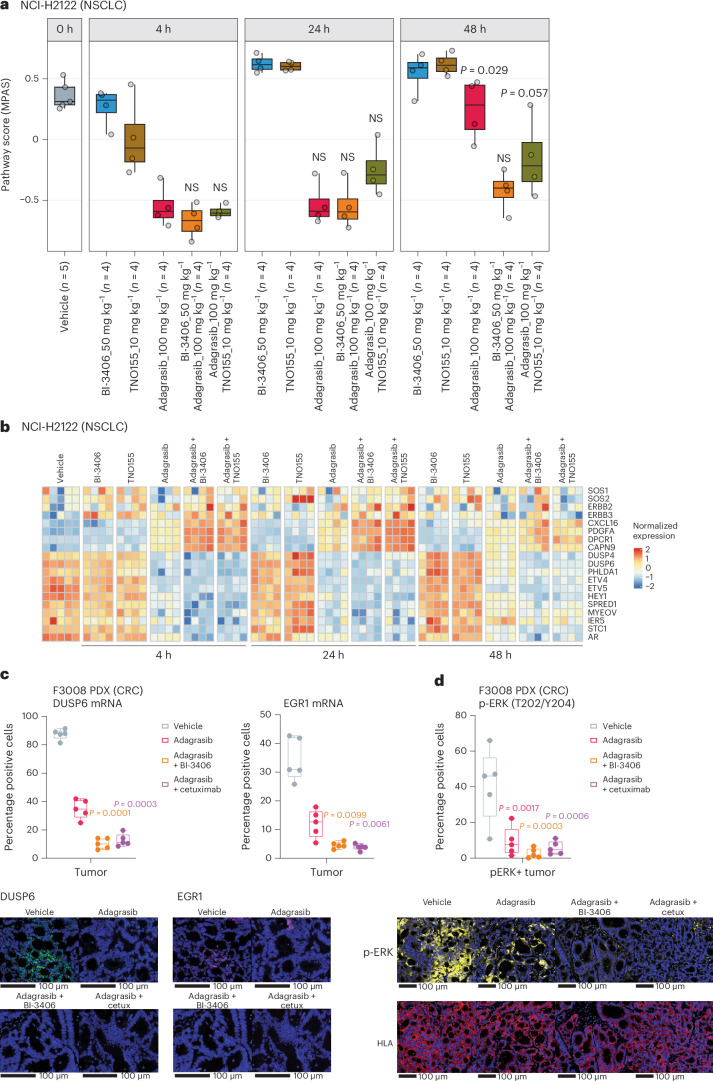
Differential modulation of single-agent or combination treatments in vivo on MAPK signaling and other pathways. **a**, Modulation of overall MAPK pathway activity, as indicated by the MPAS, in NCI-H2122 xenograft models treated with indicated monotherapies or combination treatments at 4, 24 or 48 h post-last doses. *n* = 4 animals. Boxplots show low and upper quartiles and median line is indicated. Whiskers, 1.5 × interquartile range. Data were analyzed by a two-sided Wilcoxon rank-sum test. MPAS scores for adagrasib alone or in combination at 24 and 48 h post-last dose were compared against that of adagrasib at 4 h post-last dose. **b**, Differential modulation of select MAPK pathway genes in NCI-H2122 xenograft models treated with indicated single-agent or combination treatments. Tumors were collected at 4, 24 or 48 h post-last dose. *n* = 4 tumors per time point. Color legend, normalized expression. **c**, Percentage of positive tumor cells expressing *DUSP6* or *EGR1* mRNA analyzed by RNAscope in F3008 CRC PDX models treated with vehicle, adagrasib (100 mg kg^−1^, daily), adagrasib plus BI-3406 (50 mg kg^−1^, twice daily) or adagrasib plus cetuximab (15 mg kg^−1^, twice weekly) for 5 days (top). Tumors were collected 4 h after the last dose. *n* = 5 animals. Boxplots show low and upper quartiles and median line is indicated. Whiskers indicate 1.5 × interquartile range. Data analyzed by two-sided Student’s *t*-test. Representative images from RNAscope analysis of *DUSP6* and *EGR1* expression in tumor tissue from models described in the top (bottom). Scale bar, 100 µm. **d**, Percentage of positive tumor cells expressing phosphor-ERK as analyzed by multiplex immunofluorescence in the same tumor tissues as described in **c**. *n* = 5 animals. Boxplots show low and upper quartiles and median line is indicated. Whiskers indicate 1.5 × interquartile range. Comparisons to vehicle analyzed by two-sided Student’s *t*-test. Representative images from multiplex immunofluorescence analysis of p-ERK and HLA in tumor tissue from models described in the top (bottom). Scale bar, 100 µm. NS, not significant. Source data

Subsequently, the effects of adagrasib monotherapy and combination treatments on MAPK signaling and associated pathways were assessed. The overall MAPK pathway activity of individual tumors was first quantified with MAPK Pathway Activity Score (MPAS) analysis^31^ on transcriptomic data. In NCI-H2122 tumors, treatment with adagrasib alone (median MPAS = −0.59) or plus BI-3406 (median MPAS = −0.67) or TNO155 (median MPAS = −0.61) downregulated MAPK activity to a similar extent at 4 h post-last dose (Fig. 5a). At 48 h post-last dose, sustained suppression of MAPK activity was only observed in NCI-H2122 tumors treated with adagrasib plus BI-3406, with a significant (*P* = 0.029) or moderate (*P* = 0.057) rebound of MAPK activity in those treated with, respectively, adagrasib or adagrasib plus TNO155 (Fig. 5a). Of note, the latter may be at least partially explained by plasma clearance of both adagrasib and TNO155 at the 24 h time point (Supplementary Table 8). Both combination treatments also achieved greater downregulation of genes related to the MAPK pathway (for example, *DUSP4*, *DUSP6* and *SPRED1*) at 4, 24 and 48 h time points (Fig. 5b and Supplementary Table 6). In SW837 tumors, adagrasib alone (median MPAS score = −0.57) or plus BI-3406 (median MPAS score = −0.49) or cetuximab (median MPAS score = −0.68) all downregulated overall MAPK activity as well as genes associated with MAPK signaling at 4 h (Extended Data Fig. 5b,c); however, only combination approaches were able to sustain downregulation of MAPK pathway activity, including suppressing the expression of *DUSP6* (log_2_ fold change compared to control, −2.4 fold) and *CCND1* (log_2_ change compared to control, −1.8 fold), for up to 48 h (Extended Data Fig. 5b,c and Supplementary Table 7). Consistently, in CRC PDX models, analysis by RNAscope in situ hybridization assay revealed downregulation of *DUSP6* and *EGR1* upon adagrasib treatment and further downregulation upon combination treatment (Fig. 5c,d). Analyses of residual tumors from these PDX models (Fig. 2c,d) found that all treatments downregulated *DUSP6*, *EGR1* and *SPRY4* expression to similar degrees (Extended Data Fig. 5d). In the F3008 model, when compared to adagrasib treatment alone, treatment with adagrasib plus BI-3406 or cetuximab resulted in a strong downregulation of *KRAS* and *MRAS* expression (Extended Data Fig. 5e), suggesting durable inhibition of MAPK pathway signaling in residual tumor cells.

**Extended Data Fig. 6:**
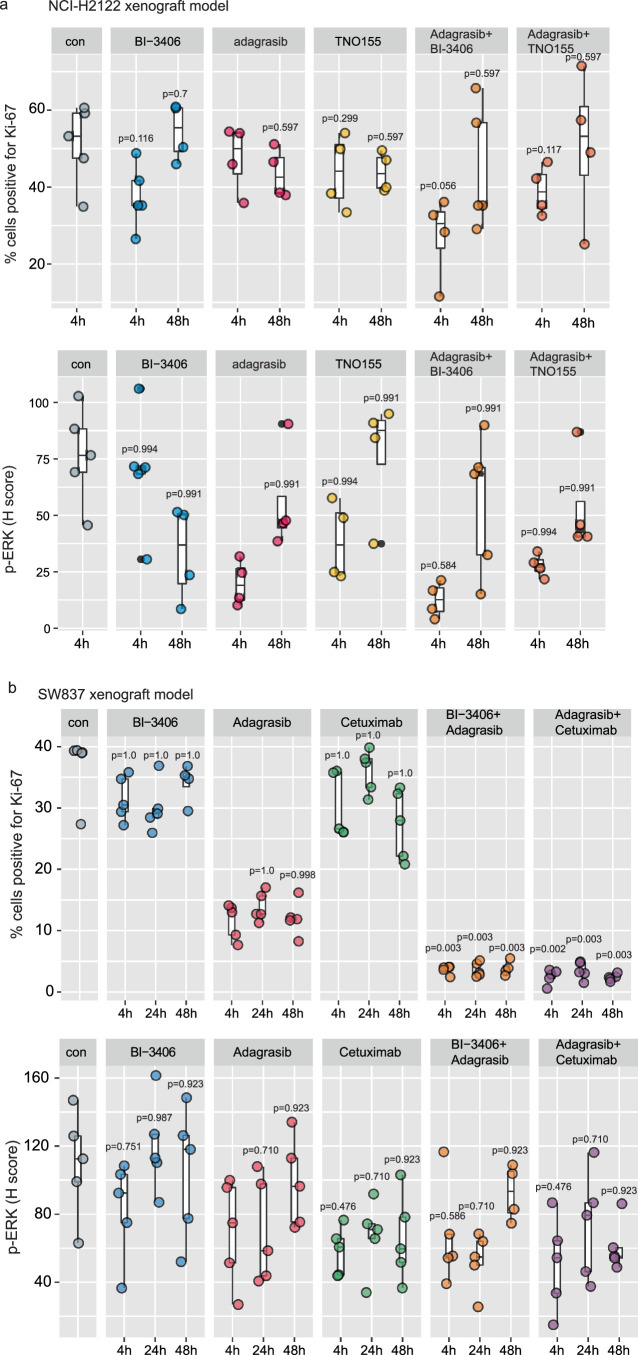
Biomarker analysis in KRAS^G12C^–driven non-small cell lung cancer (NSCLC) or KRAS^G12C^–driven colorectal cancer (CRC) tumors treated with single-agent or combination treatments. All treatments were administered over 7 consecutive days, with samples collected on Day 7 and 8 after the last dose. **a**, Biomarker analyses of cell proliferation biomarker, Ki-67 (top), and phosphor-ERK (p-ERK) (bottom), in NCI-H2122 xenograft models treated with vehicle (con), BI-3406 (50 mg/kg, bid, p.o.), adagrasib (100 mg/kg, qd, p.o.), TNO155 (10 mg/kg, bid, p.o.), adagrasib (100 mg/kg) plus BI-3406 (50 mg/kg), adagrasib (100 mg/kg) plus TNO155 (10 mg/kg) at 4 or 48 hours post-last dose. Control animals were treated with vehicle. Boxplots show low and upper quartiles and median line is indicated. Whiskers: 1.5 x interquartile range. Data analyzed by one-sided t-test with resulting p values adjusted for multiple comparisons using the Benjamini–Hochberg method within each subtopic. n = 5 tumors/group. **b**, Biomarker analyses of cell proliferation biomarker, Ki-67 (top), and phosphor-ERK (p-ERK) (bottom), in SW837 xenograft models treated with vehicle (con), BI-3406 (50 mg/kg, bid, p.o.), adagrasib (100 mg/kg, qd, p.o.), TNO155 (10 mg/kg, bid, p.o.), adagrasib (100 mg/kg) plus BI-3406 (50 mg/kg), adagrasib (100 mg/kg) plus TNO155 (10 mg/kg) at 4 or 48 hours post-last dose. Control animals were treated with vehicle. Boxplots show low and upper quartiles and median line is indicated. Whiskers: 1.5 x interquartile range. Data analyzed by one-sided t-test with resulting p values adjusted for multiple comparisons using the Benjamini–Hochberg method within each subtopic. n = 6 and n = 5 tumors/group. Source data

Immunohistochemistry analysis was performed to investigate whether downregulation of RAS-MAPK signaling may have contributed to the enhanced antiproliferative effects induced by combination approaches in NCI-H2122 and SW837 cell lines (Figs. 1 and 3c and Extended Data Fig. 3), xenograft models (Fig. 2a,b) and CRC PDX models (Fig. 2c,d). In NCI-H2122 xenografts, only combination with BI-3406 induced a significantly stronger downregulation of cell proliferation biomarker, Ki-67 (*P* = 0.056), when compared to levels by adagrasib monotherapy at 4 h post-last dose (Extended Data Fig. 6a). No further downregulation of Ki-67 nor p-ERK was induced by combination treatments, when compared to that by adagrasib monotherapy, in this model at 4 or 48 h post-treatment (Extended Data Fig. 6a). In SW837 tumors, combination with either BI-3406 (*P* = 0.003 at 4, 24 and 48 h) or cetuximab (*P* = 0.002, 0.003 and 0.003 for 4, 24 and 48 h, respectively) further downregulated Ki-67 when compared to levels by adagrasib alone at 4 h after the last dose (Extended Data Fig. 6b); however, no treatment further decreased p-ERK levels when compared to levels induced by adagrasib alone at 4 h in this model (Extended Data Fig. 6b), which is consistent with multiplex immunofluorescence results in the F3008 CRC PDX model (Fig. 5d).

Although combination approaches induced enhanced antiproliferative effects, a moderate rebound of MAPK pathway activity, which might be associated with adaptive resistance to KRAS^G12C^ targeted therapy^13,16^, was observed in all models by 48 h after treatment. Specifically, a significant rebound of MAPK pathway activity was observed in NCI-H2122 tumors treated with adagrasib monotherapy (median MPAS = 0.3), but not adagrasib plus BI-3406 (median MPAS = −0.67), at 48 h (Fig. 5a). In NCI-H2122 tumors treated with adagrasib plus TNO155, only a partial rebound of MAPK signaling (median MPAS = −0.61) was observed at 48 h (Fig. 5a) even though both compounds had been largely metabolized in the plasma by 48 h post-treatment (Supplementary Table 8). On the molecular level, upregulation of *SOS1*, *SOS2* and RTKs (for example *ERBB2* and *ERBB3*), as well as *CXCL16* and *PDGFA*, both associated with activation of the MAPK and/or PI3K signaling^32,33^, was observed as early as 4 h post-last dose of combination treatment (Fig. 5b). In SW837 xenografts treated with adagrasib alone or plus BI-3406 or cetuximab, a rebound in MAPK pathway activity was not observed by 48 h (Extended Data Fig. 5b) but, consistent with NCI-H2122 tumors (Fig. 5b), upregulation of *SOS1*, *SOS2* and RTKs was observed at 4 h post-last dose (Extended Data Fig. 5c). Additionally, a partial rebound of p-ERK levels was observed at 48 h post-treatment of adagrasib alone or plus BI-3406 in SW837 xenograft models (Extended Data Fig. 6b). Our findings reveal an elevation of upstream activators (for example *CXCL16*, *ERBB2/3* and *SOS1/2*) of MAPK signaling in both NCI-H2122 and SW837 models at 4 and 24 h post-last dose of combination treatment, whereas the expression of MAPK downstream signaling effectors (for example *DUSP6* and *ETV4/5*) remained suppressed at both time points and only began to rebound at 48 h post-treatment in NCI-H2122 tumors (Fig. 5a,b and Extended Data 5b,c).

Our findings underscore the necessity of co-administering adagrasib with inhibitors targeting regulators of RAS-GTP-loading to improve the efficacy and durability of KRAS^G12C^ targeted therapy. Specifically, when compared to single-agent approaches, combination treatment induced a more profound gene modulation in vivo that was accompanied by strengthened inhibition of the RAS-MAPK pathway, leading to enhanced antitumor efficacy.

### Co-targeting SOS1 addresses resistance to KRAS^G12C^i

As rapid resistance to KRAS^G12C^i monotherapy was observed in NSCLC and CRC models in vitro (Figs. 1d,e and 3c and Extended Data Fig. 3) and in vivo (Figs. 2 and 5b and Extended Data Figs. 5b,c and 6b), we sought to investigate whether combination therapy with SOS1i could be leveraged to induce antitumor effects in tumors with acquired KRAS^G12C^i resistance.

**Extended Data Fig. 7:**
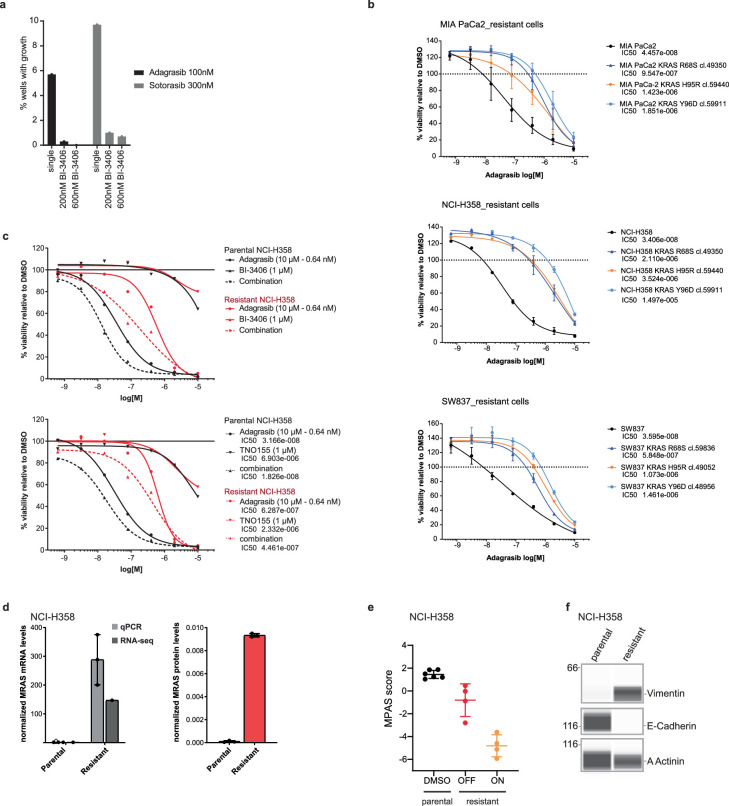
Evaluating mechanisms underpinning adagrasib resistance *in vitro.* **a**, Growth response of BaF/3 cells harboring a KRAS^G12C^ single site variant library to KRAS^G12C^-specific inhibitors alone, adagrasib (100 nM) or sotorasib (300 nM), or combined with 200 nM or 600 nM of BI-3406. n = 1 experiment. **b**, Cell viability following adagrasib treatment on MIA PaCa-2, NCI-H358, or SW837 parental cells or clones (cl) harboring additional mutations at KRAS position R68S, H95R or Y96D. Mean percentages of cell viability relative to the DMSO-treated control are shown. Error bars indicate mean ± SD; NCI-H358 KRAS H95R clones: n = 1 cell cultures; all other parental cell lines or clones: n = 2 independent cell cultures. Each group is representative of at least 2 independent cell cultures, with the exception of NCI-H358 KRAS H95R cl. 59440 (n = 1) **c**, Effects of adagrasib, BI-3406, or adagrasib plus BI-3406 (*top*) or adagrasib, TNO155, or adagrasib plus TNO155 (*bottom*) in a cell viability assay in adagrasib-sensitive (parental) or adagrasib-resistant NCI-H358 cells. Mean percentages of cell viability relative to the DMSO-treated control are shown. Each graph is representative of one of n = 2 independent cell cultures. **d**, *MRAS* expression (*left*) and protein levels (*right*) in parental (DMSO) and adagrasib-resistant NCI-H358 cell pool. Left, n = 2 individual cell cultures; right, n = 3 independent cell cultures. Error bars indicate mean ± SD. **e**, MAPK pathway activity, as indicated by MPAS score, in parental NCI-H358 cells treated with DMSO control or adagrasib-resistant NCI-H358 cells without drug (OFF) or treated with adagrasib (300 nM) (ON). DMSO group: n = 6 independent cell cultures; other groups: N = 4 independent cell cultures. **f**, Protein expression analysis of vimentin and E-cadherin, two EMT markers in parental and adagrasib-resistant NCI-H358 cells. Source data

The effectiveness of combination therapy in preventing outgrowth due to the acquisition of secondary KRAS mutations implicated in resistance was assessed^9,10,34^. KRAS^G12C^ Ba/F3 transgenic cell pools harboring every possible change at each amino acid position of the *KRAS* gene were generated and screened with KRAS^G12C^i alone or combined with escalating doses of BI-3406. When compared to KRAS^G12C^i alone, BI-3406 combination therapy resulted in a strong reduction in the number of outgrowth-positive wells that had been seeded with random library sub-pools in a dose-dependent manner (Extended Data Fig. 7a). These findings indicated that combination with BI-3406 can strengthen the antiproliferative response to *KRAS*^*G12C*^ inhibitors by impeding the outgrowth of *KRAS*^*G12C*^-secondary mutated resistant clones.

We then addressed whether combination therapy can overcome acquired resistance to KRAS^G12C^i mediated by specific on-target mutations identified in the clinic. Three KRAS^G12C^ solid cancer cell lines (NCI-H358, SW837 and MIA PaCa-2) were engineered to harbor one of most common secondary variants (*KRAS* R68S, H95Y or Y96D) associated with KRAS^G12C^i treatment. As expected, all three mutations conferred resistance to adagrasib monotherapy, as indicated by the shift in half-maximum inhibitory concentration (IC_50_) when compared to parental cells (Extended Data Fig. 7b and Supplementary Table 9). Co-administration with BI-3406 or TNO155 did not elicit additional benefit, indicating that, once established, on-target mutations that prevent adagrasib binding are not effectively addressed by these combination approaches.

Subsequent studies evaluated the potential of leveraging combination therapy to overcome acquired resistance to KRAS^G12C^i in vitro. Long-term culture in high-dose adagrasib (10 × IC_50_) was used to generate adagrasib-resistant NCI-H358 cells (NCI-H358R), which were confirmed to retain KRAS^G12C^ and did not acquire any mutations associated with resistance in patients^9,11^ (Supplementary Table 10). NCI-H358R cells demonstrated lower sensitivity to adagrasib than parental NCI-H358 cells (Extended Data Fig. 7c). Further, NCI-H358R cells were more sensitive to adagrasib plus BI-3406 or TNO155 treatment compared to adagrasib alone, although this sensitivity was not as strong as the response of parental cells to adagrasib monotherapy (Extended Data Fig. 7c). Consistent with previous reports^14^, upregulation of *MRAS* expression was observed in NCI-H358R cells (Extended Data Fig. 7d). In addition, as illustrated by increased vimentin and decreased E-cadherin protein levels (Extended Data Fig. 7f), NCI-H358R cells were observed to undergo EMT, a mechanism associated with resistance to MAPK-targeted therapy^35^. Together, these data suggest a role for MRAS, but not reactivation of MAPK signaling (Extended Data Fig. 7e), in driving acquired resistance to adagrasib in KRAS^G12C^-mutated NSCLC cells, and that adagrasib plus BI-3406 combination treatment may be effective within this context.

**Fig. 6:**
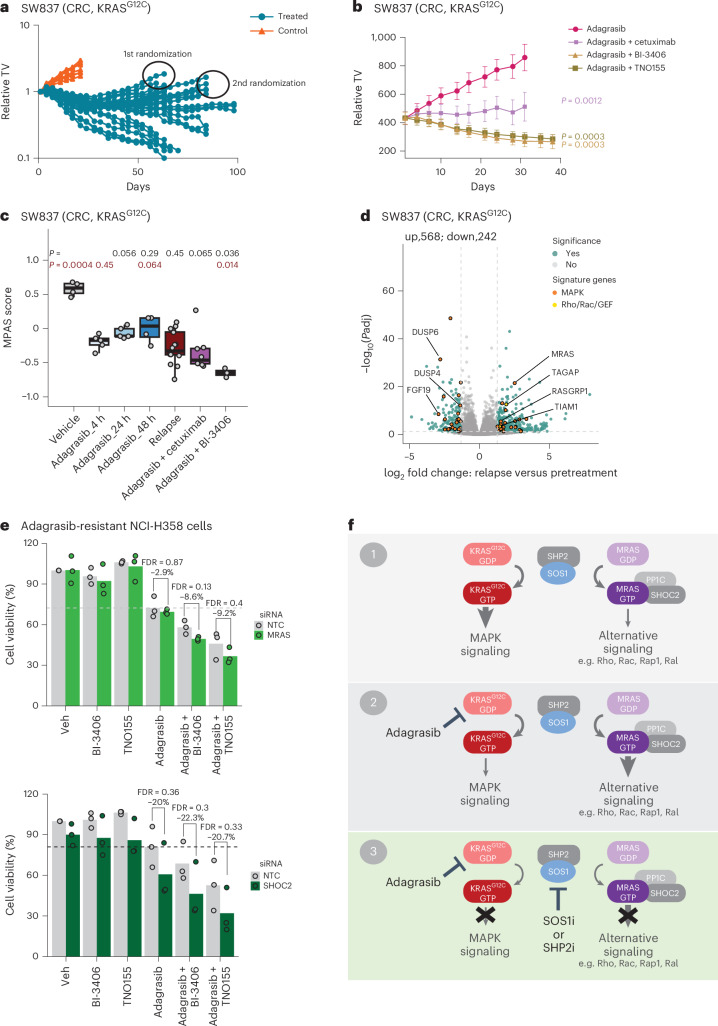
Adagrasib-resistant models remained sensitive to an adagrasib plus SOS1i combination. **a**, Tumor growth in the KRAS^G12C^-mutant CRC xenograft model (SW837) treated with vehicle (orange) or adagrasib (blue, 50 mg kg^−1^). *n* = 7 control mice and *n* = 30 out of 300 mice (Extended Data Fig. 8a) treated with adagrasib are shown. **b**, Outgrowing adagrasib-resistant tumors from **a** that had increased in size to ≤100 mm³ were treated with adagrasib (50 mg kg^−1^, 5 times per week, orally), adagrasib plus BI-3406 (50 mg kg^−1^, twice daily, orally), adagrasib plus cetuximab (20 mg kg^−1^, twice weekly, i.p.) or adagrasib plus TNO155 (10 mg kg^−1^, twice daily). *n* = 5 animals per group. Mean ± s.e.m. are shown. Combination groups were compared to adagrasib group on day 31 using a one-sided Mann–Whitney–Wilcoxon *U*-tests adjusted for multiple comparisons according to the Bonferroni–Holm Method within each subtopic. **c**, Modulation of overall MAPK pathway activity, as indicated by the MPAS, in tumors derived from SW837 xenograft models. Tumors were collected from models before adagrasib treatment (pretreatment) and during relapse as well as from adagrasib-resistant models treated with adagrasib plus BI-3406 or cetuximab. Boxplots show low and upper quartiles and median line is indicated. Whiskers indicate 1.5 × interquartile range. Data were analyzed by a two-sided Wilcoxon rank-sum test. Bold *P* values indicate comparison to adagrasib_4 h group; italicized red *P* values indicate comparison to relapse group. Vehicle, *n* = 4 animals; adagrasib_4 h, *n* = 5 animals; adagrasib_24 h, *n* = 5 animals; adagrasib_48 h, *n* = 4 animals; relapse, *n* = 16 animals; adagrasib plus cetuximab, *n* = 8 animals; adagrasib plus BI-3406, *n* = 3 animals. **d**, Volcano plot of differentially regulated genes between pre-adagrasib treatment (pretreatment) and relapsed SW837 tumors. The most significantly differential genes involved in MAPK pathway are highlighted. *P* values were obtained by two-sided Wald test and adjusted for multiple comparisons using Benjamini–Hochberg method. *n* = 6 and 16 tumors in pre-treatment and relapse groups, respectively. **e**, Cell viability of adagrasib-resistant NCI-H358 cells transfected with siRNA targeting MRAS (top) or SHOC2 (bottom) or non-targeting control (NTC) and treated with control or indicated single-agent or combination therapies. A dashed line was added to enable visual comparison to MRAS-/SHOC2-expressing cells treated with adagrasib monotherapy. Data were analyzed using a one-sided *t*-test with *P* values adjusted for multiple comparisons using Bonferroni method. *n* = 3 independent cell cultures. **f**, Schematic representing the putative mechanism of resistance to KRAS^G12C^i and overcoming resistance by KRAS^G12C^i and SOS1i combination. i.p., intraperitoneally. Source data

**Extended Data Fig. 8:**
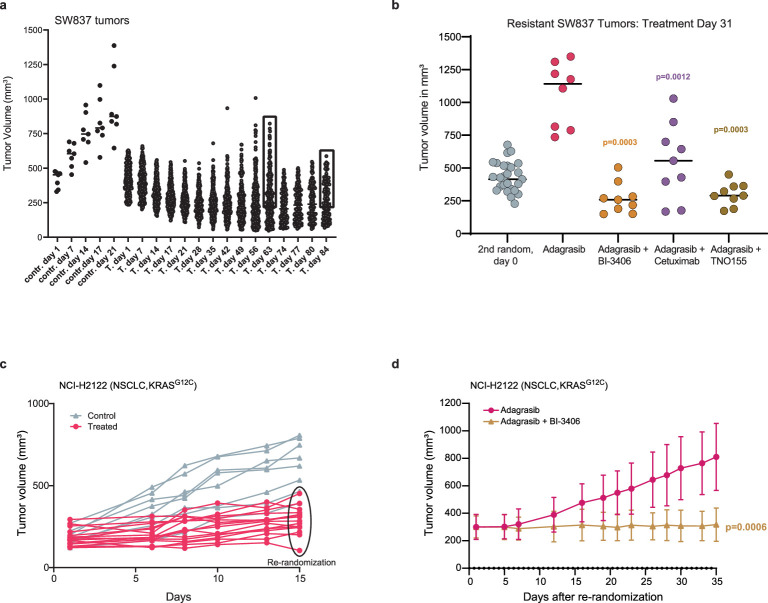
Combination treatments elicit responses from KRAS^G12C^i-resistant colorectal cancer (CRC) and non-small cell lung cancer (NSCLC) models. **a**, Growth of SW837 tumors treated long-term with adagrasib (50 mg/kg; n = 300 mice total) or vehicle (0.5% Natrosol + 0.5% DMSO; n = 7 mice) on a 5 days on/2 days off dosing schedule. Outgrowth indicating tumors that developed acquired resistance to adagrasib is indicated with a black rectangle. **b**, Growth of adagrasib-resistant SW837 tumors (n = 35 animals) from (a) that were further treated with the following for 31 days: adagrasib (50 mg/kg, qd, p.o.,), adagrasib plus BI-3406 (50 mg/kg, bid, p.o.), adagrasib plus cetuximab (20 mg/kg, q3 or 4d, i.p.), or adagrasib plus TNO155 (10 mg/kg, bid). Bar indicates median. Data were analyzed on Day 31 by an one-sided non-parametric Mann–Whitney-Wilcoxon U-tests adjusted for multiple comparisons according to the Bonferroni–Holm Method within each subtopic. Day 0 group, n = 35 mice; adagrasib group, n = 8 mice; other groups, n = 9 mice. ada = adagrasib; n.s. = non-significant. **c**, Individual tumor volumes of NCI-H2122 xenograft mice treated with vehicle or adagrasib (100 mg/kg, daily) for 15 days. On Day 15, adagrasib-resistant tumors (circled) were then re-randomized for second-line treatment of either continued treatment with adagrasib alone or adagrasib combined with BI-3406. Day 15 was selected for a 2nd randomization as, at this time point, several adagrasib-treated NCI-H2122 tumors started to grow again. N = 8 mice in the vehicle group, N = 16 mice in adagrasib-treated group. **d**, Tumor volume (Mean ± SD) of NCI-H2122 models given adagrasib alone or plus BI-3406 for 35 days after re-randomization from (b). N = 8 mice in adagrasib group, N = 7 mice in combination therapy group. Statistical differences were analyzed using two-sided t-test. Source data

We next evaluated whether combination with BI-3406 was effective in re-establishing sensitivity in adagrasib-resistant tumors. SW837 xenograft tumors were treated with adagrasib, resulting in an initial phase of tumor control followed by outgrowth, with regrowth of more than 100 mm³ representing acquired resistance (Fig. 6a and Extended Data Fig. 8a). Relapsed SW837 tumors were then randomized on day 63 and 84 for retreatment with adagrasib alone or plus BI-3406, cetuximab or TNO155. In relapsed SW837 tumors, treatment of adagrasib plus BI-3406 or TNO155, but not adagrasib alone, resulted in tumor regression (Fig. 6b and Extended Data Fig. 8b). Tumor stasis was observed in models treated with adagrasib plus cetuximab (Fig. 6b). Adagrasib-resistant NCI-H2122 xenograft tumors were then generated using a similar method, with relapsed NCI-H2122 tumors randomized on day 15 for second-line treatment with adagrasib alone or plus BI-3406 (Extended Data Fig. 8c). In relapsed NCI-H2122 tumors, treatment with adagrasib plus BI-3406 resulted in tumor stasis (Extended Data Fig. 8d). All treatments were well tolerated (Supplementary Table 11). These findings suggest that combinations with BI-3406 or TNO155 can be effective at re-establishing tumor growth control in tumors with acquired resistance to adagrasib.

The molecular mechanism underpinning the strong antiproliferative response observed in relapsed SW837 xenograft models upon KRAS^G12C^i plus SOS1i combination therapy was then investigated. Transcriptomic analysis of tumors explanted at the end of study, however, did not identify secondary *KRAS* mutations, increased *KRAS* expression, or genomic aberrations, previously reported in tumors with acquired resistance to KRAS^G12C^i (refs. ^9,11^) (Supplementary Table 12). MAPK pathway activity in relapsed SW837 xenograft tumors was significantly lower than in vehicle-treated, adagrasib-sensitive tumors (p = 0.0004) and was similar to levels in parental SW837 xenograft tumors at 4 to 48 h post-last dose of adagrasib (Fig. 6c). With our previous findings (Extended Data Fig. 5b,c), these data indicate that the modest rebound in overall MAPK pathway activity in parental SW837 xenograft tumors at 48 h post-last dose of adagrasib did not further increase during the acquisition of KRAS^G12C^i resistance in this model. Additionally, administration of adagrasib plus BI-3406 to adagrasib-resistant SW837 tumors further downregulated MAPK pathway activity when compared to levels in relapsed (*P* = 0.014) and parental SW837 tumors at 4 h post-last dose of adagrasib (*P* = 0.036) (Fig. 6c). Along with previous data (Fig. 6b), these findings further strengthen the potential of co-administering BI-3406 with adagrasib to induce tumor regression in adagrasib-resistant SW837 xenograft tumors.

**Extended Data Fig. 9:**
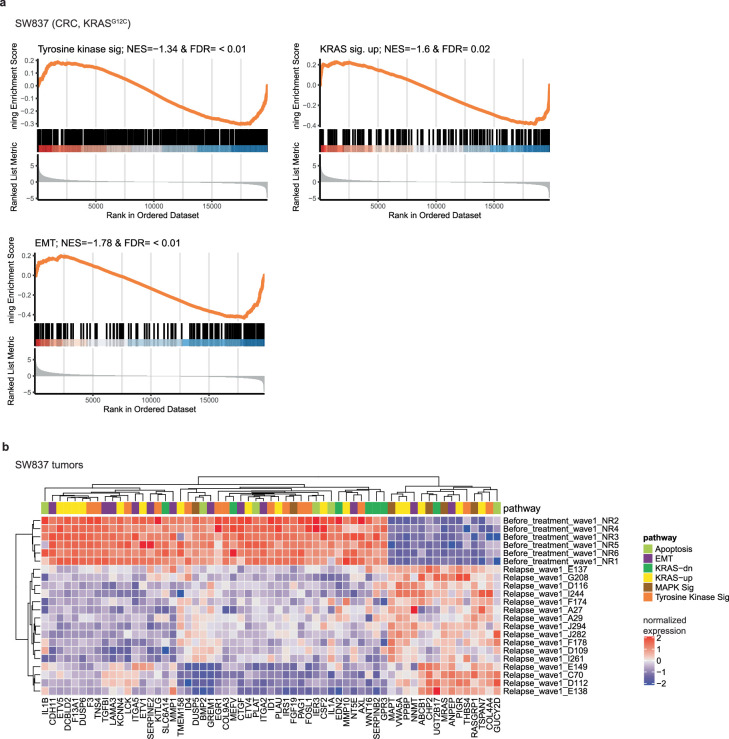
Molecular response of KRAS^G12C^i-resistant colorectal cancer (CRC) models to combination treatments. **a**, Gene set enrichment analysis (GSEA) plots show downregulated tyrosine kinase, EMT, and KRAS pathways between tumors collected from SW837 models prior to adagrasib treatment and during relapse after chronic adagrasib treatment. EMT = epithelial-mesenchymal transition. NES = normalized enrichment score; FDR = false discovery rate-adjusted q value. Upregulation (red) and downregulation (blue) are shown. N = 6 and 16 animals in before treatment and relapse groups, respectively. **b**, Heatmap showing expression z-score of genes belonging to apoptosis, epithelial-mesenchymal transition (EMT), or KRAS gene sets, as well as MAPK or tyrosine kinase signaling pathways from tumors collected from SW837 xenograft models prior to adagrasib treatment (Before Treatment, n = 6 sequenced tumors) and during relapse (Relapse, n = 16 sequenced tumors). Color legend: normalized expression. Source data

The transcriptomic changes that occur in SW837 tumors relapsing under adagrasib treatment were assessed. Identification of significantly enriched biological pathways was conducted using GSEA in a supervised manner on gene sets associated with KRAS^G12C^i (refs. ^9,11,36^). Our findings revealed that gene sets associated with tyrosine kinase signaling (*n* = 543), EMT (*n* = 204), as well as genes known to be regulated by active KRAS (*n* = 220), were downregulated in relapsed tumors, but no pattern of downregulation could be discerned (Extended Data Fig. 9a). Differential gene expression analysis revealed significant deregulation of 810 genes (242 and 568 genes down and upregulated, respectively) in tumors undergoing relapse (Fig. 6d, Supplementary Table 13). Further, *DUSP5* and *DUSP6*, two pharmacodynamic biomarkers of KRAS^G12C^i, were still downregulated in relapsed tumors (Fig. 6d and Extended Data Fig. 9b); this, together with the fact that no secondary *KRAS* mutations were detected, suggested that adagrasib was still able to bind and inhibit KRAS^G12C^.

**Extended Data Fig. 10:**
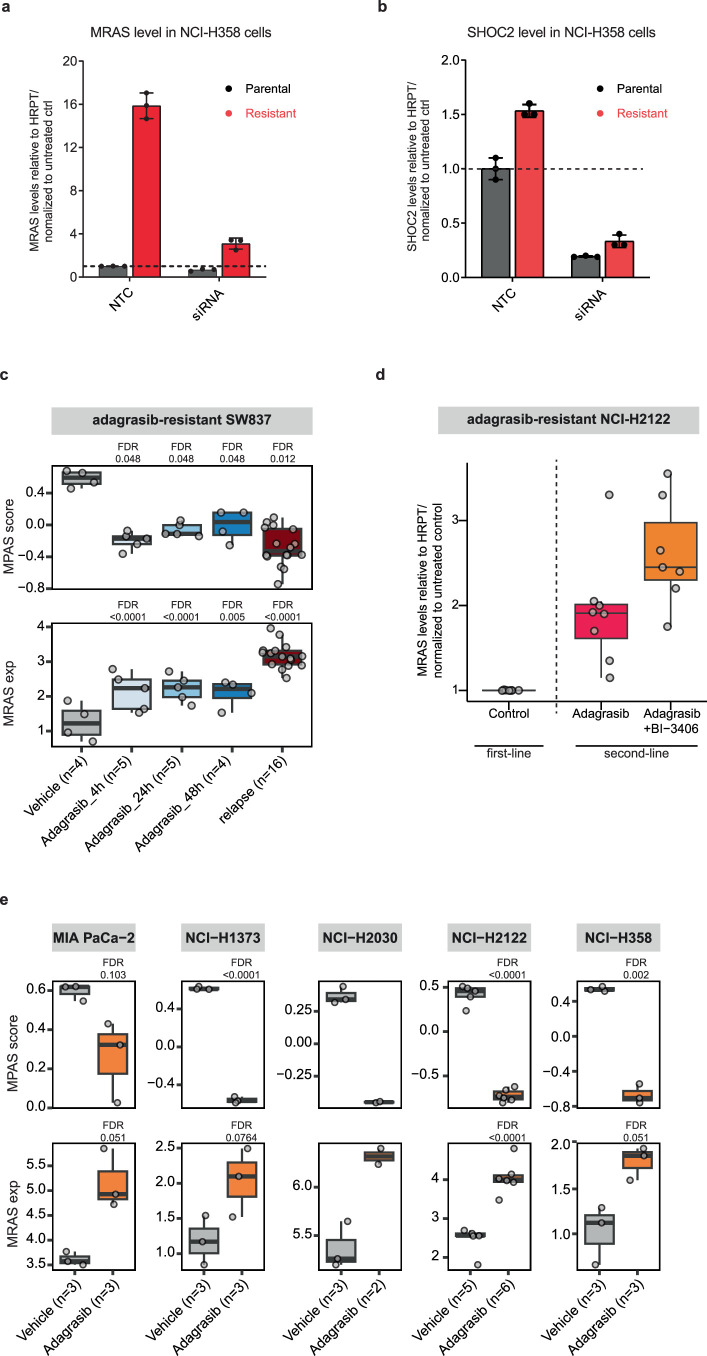
MRAS and SHOC2 regulation in adagrasib-sensitive and -resistant models. **a**, *MRAS* expression in parental and adagrasib-resistant NCI-H358 cells transfected with siRNA targeting MRAS (siRNA) or with a non-targeting control (NTC). Error bars indicate mean ± SD. The dotted line represents the baseline level in sensitive (parental NTC) cells. All groups: n = 3 qPCR from independent cell cultures. **b**, *SHOC2* expression parental and adagrasib-resistant (HD pool) NCI-H358 cells from (a) and transfected with siRNA targeting SHOC2 (siRNA) or non-targeting control (NTC). Dotted line represents the baseline level in sensitive (NTC parental) cells. n = 3 qPCR from independent cell cultures. Error bars indicate mean ± SD. **c**, MAPK activity (top) and *MRAS* expression (bottom) in SW837 xenograft tumors. SW837 models were treated with vehicle (n = 4 animals) or adagrasib (50 mg/kg) for 8 days (samples taken 4, 24, and 48 hours post-last dose, n = 4-5 animals/group) or adagrasib until relapse (n = 16 animals). Boxplots show low and upper quartiles and median line is indicated. Whiskers: 1.5 x interquartile range. Groups compared to vehicle using a two-sided Wilcoxon test; p values adjusted for multiple comparisons using Benjamini–Hochberg Method. FDR = false discovery rate-adjusted p value. Number of animals/group indicated on graph. **d**, *MRAS* expression in NCI-H2122 xenograft tumors treated with vehicle (Extended Data Fig. 8c) and in tumors following second-line treatment of adagrasib (100 mg/kg) alone or plus BI-3406 (50 mg/kg, bid) for 35 days (Extended Data Fig. 8d). Each boxplot shows lower and upper quartile; median line indicated. Whiskers: 1.5× interquartile range. Adagrasib group: N = 8 mice; other groups: N = 7 mice. **e**, MAPK activity (top) and *MRAS* expression (bottom) in indicated KRAS^G12C^-mutated xenograft models treated with vehicle or adagrasib (100 mg/kg); data collected 24 hours post-last dose. Boxplots show low and upper quartiles; median line, indicated. Whiskers: 1.5 x interquartile range. Groups compared to vehicle using a two-sided Wilcoxon test; p values adjusted for multiple comparisons using the Benjamini–Hochberg Method. FDR = false discovery rate-adjusted p value. Number of tumors sequenced/group indicated on graph. Source data

Notably, upregulated genes in relapsed SW837 xenograft tumor included *MRAS*, a homologue of the RAS family of GTPases^37^, and *RasGRP1*, a Ras GEF^38,39^, but not *NRAS* or *HRAS* (Fig. 6d, Extended Data Fig. 9b and Supplementary Table 13). SOS1 and RasGRP1 have been shown to facilitate the GTP-loading of MRAS^37,38^, which, together with SHOC2 and PP1C, forms a phosphatase complex that contributes to the increased activation of RAS-MAPK signaling through dephosphorylation of inhibitory sites on RAF kinases^40^. Consistent with these findings, transcriptomic analyses of adagrasib-resistant NCI-H358 cells revealed that, compared to parental cells, the resistant cells had significantly increased *MRAS* (16-fold) and *SHOC2* (2.5-fold) expression (Extended Data Fig. 10a,b), with upregulation of *MRAS* expression upon acquiring adagrasib resistance further confirmed in SW837 xenografts (Extended Data Fig. 10c). Increased *MRAS* expression was also observed in adagrasib-resistant NCI-H2122 models upon second-line adagrasib treatment, with a further increase upon second-line adagrasib plus BI-3406 treatment (Extended Data Fig. 10d). Notably, upregulation of *MRAS* expression was observed as early as 4 h after treatment initiation and was inversely correlated with MAPK signaling in SW837 xenografts (Extended Data Fig. 10c) and five other xenograft models (Extended Data Fig. 10e). Finally, partial re-sensitization of NCI-H358R cells to adagrasib alone or plus BI-3406 or TNO155 treatment was observed upon *SHOC2* knockdown (Fig. 6e), although this effect was not observed upon *MRAS* knockdown, likely due to the difficulty of achieving full *MRAS* knockdown in these models (Extended Data Fig. 10a).

In summary, acquired resistance to KRAS^G12C^i in our model systems is driven primarily by bypass pathway(s) that potentially involve MRAS and its complex partners, SHOC2 and PP1C (Fig. 6f). Notably, we show that co-treatment with SOS1i or SHP2i remains effective against KRAS^G12C^i-resistant models (Fig. 6b and Extended Data Fig. 8d), thus suggesting that these combination approaches may be effective in inhibiting MRAS-regulated resistance pathways (Fig. 6f).

## Discussion

Efforts to target mutant KRAS signaling in cancer have advanced tremendously, with multiple KRAS^G12C^-specific inhibitors in clinical or preclinical development^1,4,7,41–43^, but the rapid development of resistance limits progression-free survival^4,7^. Consequently, identifying rational KRAS^G12C^i combination strategies is urgently needed to improve responses to KRAS^G12C^-targeted therapy. Here, we show that co-administration of BI-3406 with adagrasib in preclinical KRAS^G12C^-mutant NSCLC and CRC models enhances the magnitude and duration of the antitumor response, and is comparable to those induced by clinically evaluated combinations, such as KRAS^G12C^i plus SHP2i or EGFRi (refs. ^16,20,21^). Further, we found that adagrasib plus BI-3406, TNO155 or cetuximab combination treatment induced strong antiproliferative effects in vitro and in vivo across a common set of KRAS^G12C^i-naive models, suggesting that the three combination approaches may provide benefit in similar populations of patients.

Co-administration of SOS1i or SHP2i both lead to the pharmacological blockade of nucleotide exchange^44^ and increased inactive RAS levels, thus potentiating the effect of KRAS^G12C^i. Moreover, co-targeting SOS1 or SHP2 may block proposed adaptive escape mechanisms, such as upregulation or activation of WT RAS isoforms^14,15^, which remain reliant on GEFs for activation. Consistent with previous studies reporting the combination benefit of KRAS^G12C^i plus SHP2i or RTKi (refs. ^1,13,16^), the enhanced antitumor effects induced by co-administration of BI-3406 with adagrasib are accompanied by enhanced suppression of RAS-GTP-loading as well as reduced downstream MAPK signaling and proliferation. Our data imply that KRAS^G12C^i plus SOS1i combination treatment may lead to a more profound inhibition of KRAS downstream signaling compared to KRAS^G12C^i monotherapy, thereby eliciting stronger and more durable responses in the clinic.

Emerging preclinical and clinical evidence have identified multiple mechanisms contributing to resistance to KRAS^G12C^i (refs. ^1,7,10,13–15,45^), such as RTK activation^16^, *KRAS*^*G12C*^ amplification^46^, activation of alternative signaling pathways including PI3K, mTOR, YAP/TAZ^4,7^, acquisition of secondary *KRAS* mutations that preclude compound binding (for example R68S, Y96C/D and H95D/Q/R)^3,9,11^, conversion of cysteine to another amino acid in position 12, and additional activating *KRAS* mutations (G13D and Q61H)^11,34^. Many of the aforementioned mutations were detected only at low frequencies and only in approximately half of patients relapsing on KRAS^G12C^i (ref. ^11^). In the remaining patients, no genomic mechanisms of resistance could be identified^13,16,17,47^, suggesting that non-genetic alterations could be involved. Overall, none of the aforementioned genetic mechanisms was empirically shown to be the primary drivers of resistance in our models.

Transcriptional changes, primarily through the upregulation of *MRAS* and *RasGRP1*, were likely the probable contributors to acquired resistance to KRAS^G12C^i in the majority of our models, with the exceptions of F3008 and B8032 models, the outgrowth of which may have been driven by the aforementioned genetic mechanisms as upregulated MRAS expression was not observed in adagrasib-treated groups. While aberrant MRAS activity in cancer is rare^37^, activating mutations in *MRAS* (Q71R) have been reported at low allelic frequency in KRAS^G12C^i-resistant NSCLC cell lines^11^. Further, GTP-loading of MRAS has been shown to be facilitated by RasGRP1 (ref. ^39^), which was upregulated in adagrasib-resistant SW837 tumors. Sotorasib treatment has recently been shown to result in increased *MRAS* expression and the switch of membranous Scrib-SHOC2 to MRAS-SHOC2, thereby activating MAPK signaling^14^. Of note, co-administration of BI-3406 or SHP099 inhibited sotorasib or adagrasib-induced upregulation of active MRAS and further suppressed cell proliferation in vitro^14^. While no upregulation of active MRAS in SW837 cells was observed in vitro, *MRAS* expression rapidly increased in SW837 xenografts treated with adagrasib and was even higher in SW837 and NCI-H2122 tumors that relapsed on adagrasib. Moreover, co-treatment with SOS1i led to a further increase in *MRAS* expression in NCI-H2122 tumors, likely due to the enhanced downregulation of KRAS activity by this combination. Given that SOS1i and SHP2i very potently inhibit MRAS activation, this increase in *MRAS* expression did not seem sufficient to dampen the enhanced antitumor effect of adagrasib in combination with either inhibitor. Finally, knockdown of SHOC2, a complex partner of MRAS, partially re-sensitized adagrasib-resistant NCI-H358 cells to adagrasib and even more so to adagrasib plus SOS1i or SHP2i combination treatments. Together, our findings support MRAS activation to be a potential mechanism of resistance to KRAS^G12C^i.

Given that we did not observe a rebound of MAPK signaling in adagrasib-resistant SW837 xenografts or NCI-H358R cells, it is likely that MRAS contributes to adagrasib resistance via a MAPK-independent mechanism. This is consistent with previous findings that MRAS, alone, is a poor activator of MAPK signaling^38,48,49^. While MRAS shares approximately 50% sequence identity with other RAS isoforms and can interact with some of the common RAS effectors and regulators, such as RAF, PI3K, RalGDS and SOS^38,50,51^, it also uniquely associates with SHOC2 and PPI1C^40^ as well as certain RapGEFs^52,53^. This suggests that MRAS may regulate other signaling pathways involving Rho, Ral or Rap GTPases^38,53,54^, contributing to tumor cell migration adhesion, endocytosis or invasion. Indeed, in SW837 xenografts, we also observed an upregulation of several Rho GEFs, including *TAGAP*, *ARHGEF40*, *FGD2* and *TIAM1*, supporting Rho signaling being involved in resistance to KRAS^G12C^i; however, additional functional studies, are needed to clarify the role of elevated MRAS and RasGRP1 in mediating resistance to KRAS^G12C^i.

In addition to MRAS activation in NCI-H2122 xenografts, adagrasib treatment led to the upregulation of genes related to oxidative phosphorylation, genes upregulated by reactive oxygen species and Myc target genes, which may contribute to survival during KRAS inhibition. Co-treatment with SOS1i or SHP2i seemed to counteract this effect, which may also be the result of MRAS modulation, as MRAS has previously been shown to activate JNK^54^, a known regulator of MYC. However, the exact molecular mechanism remains to be elucidated.

Notably, in an adagrasib-resistant CRC model, we show that combination of adagrasib plus BI-3406 or TNO155 was more efficacious than adagrasib plus cetuximab. This suggests that KRAS^G12C^i-resistance in a subset of tumors may be driven by non-EGFR-dependent mechanisms, potentially other RTKs or alternative pathways. Indeed, the ability of SOS1 and SHP2 to regulate RAS signaling downstream of multiple RTKs may explain the enhanced antitumor response observed^20,55^. Of note, while our findings suggest that SHP2i and SOS1i may induce the same efficacy, inhibiting SOS1 avoids some of the pleiotropic effects of SHP2 inhibitors^56,57^, potentially resulting in better clinical tolerability.

Clinical studies have also shown that KRAS-mutant tumors with certain co-mutations, such as *KEAP1* or *STK11*, are less responsive to certain therapeutic approaches, including anti-PD1 (ref. ^58^) or KRAS^G12C^i (ref. ^59^). In the 14 KRAS^G12C^ models tested, four carried *KEAP1* and three carried *STK11* co-mutations. No correlation between the mutational status of these genes and sensitivity to any of the tested combination treatments was observed. In isogenic murine lung tumor cell lines, however, loss of either *Lkb1/Stk11* or *Keap1* resulted in reduced sensitivity to adagrasib. Further, co-administration with a SHP2i or SOS1i re-sensitized *Keap1*, but not *Lkb1*, knockout cells to KRAS^G12C^i. The mechanisms of differential resistance of *Lkb1* mutations, in contrast to *Keap1* loss to combination therapy, is under investigation. Our transcriptomic analyses demonstrate that cell lines weakly responsive to the combination treatments exhibited higher expression of genes in cancer hallmark pathways, such as EMT, PI3K/AKT/mTOR signaling and TGFβ signaling. Further investigation is required to determine whether these characteristics could be used as clinical biomarkers of response.

Finally, a new class of KRAS allele-specific inhibitors, targeting the active GTP-bound state of KRAS (KRAS^G12C^(ON) inhibitors), has recently entered the clinic (NCT05462717)^56,57^. Early preclinical data indicate that co-treatment of KRAS^G12C^(ON) inhibitors with SHP2i prolongs the durability of response^60^. Given the similar mode of action of SOS1i and SHP2i, we hypothesize that combination with a SOS1i may delay development of acquired resistance by deepening the inhibition of the KRAS pathway^13,16,17,47^; however, separate studies are needed to confirm this.

Overall, our findings underscore the potential of co-administering a SOS1i with allele-specific KRAS inhibitors to improve and prolong clinical responses in patients with cancer driven by mutant *KRAS*.

## Methods

### Cell lines

Cell lines were purchased from American Type Culture Collection (ATCC). The original LKR13K (Kras^G12D^) cells derived from Kras^LA1-G12D^ mice^26^, LKR13KL (Kras^G12D^; Lkb1^−/−^) and LKR13KK (Kras^G12D^; Keap1^−/−^) cells were a gift from J. Heymach’s laboratory at MD Anderson Cancer Center. MIA PaCa-2 and SW837 cells were cultured in high-glucose DMEM (Sigma, D6429) with 10% FBS (Hyclone, Thermo Fisher Scientific, SH30084), NCI-H2122, NCI-H358 and LKR13 cells were cultured in RPMI (Gibco, 3A10491-01) with 10% FBS at 37 °C and 5% CO_2_ in a humidified incubator. For high-throughput screens, SW837 and NCI-H358 cells were cultured in high-glucose DMEM (Sigma, D6429) with 10% FBS in the presence of pen–strep (Gibco, 15140-106). CRISPR-Cas9-engineered human tumor cell lines were generated at Horizon Discovery.

### Combination studies: cell culture

NCI-H2122 (human NSCLC cells, ATCC CRL-5985) cells were grown as described above. After expansion and passaging cells twice a week with a 1:2 to 1:4 dilution, we seeded 800 cells per well in 60 µl in 384-well plates (Greiner, 781182) for the proliferation assay. After 24 h, compounds were added with an ultrasonic dispersion system (Echo, Labcyte System) and incubated for another 72 h. Cells were then stained with CellTiter-Glo Reagent according to the manufacturer’s protocol (Promega, G924C) and incubated 15 min under shaking. Plates were then read with a plate reader (EnVision, PerkinElmer HTS Multilabel).

SW837 (human colon cells, ATCC CCL-235) cells were grown as described above. After expansion and passaging the cells twice a week with a 1:10 to 1:12 dilution, we seeded 500 cells per well in 60 µl in 384-well plates (Greiner, 781182) for the proliferation assay. After 24 h, compounds were added with an ultrasonic dispersion system (Echo, Labcyte System) and incubated for another 144 h. Cells were then stained with CellTiter-Glo Reagent according to the manufacturer’s protocol (Promega, G924C) and incubated 15 min under shaking. Plates were then read with a plate reader (EnVision, PerkinElmer HTS Multilabel).

NCI-H358 (human NSCLC cells, ATCC CRL-5807) cells were grown as described above. After expansion and passaging the cells twice a week with a 1:10 to 1:12 dilution, we seeded 500 cells per well in 60 µl in 384-well plates (Greiner, 781182) for the proliferation assay. After 24 h, compounds were added with an ultrasonic dispersion system (Echo, Labcyte System) and incubated for another 72 h. Cells were then stained with CellTiter-Glo Reagent according to the manufacturer’s protocol (Promega, G924C) and incubated 15 min under shaking. Plates were then read with a plate reader (EnVision, PerkinElmer HTS Multilabel).

### CRISPR engineering of KRAS^G12C^ syngeneic mouse lung cancer cell lines

To convert LKR13K, LKR13KK and LKR13KL cell lines from Kras^G12D^ to Kras^G12C^ by homologous-directed repair, 50,000 cells were plated in a six-well plate and endogenous Kras^G12D^ was knocked out using the snRNP complex by co-transfecting 13.8 µg of Cas9 nuclease (IDT) with 2.8 µg of G12D sgRNA (5′–GTGGTTGGAGCTGATGGCGT–3′) and 1 µM of G12C ssODN (5′–AGTTGTATTTTATTATTTTTATTGTAAGGCCTGCTGAAAATGACTGAGTATAAGCTTGTGGTGGTTGGAGCTTGTGGTGTAGGCAAGAGCGCCTTGACGATACAGCTAATTCAGAATCACTTTGTGGATGAGTATGACCC–3′) using Lipofectamine CRISPRMax reagent (Thermo). Forty-eight hours after transfection, cells were cultured in the presence of 1 µM MRTX1133 (Kras^G12D^ inhibitor) to select for positively converted cells.

### Growth curve fitting and combination scoring

Curves were fitted using standard nonlinear regression with a four-parameter fit after normalization to percentage of maximum signal (the negative dimethylsulfoxide (DMSO) controls) for each treatment type. The combination cScore quantifies the deviation of the observed effect of two drugs combined when compared to the expected effect based on the observed monotherapies at the same concentration. For this, the measured cell viability is subtracted from the expected cell viability using the Bliss Independence Model^61^, creating a ‘gap’ matrix with the two monotherapy concentrations on the *x* and *y* axis, respectively and the unit (%PoC). The cScore is then defined as the average gap value of a 3 × 3 field matrix around the IC_50_ of both compounds or, if one or both compounds have no measurable IC_50_, the scores at the highest two concentrations of the Gap table. If the IC_50_ is close to the C_max_ (maximum concentration of compound used) or C_min_(minimum concentration of compound used), the field used for averaging may be 2 × 3 or 2 × 2, but never <4. This approach focuses on drug combination effects around the IC_50_ values of the respective monotherapies. Positive cScores thus express the average increased potency of the combination over the monotherapies around the IC_50_ in % cell viability PoC.

### Western blot

SW837 or NCI-H2122 cells were seeded in six-well plates (Corning, 3506) at a density of 2.5 × 10^6^ or 1 × 10^6^ cells per well in complete culture medium. After overnight incubation at 37 °C and 5% CO_2_ in a humidified incubator, cells were treated with indicated concentrations of MRTX849, BI-3406, TNO155 or SHP099 alone or in combination; control cells were treated with 0.1% DMSO (Sigma-Aldrich, 41648). After 6 or 24 h of treatment, cells were washed with ice-cold PBS, collected and lysed with MSD lysis buffer (Mesoscale Diagnostics, R60TX-2) containing Tris, pH 7.5, 150 mM NaCl, 1 mM EDTA, 1 mM EGTA, 1% Triton X-100, 10 mM NaF, completed with protease and phosphatase inhibitors (Thermo Fisher Scientific, 78440). Protein concentrations were determined via Bradford assay according to manufacturer´s instruction (Bradford Dye, Bio-Rad, 5000205). Unless otherwise stated, 20 µg of total protein was separated on a 4–12% polyacrylamide gel (Bio-Rad, 3450124) in MOPS Running Buffer (Bio-Rad, 1610788) and blotted on a PVDF membrane (Bio-Rad, 1704157) with the Bio-Rad Trans-Blot Turbo Instrument. For separation of KRAS shift, total protein was separated on a 10% Tris-HCL polyacrylamide gel (Bio-Rad, 3450021) in Tris Glycine Running Buffer (Bio-Rad, 1610732). Membranes were blocked for 1 h in 4% skim milk (Millipore, 70166) in 1× TBS (Bio-Rad, 1706435)/0.1% Tween 20 (Bio-Rad, 161-0781) at room temperature and then probed overnight at 4 °C with primary antibodies against KRAS (LSBio, LS-C175665; 1:500 dilution), p-ERK Thr202/Tyr204 (Cell Signaling, 4376; 1:500 dilution), ERK (Cell Signaling, 9102; 1:1,000–1:2,000 dilution), phospho-S6 Ribosomal Protein (Ser235/236) (Cell Signaling, 2211; 1:500–1:1,000 dilution), DUSP6 (Abcam, ab76310; 1:1,000 dilution), cleaved PARP (Asp214) (Cell Signaling, 9541; 1:1,000 dilution), cyclin D1 (Biosite, ARB-Q4OL25-0,5; 1:100 dilution); p27 (BD, 610241; 1:1,000 dilution) and β-actin (Abcam, ab8226; 1:10,000 dilution). The phosopho-S6 ribosomal protein dilution was prepared in 5% BSA in 1× TBS/0.1% Tween 20, and all other antibody dilutions were prepared in 4% skim milk in 1× TBS/0.1% Tween 20. Cytiva Rainbow Molecular Weight Markers were used (Fisher Scientific, 45-001-591). After washing, membranes were incubated with the following secondary antibodies diluted in respective incubation buffers: goat anti-rabbit IgG, HRP conjugated (Dako, P0447; 1:1,000 dilution) and goat anti-mouse IgG, HRP conjugated (Dako, P0448; 1:1,000 dilution). Proteins were visualized using ECL Western Blotting detection reagent (Amersham, RPN2106) according to the manufacturer’s instructions.

For western blotting following the RAS pulldown assay the samples were eluted from the resin already in the provided 2× SDS sample buffer provided in the pulldown kit described below. All samples were incubated for 5 min at 95 °C and loaded onto 12% Criterion XT Bis-Tris Protein Gels (Bio-Rad, 3450118). Gels were transferred to a 0.2-µm PVDF membrane (Bio-Rad, 1704157) with the Trans-Blot Turbo Transfer System using the standard program for 30 min at 25 V. The following primary antibodies were used for immunoblotting: KRAS (1:1,000 dilution; LSBio, LS-C175665), HRAS (1:500 dilution; Proteintech, 18295-1-AP), MRAS (1:200 dilution; Abcam, ab176570), NRAS (1:1,000 dilution; Abcam, ab167136) and GAPDH (1:1,000 dilution; Cell Signaling, 2118).

### GTP-KRAS ELISA

GTP loaded KRAS was quantified using the KRAS In-well Lysis ELISA kit from Active Motif (cat. no. 52100) according to the manufacturer’s recommendations. In brief, 300,000 cells (MIA PaCa-2) or 600,000 cells NCI-H2122, SW837, NCI-H358) were seeded in a six-well plate and incubated for 48 h before addition of the drug in duplicates. The plates were incubated at 37 °C, 5% CO_2_ for specified amount of time. Cells were washed with PBS and lysed in 150 µl per well using Complete Lysis/Binding Buffer (ELISA kit from Active Motif). Wells were sealed and incubated for 15 min at 4 °C on a shaker (100–200 rpm). Supernatant of the lysates was transferred to a new plate, coated with GST-CRAF RBD provided by the manufacturer. Further incubation (primary and secondary antibody addition), washing and detection were performed according to instructions. Chemiluminescence was measured using the PerkinElmer EnSpire Multimode Reader.

### Active RAS pulldown assays

NCI-H358, NCI-H2122 and SW837 cells were seeded into 15-cm dishes at a cell number selected based on proliferation rate and duration of drug treatment, to reach a confluency of 80–90% at the day of the lysate preparation. On the following day, the cells were treated with adagrasib (150 nM), SHP2i TNO155 (1,000 nM), BI SOS1i BI-3406 (1,200 nM) or a combination of adagrasib with BI-3406 or adagrasib with TNO155. Cells treated with DMSO (0.027%) served as vehicle control. Cells were treated for either 2 h or 48 h and subsequently collected for the assay using the Active Ras Pull-Down and Detection kit (Thermo Scientific, 16117). Pull-downs were performed according to manufacturer’s protocol with the following adaptation: Lysis/Binding/Wash buffer was supplemented with Halt Protease and Phosphatase Inhibitor Cocktail (100×) (Thermo Scientific, 78441). Given that NCI-H2122 cells are semi-adherent, the cells in the supernatant were collected, pelleted by centrifugation and added to the cells scraped off from the dishes. Then, 500 µg total protein was applied as input material for the pulldown per spin cup. Then, β-mercaptoethanol was added to complete the provided 2× SDS sample buffer. Pulldowns were performed in duplicates and pooled after elution from the resin to ensure sufficient material for SDS–PAGE and immunoblotting.

### IncuCyte kinetic cell confluence proliferation assay

SW837 and NCI-H2122 cells were seeded in 96-well plates (Corning, 3598) at a density of 4,000 cells per well in complete culture medium. Following overnight incubation at 37 °C and 5% CO_2_ in a humidified incubator, cells were treated with indicated concentrations of MRTX849, BI-3406, TNO155 or SHP099 as monotherapy or in combination. Control cells were treated with 0.1% DMSO. Immediately before treatment, cells were stained with IncuCyte caspase‐3/7 Green Apoptosis Assay Reagent (Essen BioScience, 4440) for apoptosis detection according to the manufacturer’s instruction. Cell growth was monitored using the IncuCyte S3 live cell imaging system (Essen BioScience). Using the ×10 objective, two regions of view were collected per well every 4 h for at least 7 days, with an extended collection period in cases of slow cell growth. Phase contrast and green channel (ex, 440/80 nm; em, 504/44 nm) were collected for each experiment. Data were analyzed using IncuCyte 2019B software. Values from both regions of each well were averaged and confluence was calculated as the percentage of the image area that was occupied by objects (phase area confluence). Apoptotic events were calculated as the percentage of the image area that was occupied by green objects.

### siRNA transfection and cell viability

For knockdown experiments, NCI-H358-resistant cells were transfected with ON-TARGETplus SMARTpool siRNA duplexes (Dharmacon) targeting SHOC2 (L-019524-01) consisting of J-019524-09 GAAGAGAAUUCAAUGCGUU, J-019524-10 CGUCUUGGUCUGAGAUAUA, J-019524-11 UCGUAUAACUACUGUGGAA and J-019524-12 GAGGUAGUAUAGUUAGAUA, MRAS siRNA pool (L-008586-00) consisting of J-008586-05 ACACAAUAUUCCGUACAUA, J-008586-06 CAACAAGGUCGAUUUGAUG, J-008586-07 GUAAUUAGGCAACAGAUUC and J-008586-08 UCAAAGACAGGGAGUCAUU or a non-targeting control using Lipofectamine RNAiMAX reagent according to the manufacturer’s instructions (Invitrogen). Cell viability assays were then performed using 10 nM siRNA in 96-well plates in a total volume of 100 µl per well. Adagrasib (300 nM), SOS1i (1 µM) and SHP2i (1 µM) were added 4 h after siRNA transfection. Viability was determined using CellTiter-Glo (Promega) 96 h post-transfection. Then, 30 µl CellTiter-Glo solution was added directly to the cell medium, mixed and incubated for 10 min before determination of the luminescence signal. The effectiveness of the knockdown was confirmed by qPCR. Treated cells were lysed and prepared for qPCR using the FastLane Cell RT–PCR_QuantiTect Multiplex RT–PCR kit (QIAGEN, 216513) according to the manufacturer’s protocol. The final reaction was prepared with 3 µl RNA lysate as a multiplex reaction in technical triplicates using MRAS TaqMan Assay probe Hs01031059_m1 (FAM) together with the endogenous control HPRT Hs02800695_m1 (VIC) from Thermo Fisher Scientific.

### Whole-exome sequencing capillary immunoassay

Proteins were extracted in RIPA lysis buffer (Sigma-Aldrich, R0278) supplemented 1:100 with Halt Protease and Phosphatase Inhibitor Cocktail (Thermo Scientific, 1861282). Then, 0.5 µg µl^−1^ protein was used for the final reaction. Proteins were detected using the capillary immunodetection kit from Protein Simple (12–230 kDa Separation 8 × 25 Capillary Cartridges, SM-W004-1). Samples were diluted with the provided reagents of the EZ Standard Pack 1 (12–230 kDa, PS-ST01EZ-8) according to the manufacturer’s instructions. Antibodies were used to detect MRAS levels (anti-MRas antibody (EPR12457), rabbit ab176570, Abcam, 1:50 dilution). Vimentin (Vimentin (D21H3) XP, rabbit, 1:50 dilution, 5741, Cell Signaling) and E-cadherin (ab231303, mouse, 1:100 dilution, Abcam) were used to detect EMT and anti-actinin was used for normalization (1:200 dilution, rabbit, 3134, Cell Signaling). Secondary antibodies against mouse and rabbit were also obtained from Protein Simple (Anti-Mouse Detection Module, DM-002 and Anti-Rabbit Detection Module, DM-001).

### Oncogenic KRAS variant generation and viability

Ba/F3 cells (DSMZ, cat. no. ACC300) were grown in RPMI-1640 medium supplemented with 10% FCS at 37 °C in 5% CO_2_ atmosphere in the presence of 10 ng ml^−1^ interleukin-3 (R&D).

Ba/F3 cells were transduced with retroviruses encoding pMSCV_KRAS_G12C_single site variant Library (ssvL) cloned at TWIST Biosciences and harboring a pool of each possible variant at any AA position of the KRAS gene (3,756 variants), a puromycin resistance gene and green fluorescent protein (GFP). Platinum-E cells (Cell Biolabs) were used for retrovirus packaging. Retrovirus and 4 μg ml^−1^ polybrene were added to Ba/F3 cells for spinfection. Infection efficiency was confirmed by measuring GFP-positive cells using a cell analyzer. The infection rate was kept low (~6%) to promote the single infection of one cell. Cells with a library representation of >1,000× were further cultivated in the presence of puromycin (1 μg ml^−1^) to select for transduced cells. Following selection, interleukin-3 was withdrawn from transgenic Ba/F3 cells expressing the oncogenic KRAS variants (KRAS ssvL Ba/F3) to make cells dependent on transgene activity. Initial quality check of the library was then performed by RNA sequencing to ensure proper library coverage.

For colony growth assays, KRAS ssvL BaF/3 cells were seeded into 96-well plates at 250 cells per 100 μl in growth medium, providing only a subset of the mutation spectrum per well, and allowing slower-growing variants to arise by preventing stronger drivers to overgrow the resistant population. We used 5 × 96 wells for each condition. The 481 wells provide a ~30-fold coverage of each variant for each condition. Compounds were added alone or in combination with two different doses of SOS1i. Treated cells were incubated for 14 days at 37 °C with 5% CO_2_ with an addition of 100 µl medium/cpd mixture after 7 days. AlamarBlue Cell Viability assay (Thermo Fisher) was performed, and after 6 h, fluorescence was measured by using the multilabel Plate Reader VICTOR X4. The raw data were imported into Microsoft Excel and the signal to background ratio was calculated. Ratios >1.5 were counted as colony growth and then visualized in Prism, GraphPad.

### Cell line-derived efficacy studies and biomarker studies in mice

We used 7–10-week-old female BomTac:NMRI-*Foxn1*^nu^ (SW837 and NCI-H2122) or 7-week-old female CB-17/Icr-Prkdc^scid^/Rj (NCI-H358) mice for all xenograft studies. For biomarker and efficacy experiments using SW837 or NCI-H358 tumor-bearing mice, female mice were engrafted subcutaneously with 5 million cells suspended in Matrigel (Corning, 356231) diluted in 1× PBS with 5% FCS. For biomarker and efficacy experiments using NCI-H2122 tumor-bearing mice, female mice were engrafted subcutaneously with 5 million cells suspended in in 1× PBS with 5% FCS. Tumors were randomized by tumor size in groups using the automated data storage system Sepia. Mice were treated once at time point 0 h (qd, once daily) and in addition 6 h later in case of twice daily treatment (bid, twice daily). Tumor size was measured by an electronic caliper and body weight was monitored daily. The analysis follows largely the procedures described previously^62,63^. BI-3406, adagrasib and TNO155 were dissolved in 0.5% Natrosol plus 0.5% DMSO. BI-3406 was administered at 50 mg kg^−1^ orally twice a day, adagrasib at either 50 or 100 mg kg^−1^ orally once per day, and TNO155 at 10 mg/kg, bid. Trametinib was dissolved in 0.5% DMSO and 0.5% Natrosol and dosed twice daily with 0.1 mg kg^−1^. Cetuximab was dissolved in 0.9% NaCl and dosed at 20 mg kg^−1^ i.p. twice a week. The control group was treated with 0.5% of Natrosol orally and 0.9% NaCl i.p. in the same frequency as in the treatment groups in the respective experiments (twice daily (orally), twice weekly (i.p.)). All compounds were administered either intragastrically by gavage (10 ml kg^−1^) or i.p. (cetuximab). Mice included in the biomarker studies were treated for 7 continuous days. Tumors were explanted at 4 h, 24 h or 48 h after the last dose and were either embedded in paraffin or fresh frozen for further analysis.

For investigating the efficacy of treatments on SW837 xenograft models with acquired adagrasib resistance, animals bearing established SW837 tumors were treated long-term with 50 mg kg^−1^ adagrasib for 5 days on and 2 days off per week. Tumor size was measured by an electronic caliper and body weight was monitored daily. Most tumors first underwent regression, with outgrowth occurring after several weeks of treatment. Outgrowing tumors that reached an increase of tumor size of at least 100 mm³ compared to the smallest size of the respective tumor then were randomized on day 63 and 84 for inclusion in treatment groups (*n* = 9 mice) for a second treatment. Results of the efficacy experiments starting on day 63 and day 84 were combined for analysis as no difference was observed in the outcome.

For investigating the efficacy of BI-3406 and adagrasib combination rechallenge after initial adagrasib monotherapy, animals bearing established NCI-H2122 tumors were first treated daily with 100 mg kg^−1^ adagrasib or with vehicle control. Tumor size was measured by an electronic caliper and body weight was monitored daily. As the majority of adagrasib-treated tumors did not go into regression, we re-randomized tumors at an average size of 300 mm^3^ after 15 days of treatment. Tumors were randomly reassigned using the automated software Sepia (as described) to either continue adagrasib monotherapy (100 mg kg^−1^ daily) or receive the combination of adagrasib (100 mg kg^−1^ daily) with BI-3406 (50 mg kg^−1^ twice daily) for a further 35 days.

All animal studies performed at Boehringer Ingelheim were approved by the internal ethics committee and the local Austrian governmental committee, with maximal tumor size/burden being greater or equal to 1,500 mm^3^. Maximal tumor size was not exceeded in any study. Mice used in Boehringer Ingelheim studies are group housed within environmentally controlled conditions with a 12-h light–dark cycle at 21 ± 1.5 °C, 55 ± 10% humidity and received food and water ad libitum. Sample sizes were determined by performing power analysis.

### PDX studies

PDX model characterization and profiling have been described previously^23^. The F3008 PDX study was performed in female NSG (NOD.Cg-Prkdc^scid^ Il2rg^tm1Wjl^/SzJ) mice (The Jackson Laboratory, cat. no. 005557) and the B8032 PDX study was performed in female athymic nude (NU(NCr)-Foxn1^nu^; strain 490) mice (Charles River Laboratories, cat. no. 24106219). For each model, tumor fragments (4 × 4 × 4 mm^3^) were implanted on the right hind flanks of the mouse host and allowed to grow to an average volume of 100–250 mm^3^ as monitored by caliper measurements. All orally administered compounds were dosed on a schedule of 5 days on and 2 days off. At enrollment, animals were randomized and treated with vehicle (0.5% Natrosol) orally twice a day (6 h apart), BI-3406 at 50 mg kg^−1^ orally twice a day (6 h apart), cetuximab at 15 mg kg^−1^ i.p. twice a week, SHP099 at 25 mg kg^−1^ orally once per day, adagrasib at 100 mg kg^−1^ orally once per day, or adagrasib (orally once per day) plus BI-3406 at 50 mg kg^−1^ orally twice a day (6 h apart) or cetuximab at 15 mg kg^−1^ i.p. twice a week. Mice were 11 weeks old and treatment group sizes included at least 5–8 mice per group. All animals received LabDiet 5053 chow ad libitum. Adagrasib was purchased from MedChemExpress, cetuximab was purchased from MD Anderson Cancer Center pharmacy and BI-3406 was synthesized at Boehringer Ingelheim. During the PDX studies, tumor growth was monitored twice a week with calipers and the TV was calculated as TV = (*D* × *d*^2^/2), where *D* is the largest and *d* is the smallest superficial visible diameter of the tumor mass. All measurements were documented as mm^3^. Body weights were measured twice weekly and used to adjust dosing volume and monitor animal health. All procedures for PDX studies were reviewed and approved by the Institutional Animal Care and Use Committee (00000884-RN04) at MD Anderson Cancer Center, with maximal tumor size/burden being greater or equal to 2,000 mm^3^. Maximal tumor size was not exceeded in any study. Mice used in PDX studies were group housed within environmentally controlled conditions with 12-h light–dark cycle at 21–23 °C, 40–60% humidity and received LabDiet 5053 chow and sterile water ad libitum. Sample sizes were determined based on a previous, similar study^23^.

### Pharmacodynamic biomarker analysis

For the pharmacodynamic biomarker studies using F3008 CRC PDX models, tumor fragments (4 × 4 × 4 mm^3^) were implanted on the right hind flanks of NSG female mice (Jackson Laboratory) and allowed to grow to an average volume of 250–350 mm^3^ as monitored by caliper measurements. At enrollment, animals were randomized and treated for 5 days with vehicle (0.5% Natrosol) orally twice a day (6 h apart), adagrasib at 100 mg kg^−1^ orally once per day, or adagrasib plus BI-3406 at 50 mg kg^−1^ orally twice a day (6 h apart) or cetuximab at 15 mg kg^−1^ i.p. on day 1 and day 4. Tumors were collected 4 h after the last dose on the fifth day of treatment and fixed in 10% neutral buffered formalin overnight and then processed and embedded in paraffin. Formalin-fixed and paraffin-embedded (FFPE) blocks were sectioned into 3-μm thick sections, deparaffinized, then rehydrated by serial passage through xylene and graded alcohol. Sections were subjected to an initial heat-induced epitope retrieval in citrate buffer, pH 6, at 95 °C for 15 min. Anti-phospho-p44/42 MAPK (ERK1/2) (Thr202/Tyr204) (1:2,000 dilution, Cell Signaling Technology, 4370) was developed using Opal tyramide signal amplification followed by direct immunofluorescence of HLA conjugated to Alexa 647 (1:250 dilution, Abcam, 199837). An RNAscope in situ hybridization assay was performed following the manufacturer’s protocol (Advanced Cell Diagnostics) using DUSP6 (cat. no. 405361), EGR1 (cat. no. 457671-C2) and POLR2A (cat. no. 310451-C4) probes. Appropriate positive and negative controls were included with the study sections. Digital images of whole-tissue sections were acquired using Vectra Polaris Automated Quantitative Pathology Imaging System (Akoya Biosciences) and representative regions were selected for each whole slide and processed using inForm Software v.2.4 (Akoya Biosciences). Processed images were then analyzed using HALO Software v.3.2 (Indica Labs).

For pharmacodynamic biomarker studies using NCI-H2122 and SW837 models, animals were first randomized and treated for 5 days with the dosing schedules described above. Tumors were collected at 4, 24 and 48 h after last dose of treatment. Tumor samples were FFPE and processed as described above. In brief, FFPE blocks were sectioned into 3-μm thick sections, deparaffinized and rehydrated by serial passage thorough xylene and graded alcohol. Sections were then subjected to heat-induced antigen retrieval and stained for anti-phospho-p44/42 MAPK (ERK1/2) (Thr202/Tyr204) and anti-KI-67 (CST, 9027, 1:400 dilution in PBS/2% BSA) on bond RX (LEICA). Appropriate negative and positive controls and isotype controls were included. Digital images of whole tissue sections were acquired with NanoZoomer scanner (Hamamatsu) and processed images were then analyzed using HALO Software v.3.2 (Indica Labs). Results were reviewed by a pathologist.

### Measurement of plasma concentrations

Compound concentrations in plasma aliquots were measured by quantitative HPLC–MS/MS using an internal standard. Calibration and quality control samples were prepared using blank plasma from untreated animals. Samples were precipitated with acetonitrile and injected into a HPLC system (Agilent 1200). Separation was performed by gradients of 5 mmol l^−1^ ammonium acetate pH 4.0 and acetonitrile with 0.1% formic acid on a 2.1 × 50 mm Xbridge BEH C18 reversed-phase column with 2.5 µm particles (Waters). The HPLC was interfaced by ESI operated in positive ionization mode to a triple quadrupole mass spectrometer (5000 or 6500+ Triple Quad System, SCIEX) operated in multiple reaction monitoring mode. Chromatograms were analyzed with Analyst (SCIEX) and pharmacokinetic parameters were calculated by non-compartmental analysis using BI proprietary software.

### DNA isolation for WGS

At least 2 million cells were washed in PBS. DNA was isolated using NEB Monarch Genomic DNA Purification kit. Dry DNA pellets (at least 1.5 µg per sample) were sent to Sequanta Technologies for whole-genome sequencing. Next-generation sequencing libraries were prepared with the Illumina Truseq DNA PCR-free kit and sequenced on the Novaseq6000 platform aiming for 90 G (gigabases per sample) of reads over Q30 quality.

### RNA isolation and sequencing library preparation for expression profiling

Cell line-derived xenograft samples for expression profiling were prepared with either the QuantSeq or TruSeq protocol. QuantSeq libraries were prepared as previously described^23^. In brief, cells were lysed in TRI Lysis Reagent (QIAGEN, 79306) according to the manufacturer’s instructions. Instead of chloroform, 10% volume 1-bromo-2-chloropropane (Sigma-Aldrich, B9673) was added. Total RNA was isolated with RNAeasy Mini kit (QIAGEN, 73404). QuantSeq libraries were prepared using the QuantSeq 3′ mRNA-Seq Library Prep kit FWD for Illumina from Lexogen (015.96) according to the manufacturer’s instructions. Samples were subsequently sequenced on an Illumina NextSeq 500 System with a single-end 76 bp protocol. For TruSeq, a similar RNA isolation protocol was used and RNA-seq libraries were prepared using TruSeq RNA library preparation kit v2 according to the manufacturer’s instructions.

### Data analyses

Statistical analyses were performed with R v.4.0.2 and Bioconductor 3.7 or GraphPad Prism (v.9.3.1). Associations of gene mutations with the sensitivity status of cell lines as well as comparisons of TVs between control and experimental groups were analyzed with a Fisher’s exact test. All other data meeting the requirements for parametric analyses were assessed with paired Student’s *t*-tests or one-way analysis of variance. All statistical analyses used absolute values when calculating TV. Datasets that deviated from normal distribution were analyzed with a nonparametric two-sided Wilcoxon rank-sum test. When applicable, *P* values were adjusted for multiple comparisons according to Bonferroni–Holm, Benjamini–Hochberg FDR analysis or Tukey’s multiple comparisons test. The level of significance was fixed at α = 5% such that an (adjusted) *P* value <0.05 was considered to be statistically significant. Differences were observed to be indicative whenever 0.05 ≤ *P* < 0.10.

Gene expression (RNA-seq) analysis was performed as previously described^23^. In brief, reads from grafted samples were filtered into human and mouse reads using Disambiguate^64^. The filtered reads were then processed with a pipeline by building upon the implementation of the ENCODE ‘Long RNA-seq’ pipeline; filtered reads were mapped against the *Homo* *sapiens* (human) genome hg38/GRCh38 (primary assembly, excluding alternate contigs) or the *Mus* *musculus* (mouse) genome mm10/GRCm38 using the STAR (v.2.5.2b)^65^ aligner allowing for soft clipping of adaptor sequences. For quantification, we used transcript annotation files from Ensembl v.86, which corresponds to GENCODE 25 for human and GENCODE M11 for mouse. Samples were quantified with the above annotations, using RSEM (v.1.3.0) and featureCount (v.1.5.1)^66^. Quality controls were implemented using FastQC (v.0.11.5), picardmetrics (v.0.2.4) and dupRadar (v.1.0)^67^ at the respective steps.

Differential expression analysis was performed on the human mapped counts derived from featureCounts using DESeq2 (v.1.28.1)^68^. We used an absolute log_2_ fold change cutoff of 1.3 and FDR < 0.05.

GSEA was performed using fgsea^69^ R/Bioconductor (v.1.14) package and hallmark gene sets from the molecular signatures database, MsigDB v.7.5.1 (ref. ^70^). The resulting nominal *P* values were adjusted using the Benjamini–Hochberg multiple testing correction method and gene sets with adjusted *P* values <0.01 were considered as significant.

Heatmaps of GSEA were generated using ComplexHeatmap (v.2.4.3)^71^ and upset plots were generated using UpSetR (v.1.4.0)^72^ R/Bioconductor packages. All other figures from data analyses were visualized using ggplot2 (v.3.3.2)^73^ R package.

MPAS^31^ were computed using ten genes previously published (PHLDA1, SPRY2, SPRY4, DUSP4, DUSP6, CCND1, EPHA2, EPHA4, ETV4 and ETV5) and single-sample gene set scoring using GSVA R/bioconductor package^74^. MPAS scores were computed for SW837, NCI-H2122 xenografts models treated with mono and combination treatments and published datasets^75^ treated with KRAS^G12C^i (Extended Data Fig. 10e).

### Analysis of previously reported mutations associated with adagrasib resistance

Whole-genome sequencing reads from NCI-H358 models were aligned to the hg38 human reference genome using bwa to generate BAM files. In the next step, we performed a supervised analysis of previously reported mutations associated with adagrasib resistance in patients (Supplementary Tables 10 and 12). Using BAM files, we generated pileup of reads using pysam (v.0.22.0; https://github.com/pysam-developers/pysam) at each position of these mutations and calculated the percentage of reads supporting reference and alternate allele.

### Statistics and reproducibility

For all the comparison and correlation analyses, exact *P* values are reported in the figures. Unless otherwise specified, *P*_adj_ or FDR means-adjusted *P* values with the Benjamini–Hochberg method and two-sided Wilcoxon rank-sum test was performed. Data distributions were assumed to be normal, but this was not formally tested. Unless otherwise specified, all values were included, median and interquartile ranges were shown in all boxplots with whiskers extending 1.5 × interquartile range. For western blots on NCI-H2122 (Fig. 3c) and SW837 (Extended Data Fig. 3) cell lines, results were replicated using the KRAS^G12C^i, BI-1823911 and the former front-runner SOS1i candidate, BI-17101963.

### Reporting summary

Further information on research design is available in the Nature Portfolio Reporting Summary linked to this article.

## Supplementary information

Reporting Summary

Supplementary Tables 1–13 Supplementary Table 1: results from high-throughput compound screen to identify additive partners of two KRAS^G12C^ inhibitors (sotorasib/AMG 510 and Adagrasib/MRTX849). Supplementary Table 2: mutations observed in LU65, NCI-H1373, NCI-H2122, NCI-H358, SW837, HOP-62, KYSE-410, NCI-H1792, NCI-H2030, SW 1573, HCC-44 cell lines. Supplementary Table 3: pharmacokinetic (PK) analyses in NCI-H2122 xenograft models used in efficacy experiments. Supplementary Table 4: lung and colorectal cancer mouse model body weights. Supplementary Table 5: CRC PDX model mouse body weights. Supplementary Table 6: differentially expressed genes between vehicle control and different mono and combination treatments in NCI-H2122 xenograft models. *P* values are computed with two-sided Wald test in DESeq2 and adjusted for multiple comparisons using Benjamini–Hochberg method. Supplementary Table 7: differentially expressed genes between vehicle control and different mono and combination treatments in SW837 xenograft models. *P* values are computed with two-sided Wald test in DESeq2 and adjusted for multiple comparisons using Benjamini–Hochberg method. Supplementary Table 8: PK analyses in NCI-H2122 models used for biomarker studies Supplementary Table 9: comparison of single adagrasib treatment versus combination with BI-3406 or TNO155 on MIA PaCa-2, NCI-H358, or SW837 parental or clones (cl) harboring additional mutations at KRAS position R68S, H95R or Y96D. Supplementary Table 10: previously reported genetic mutations associated with adagrasib resistance in patients and their status in adagrasib-resistant NCI-H358 xenograft models (evaluated with whole-genome sequencing). Supplementary Table 11: adagrasib-resistant model mouse body weights. Supplementary Table 12: previously reported genetic mutations associated with adagrasib resistance in patients and their status in adagrasib-resistant SW837 xenograft models (evaluated with RNA-seq). Supplementary Table 13: differentially expressed genes between pre-treated and adagrasib-resistant SW837 xenograft models. *P* values are computed with two-sided Wald test in DESeq2 and adjusted for multiple comparisons using Benjamini–Hochberg method.

## Source data

Source Data Unprocessed western blots/gels.

Source Data Fig. 1 Statistical source data.

Source Data Fig. 2 Statistical source data.

Source Data Fig. 3 Statistical source data.

Source Data Fig. 4 Statistical source data.

Source Data Fig. 5 Statistical source data.

Source Data Fig. 6 Statistical source data.

Source Data Extended Data Fig. 1 Statistical source data.

Source Data Extended Data Fig. 2 Statistical source data.

Source Data Extended Data Fig. 4 Statistical source data.

Source Data Extended Data Fig. 5 Statistical source data.

Source Data Extended Data Fig. 6 Statistical source data.

Source Data Extended Data Fig. 7 Statistical source data.

Source Data Extended Data Fig. 8 Statistical source data.

Source Data Extended Data Fig. 9 Statistical source data.

Source Data Extended Data Fig. 10 Statistical source data.

## Data Availability

Cell line-derived xenograft RNA-seq data analyzed in this study were deposited to the Gene Expression Omnibus under the identifier GSE225061. Data for all replicates for each figure are provided in Source Data files. All other data supporting the findings of this study are available from the corresponding author on reasonable request. Source data are provided with this paper.

## Code availability

All the computational analyses were performed using R statistical programming language (v.4.0.2) with open-source packages described in the Methods. No custom code or algorithm has been written for the analyses in our study.
