# Supplementary material for: Co-targeting SOS1 enhances the antitumor effects of KRASG12C inhibitors by addressing intrinsic and acquired resistance
Source: Nat Cancer. 2024 Aug 5;5(9):1352–70. doi: 10.1038/s43018-024-00800-6 (PMC11424490; doi:10.1038/s43018-024-00800-6)
Supplement: Supplementary file 1 — Reporting Summary [file 43018_2024_800_MOESM1_ESM.pdf]

nature portfolio

Corresponding author(s): Dr. Marco H Hofmann

Last updated by author(s): Jun 18, 2024

Reporting Summary

Nature Portfolio wishes to improve the reproducibility of the work that we publish. This form provides structure for consistency and transparency in reporting. For further information on Nature Portfolio policies, see our Editorial Policies and the Editorial Policy Checklist.

Statistics

For all statistical analyses, confirm that the following items are present in the figure legend, table legend, main text, or Methods section.

n/a	Confirmed	
☐	☒	The exact sample size (*n*) for each experimental group/condition, given as a discrete number and unit of measurement
☐	☒	A statement on whether measurements were taken from distinct samples or whether the same sample was measured repeatedly
☐	☒	The statistical test(s) used AND whether they are one- or two-sided<br>*Only common tests should be described solely by name; describe more complex techniques in the Methods section.*
☒	☐	A description of all covariates tested
☐	☒	A description of any assumptions or corrections, such as tests of normality and adjustment for multiple comparisons
☐	☒	A full description of the statistical parameters including central tendency (e.g. means) or other basic estimates (e.g. regression coefficient) AND variation (e.g. standard deviation) or associated estimates of uncertainty (e.g. confidence intervals)
☐	☒	For null hypothesis testing, the test statistic (e.g. *F*, *t*, *r*) with confidence intervals, effect sizes, degrees of freedom and *P* value noted<br>*Give P values as exact values whenever suitable.*
☒	☐	For Bayesian analysis, information on the choice of priors and Markov chain Monte Carlo settings
☐	☒	For hierarchical and complex designs, identification of the appropriate level for tests and full reporting of outcomes
☒	☐	Estimates of effect sizes (e.g. Cohen's *d*, Pearson's *r*), indicating how they were calculated

Our web collection on statistics for biologists contains articles on many of the points above.

Software and code

Policy information about availability of computer code

Data collection	inForm Software v2.4 (Akoya Biosciences); Data generated as part of this study is made publicly available on GEO under the id GSE225061.
Data analysis	R statistical programming (v 4.0.2), Bioconductor (v 3.7), STAR alignment tool (v 2.5.2b), featureCount (v 1.5.1), FastQC (v 0.11.5), picardmetrics (v 0.2.4), dupRadar (v 1.0), DESeq2 (v 1.28.1), fgsea (v 1.14), MsigDB (v 7.5.1), ComplexHeatmap (v 2.4.3), UpSetR (v 1.4.0), ggplot2 (v 3.3.2), pysam (v 0.22.0), HALO 3.6.4134 image analysis (Indica Labs), Graphpad Prism 10.1.2, Incucyte 2019B software,

For manuscripts utilizing custom algorithms or software that are central to the research but not yet described in published literature, software must be made available to editors and reviewers. We strongly encourage code deposition in a community repository (e.g. GitHub). See the Nature Portfolio guidelines for submitting code & software for further information.

Data

Policy information about availability of data

All manuscripts must include a data availability statement. This statement should provide the following information, where applicable:

- Accession codes, unique identifiers, or web links for publicly available datasets
- A description of any restrictions on data availability
- For clinical datasets or third party data, please ensure that the statement adheres to our policy

Cell line-derived xenograft RNA-seq data analyzed in this study were deposited to GEO under the identifier GSE225061. Data for all replicates for each figure are provided in Source Data files. All other data supporting the findings of this study are available from the corresponding author on reasonable request.

Research involving human participants, their data, or biological material

Policy information about studies with human participants or human data. See also policy information about sex, gender (identity/presentation), and sexual orientation and race, ethnicity and racism.

Reporting on sex and gender	n/a
Reporting on race, ethnicity, or other socially relevant groupings	n/a
Population characteristics	n/a
Recruitment	n/a
Ethics oversight	n/a

Note that full information on the approval of the study protocol must also be provided in the manuscript.

Field-specific reporting

Please select the one below that is the best fit for your research. If you are not sure, read the appropriate sections before making your selection.

☒ Life sciences ☐ Behavioural & social sciences ☐ Ecological, evolutionary & environmental sciences

For a reference copy of the document with all sections, see nature.com/documents/nr-reporting-summary-flat.pdf

Life sciences study design

All studies must disclose on these points even when the disclosure is negative.

Sample size	The sample size was determined based on our previous experience and power calculations as described in Zheng et al., 2013 (PMID: 23845442). For in vivo experiments sample sizes were determined based on Jackson et al., 2005 (PMID: 16288016) and were selected to be sufficient to enable statistical significance evaluation. No statistical method was used to determine sample sizes.<br><br>Only female mice were used in efficacy studies for husbandry purposes. This study was focused on NSCLC and CRC cell lines. The hormone dependency is lower in NSCLC and CRC tumor cells compared to indications driven by the aberrant signaling of reproductive hormones, such as breast and prostate cancer.
Data exclusions	No data were excluded.
Replication	Each in vivo experiment presented in the paper was repeated in multiple mice (n > 5 per arm). Technical replicates for in vitro experiments were done with n=3. -In vivo experiments were not repeated due to ethical reasons but animals per group are indicated in figure legends. At least 3 independent experiments were performed for in vitro assays with consistent results, with the following exceptions:<br>- in vitro experiments using B8219, B8182, and F3008 cells were each repeated in 2 independent experiments showing similar results<br>- For Western blots on NCI-H2122 (Fig 3c) and SW837 (Extended Data Fig 3) cell lines, similar results were obtained using the KRASG12Ci , BI 1823911, and the former front-runner SOS1i candidate, BI 17101963 (Rebuttal Fig. 6)<br>- Extended data Fig 7a was only conducted once using a pool of cells generated with the help of a single site variant library to KRASG12C.
Randomization	Included in text. For in vivo experiments, when tumor sizes reached approximately 150-250mm3, animals were randomized based on tumor sizes and assigned to different experimental groups. For in vitro experiments, samples were randomly allocated into experimental groups
Blinding	Data collection was only partially blinded manner as compound codes were used by the experimenters but some experimenters knew the compound names behind the compound codes due to repetition of experiments. Data analysis was not done in a blinded manner as compound names were available at this step

Reporting for specific materials, systems and methods

We require information from authors about some types of materials, experimental systems and methods used in many studies. Here, indicate whether each material, system or method listed is relevant to your study. If you are not sure if a list item applies to your research, read the appropriate section before selecting a response.

Materials & experimental systems

n/a	Involved in the study
	☒ Antibodies
	☒ Eukaryotic cell lines
☒	☐ Palaeontology and archaeology
	☒ Animals and other organisms
☒	☐ Clinical data
☒	☐ Dual use research of concern
☒	☐ Plants

Methods

n/a	Involved in the study
☒	☐ ChIP-seq
☒	☐ Flow cytometry
☒	☐ MRI-based neuroimaging

Antibodies

Antibodies used

Antibodies were used for Western blots (not following Ras pull-down): primary antibodies against KRAS (LSBio #LS-C175665; 1:500), pERK Thr202/Tyr204 (Cell Signaling #4376; 1:500), ERK (Cell Signaling #9102; 1000-1:2000), phospho-S6 Ribosomal Protein (Ser235/236) (Cell Signaling #2211; 1:500- 1:1000), DUSP-6 (Abcam #ab76310; 1:1000), cleaved PARP (Asp214) (Cell Signaling #9541; 1:1000), Cyclin D1 (Biosite #ARB-Q4OL25-0,5; 1:100); p27 (BD #610241; 1:1000), and ß-Actin (abcam #ab8226; 1:10000); secondary antibodies were diluted in respective incubation buffers: goat a-rabbit IgG, HRP conjugated (Dako #P0448; 1:1000), goat a-mouse IgG, HRP conjugated (Dako #P0447; 1:1000). Antibodies used for western blotting following the RAS pull-down: KRAS (1:1000; LSBio #LS-C175665), HRAS (1:500; Proteintech #18295-1-AP), MRAS (1:200; abcam #ab176570), NRAS (1:1000; abcam #ab167136), and GAPDH (1:1000; Cell Signaling #2118). For WES capillary immunoassay: MRAS (Anti-MRas antibody [EPR12457], rabbit #ab176570, Abcam, 1/50 dilution); vimentin (Vimentin (D21H3) XP, rabbit, 1/50 dilution, #5741, Cell Signaling), E-Cadherin (ab231303, mouse, 1/100 dilution, Abcam), α-Actinin (1/200 dilution, rabbit, #3134, Cell Signaling); Protein Simple (Anti-Mouse Detection Module, #DM-002 and Anti-Rabbit Detection Module, #DM-001). For biomarker analyses: Anti-phospho-p44/42 MAPK (ERK1/2) (Thr202/Tyr204) (1:2000, Cell Signaling Technology #4370), Alexa 647 (1:250, Abcam #199837); anti-phospho-p44/42 MAPK (ERK1/2) (Thr202/Tyr204), anti-KI-67 (CST #9027, 1/400 in PBS/2% BSA).

Validation

We only used commercially available antibodies. Validation experiments for all antibodies can thus be found on their respective websites:
KRAS (LSBio #LS-C175665): https://www.lsbio.com/antibodies/kras-antibody-clone-2c1-ihc-wb-western-ls-c175665/183089#validation-section
ERK Thr202/Tyr204 (Cell Signaling #4376): https://www.cellsignal.com/products/primary-antibodies/phospho-p44-42-mapk-erk1-2-thr202-tyr204-20g11-rabbit-mab/4376
ERK (Cell Signaling #9102): https://www.cellsignal.com/products/primary-antibodies/p44-42-mapk-erk1-2-antibody/9102
phospho-S6 Ribosomal Protein (Ser235/236) (Cell Signaling #2211): https://www.cellsignal.com/products/primary-antibodies/phospho-s6-ribosomal-protein-ser235-236-antibody/2211
DUSP-6 (Abcam #ab76310): https://www.abcam.com/products/primary-antibodies/dusp6-antibody-epr129y-ab76310.html
cleaved PARP (Asp214) (Cell Signaling #9541): https://www.cellsignal.com/products/primary-antibodies/cleaved-parp-asp214-antibody/9541
Cyclin D1 (Biosite #ARB-Q4OL25-0,5): validated by Cheuk et al 2004 PMID 15166673
p27 (BD #610241): https://www.bdbiosciences.com/en-us/products/reagents/microscopy-imaging-reagents/immunofluorescence-reagents/purified-mouse-anti-p27-kip1.610241
ß-Actin (abcam #ab8226): https://www.abcam.com/products/primary-antibodies/beta-actin-antibody-mabcam-8226-loading-control-ab8226.html
goat a-rabbit IgG, HRP conjugated (Dako #P0448): https://www.agilent.com/en/product/specific-proteins/elisa-kits-accessories/goat-anti-rabbit-immunoglobulins-hrp-affinity-isolated-2717113
goat a-mouse IgG, HRP conjugated (Dako #P0447): https://www.agilent.com/en/product/specific-proteins/elisa-kits-accessories/goat-anti-mouse-immunoglobulins-hrp-affinity-isolated-2717109
HRAS (Proteintech #18295-1-AP): https://www.ptglab.com/products/HRAS-Specific-Antibody-18295-1-AP.htm
MRAS (abcam #ab176570): https://www.abcam.com/products/primary-antibodies/mras-antibody-epr12457-ab176570.html
NRAS (abcam #ab167136): https://www.abcam.com/products/primary-antibodies/nras-antibody-ab167136.html
GAPDH (Cell Signaling #2118): https://www.cellsignal.com/products/primary-antibodies/gapdh-14c10-rabbit-mab/2118
Vimentin D21H3 (#5741, Cell Signaling): https://www.cellsignal.com/products/primary-antibodies/vimentin-d21h3-xp-174-rabbit-mab/5741
E-Cadherin (ab231303, Abcam): https://www.abcam.com/products/primary-antibodies/e-cadherin-antibody-4a2-ab231303.html
α-Actinin (#3134, Cell Signaling): https://www.cellsignal.com/products/primary-antibodies/a-actinin-antibody/3134
Protein Simple Anti-Mouse Detection Module, #DM-002: https://www.bio-techne.com/p/simple-western/anti-mouse-detection-module_dm-002
Protein Simple Anti-Rabbit Detection Module, #DM-001: https://www.bio-techne.com/p/simple-western/anti-rabbit-detection-module_dm-001
Anti-phospho-p44/42 MAPK (Cell Signaling Technology #4370): https://www.cellsignal.com/products/primary-antibodies/phospho-p44-42-mapk-erk1-2-thr202-tyr204-d13-14-4e-xp-rabbit-mab/4370
Alexa 647 (Abcam #199837): https://www.abcam.com/products/primary-antibodies/alexa-fluor-647-hla-a-antibody-ep1395y-ab199837.html
anti-phospho-p44/42 MAPK (ERK1/2) (Thr202/Tyr204),
anti-KI-67 (CST #9027, 1/400 in PBS/2% BSA): https://www.cellsignal.com/products/primary-antibodies/phospho-p44-42-mapk-erk1-2-thr202-tyr204-antibody/9101

Eukaryotic cell lines

Policy information about cell lines and Sex and Gender in Research

Cell line source(s)	All cell lines except for LKR13 lines (LKR13K, LKR13KL, and LKR13KK) were obtained from the American Type Culture Collection (ATCC). The LKR13 cells were gifted by Dr. John Heymach at MD Anderson Cancer Center. All other cell lines were purchased from American Type Culture Collection (ATCC), including NCI-H2122 (ATCC CRL-5985), SW837 (ATCC CCL-235), NCI-H358 (ATCC CRL-5807). Sex was not considered in studies using cell lines. We used tumor cell lines that were derived from female and male patients: NCI-H2122 (f), SW837 (m), MIA PaCa-2 (m), NCI-H358 (m), NCI-H1373 (m), HOP62 (f), HCC-44 (f), NCI-H2023 (m), KYSE-410 (m), LU65 (f), SW1573 (f), NCI-1792 (m), F3008 (f), B8032 (m), B8219 (m), and B8182 (f).
Authentication	All cells were authenticated using STR profiling and whole genome sequencing.
Mycoplasma contamination	Cell lines were tested negative for mycoplasma contamination.
Commonly misidentified lines (See ICLAC register)	No commonly misidentified cells were used.

Animals and other research organisms

Policy information about studies involving animals; ARRIVE guidelines recommended for reporting animal research, and Sex and Gender in Research

Laboratory animals	Mice at MD Anderson are group housed within environmentally-controlled conditions with 12-hour-light/dark cycle at 21°C-23°C, 40%-60% humidity; all mice recieve LabDiet 5053 chow and sterile water ad libitum. Mice at Boehringer-Ingelheim are group housed within environmentally-controlled conditions with a 12-hour-light/dark cycle at 21°C ± 1.5 °C, 55% ± 10% humidity; all mice receive food and water ad libitum. The F3008 PDX study was performed in female NSG (NOD.Cg-Prkdcscid Il2rgtm1Wjl/SzJ) mice (The Jackson Laboratory Cat# 005557) and the B8032 PDX study was performed in female athymic nude (NU(NCr)-Foxn1nu; strain 490) mice (Charles River Laboratories Cat# 24106219). For efficacy and biomarker studies, 7-10 week old female BomTac:NMRI-Foxn1nu (SW837 and NCI-H2122) or 7 week old female CB-17/Icr-Prkdc scid/Rj (NCI-H358) mice were used. Maximal tumor size/burden for animals housed in Boehringer-Ingelheim or MD Anderson facilities were being greater or equal to 1500 mm3 or 2000 mm3, respectively. Maximal tumor size was not exceeded in any study.
Wild animals	No wild animals were used in the study.
Reporting on sex	Only female mice were used.
Field-collected samples	No field collected samples were used in the study.
Ethics oversight	All procedures for PDX studies were reviewed and approved by the Institutional Animal Care and Use Committee (IACUC #00000884-RN04) at MD Anderson Cancer Center. All animal studies performed at Boehringer-Ingelheim were approved by the internal ethics committee and the local Austrian governmental committee.

Note that full information on the approval of the study protocol must also be provided in the manuscript.

Plants

Seed stocks	n/a
Novel plant genotypes	n/a
Authentication	n/a
