# Supplementary material for: Co-targeting SOS1 enhances the antitumor effects of KRASG12C inhibitors by addressing intrinsic and acquired resistance
Source: Nat Cancer. 2024 Aug 5;5(9):1352–70. doi: 10.1038/s43018-024-00800-6 (PMC11424490; doi:10.1038/s43018-024-00800-6)
Supplement: Supplementary file 3 — Unprocessed western blots/gels. [file 43018_2024_800_MOESM3_ESM.pdf]

Fig 3b

H2122_2h treatment-raw data

KRAS

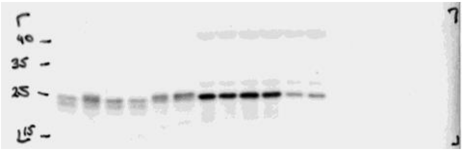

HRAS

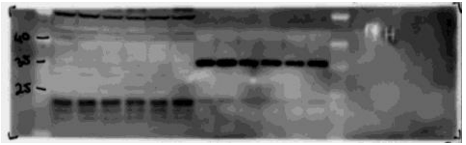

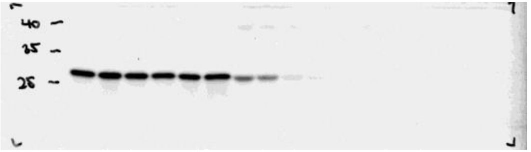

MRAS

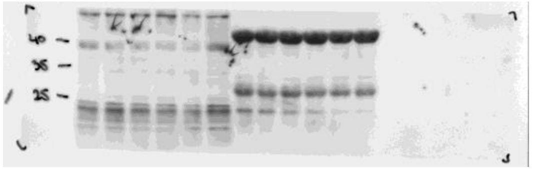

NRAS

1st Antibody	MW [kD]	used dilution	Company	Cat #	2nd Antibody	Company	Cat #
GAPDH	37 kDa	1:1000	Cell Signalling Technologies	#2118	rabbit	Dako	P0448
KRAS	21 kDa	1:1000	LSBio	LS-C175665	mouse	Dako	P0447
HRAS	21 kDa	1:500	proteintech	18295-1-AP	rabbit	Dako	P0448
NRAS	21 kDa	1:1000	abcam	ab167136	rabbit	Dako	P0448
MRAS	21 kDa	1:200	abcam	ab176570	rabbit	Dako	P0448

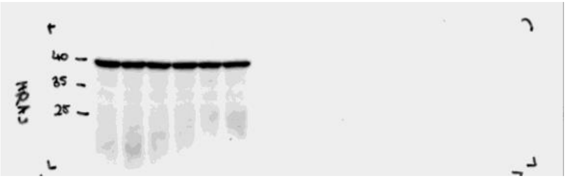

GAPDH

H2122_2h treatment-raw data

pERK

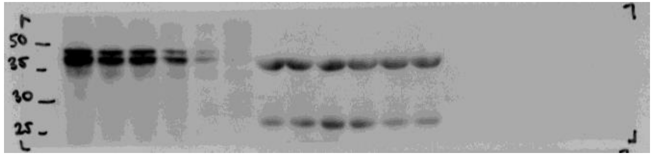

ERK

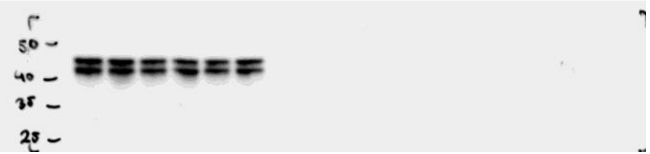

Vinculin

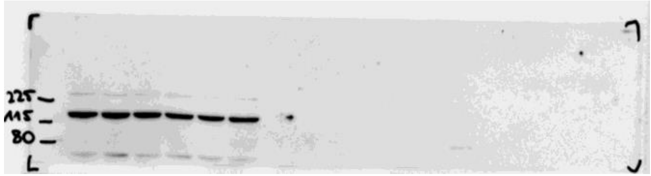

1st Antibody	MW [kD]	used dilution	Incubation	Species	Company	Cat #
Vinculin	124 kDa (it appears at 115 kDa)	1:200	ON at 4°C	rabbit	CST	#4650
pERK	44, 42 kDa	1:1000	ON at 4°C	rabbit	CST	#4370
ERK	44, 42 kDa	1:1000	ON at 4°C	rabbit	CST	#9102

H2122_48h treatment-raw data

KRAS

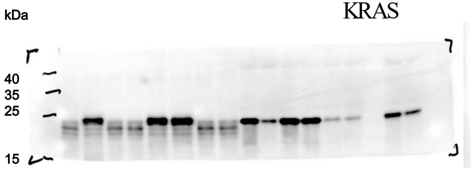

HRAS

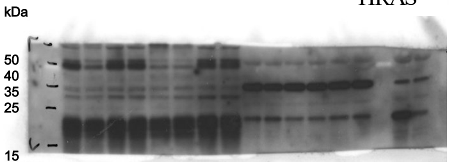

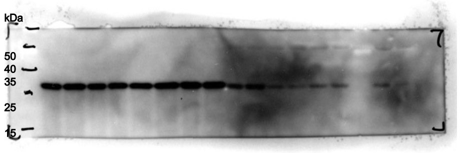

MRAS

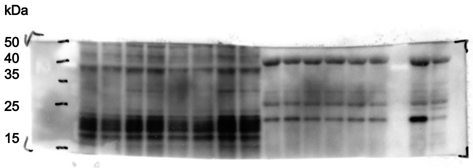

NRAS

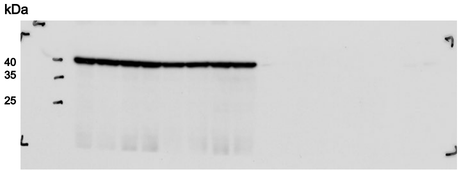

GAPDH

1st Antibody	MW [kD]	used dilution	Company	Cat #	2nd Antibody	Company	Cat #
GAPDH	37 kDa	1:1000	Cell Signalling Technologies	#2118	rabbit	Dako	P0448
KRAS	21 kDa	1:1000	LSBio	LS-C175665	mouse	Dako	P0447
HRAS	21 kDa	1:500	proteintech	18295-1-AP	rabbit	Dako	P0448
NRAS	21 kDa	1:1000	abcam	ab167136	rabbit	Dako	P0448
MRAS	21 kDa	1:200	abcam	ab176570	rabbit	Dako	P0448

H2122_48h treatment-raw data

pERK

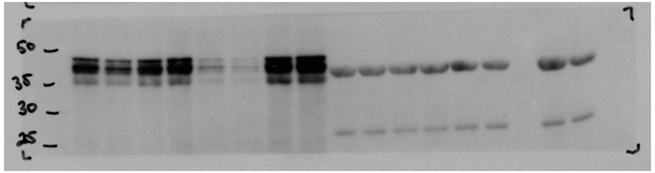

ERK

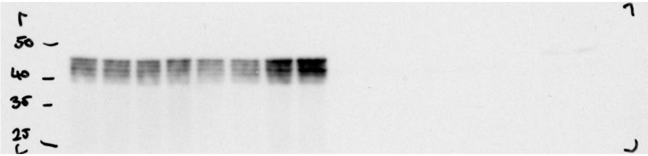

Vinculin

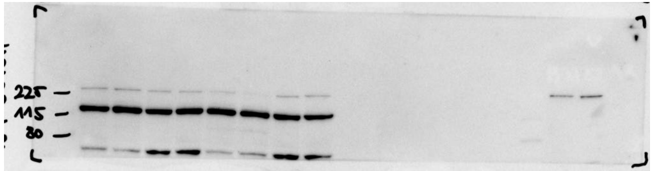

1st Antibody	MW [kD]	used dilution	Incubation	Species	Company	Cat #
Vinculin	124 kDa (it appears at 115 kDa)	1:200	ON at 4°C	rabbit	CST	#4650
pERK	44, 42 kDa	1:1000	ON at 4°C	rabbit	CST	#4370
ERK	44, 42 kDa	1:1000	ON at 4°C	rabbit	CST	#9102

H358_2h treatment - raw data

KRAS

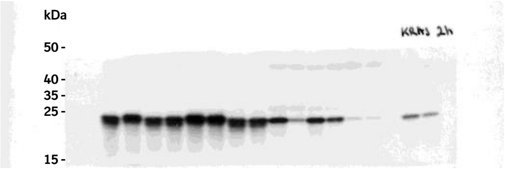

HRAS

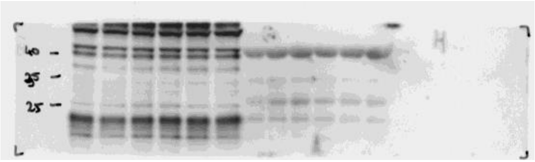

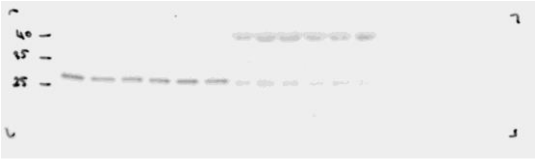

MRAS

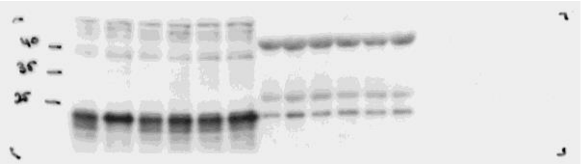

NRAS

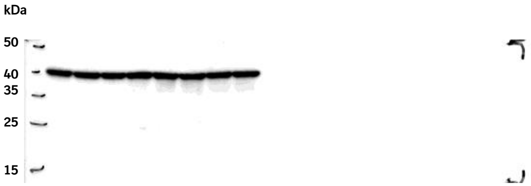

GAPDH

1st Antibody	MW [kD]	used dilution	Company	Cat #	2nd Antibody	Company	Cat #
GAPDH	37 kDa	1:1000	Cell Signalling Technologies	#2118	rabbit	Dako	P0448
KRAS	21 kDa	1:1000	LSBio	LS-C175665	mouse	Dako	P0447
HRAS	21 kDa	1:500	proteintech	18295-1-AP	rabbit	Dako	P0448
NRAS	21 kDa	1:1000	abcam	ab167136	rabbit	Dako	P0448
MRAS	21 kDa	1:200	abcam	ab176570	rabbit	Dako	P0448

H358 2h treatment-raw data

pERK

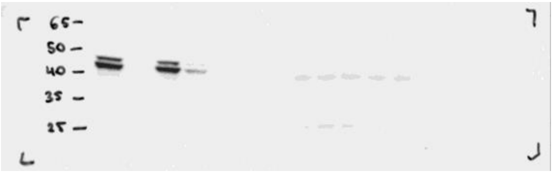

ERK

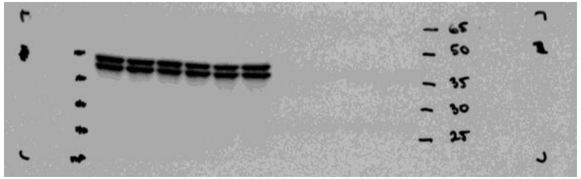

Vinculin

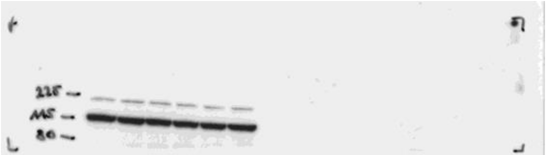

1st Antibody	MW [kD]	used dilution	Incubation	Species	Company	Cat #
Vinculin	124 kDa (it appears at 115 kDa)	1:200	ON at 4°C	rabbit	CST	#4650
pERK	44, 42 kDa	1:1000	ON at 4°C	rabbit	CST	#4370
ERK	44, 42 kDa	1:1000	ON at 4°C	rabbit	CST	#9102

H358_48h treatment - raw data

KRAS

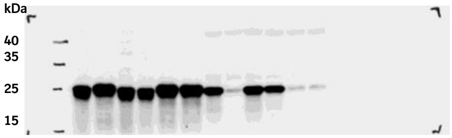

HRAS

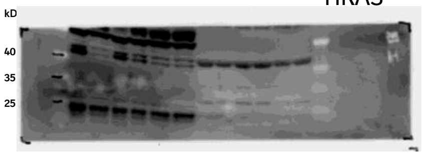

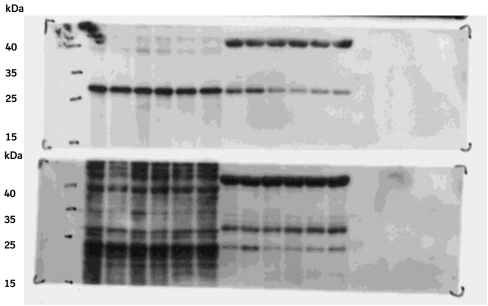

MRAS

NRAS

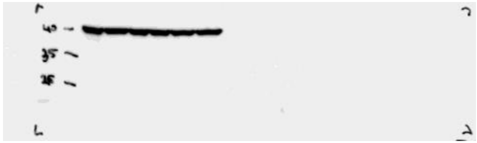

GAPDH

1st Antibody	MW [kD]	used dilution	Company	Cat #	2nd Antibody	Company	Cat #
GAPDH	37 kDa	1:1000	Cell Signalling Technologies	#2118	rabbit	Dako	P0448
KRAS	21 kDa	1:1000	LSBio	LS-C175665	mouse	Dako	P0447
HRAS	21 kDa	1:500	proteintech	18295-1-AP	rabbit	Dako	P0448
NRAS	21 kDa	1:1000	abcam	ab167136	rabbit	Dako	P0448
MRAS	21 kDa	1:200	abcam	ab176570	rabbit	Dako	P0448

H358_48h treatment - raw data

pERK

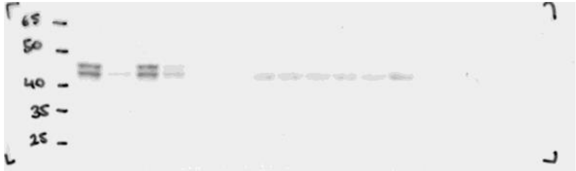

ERK

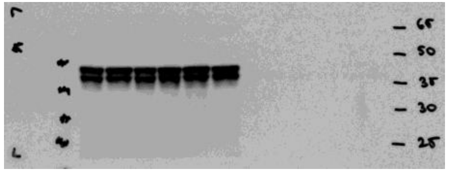

Vinculin

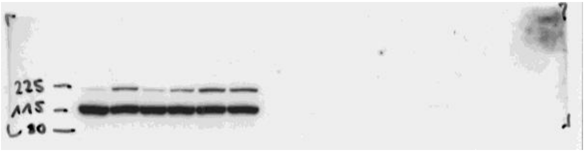

1st Antibody	MW [kD]	used dilution	Incubation	Species	Company	Cat #
Vinculin	124 kDa (it appears at 115 kDa)	1:200	ON at 4°C	rabbit	CST	#4650
pERK	44, 42 kDa	1:1000	ON at 4°C	rabbit	CST	#4370
ERK	44, 42 kDa	1:1000	ON at 4°C	rabbit	CST	#9102

SW837 2h treatment - raw data

KRAS

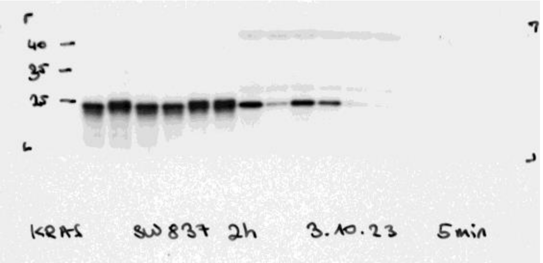

HRAS

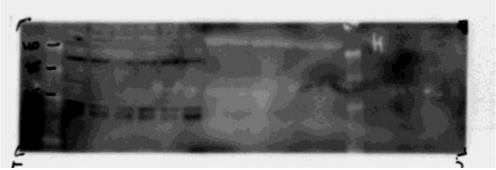

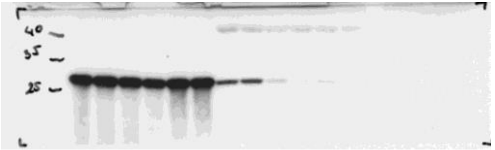

MRAS

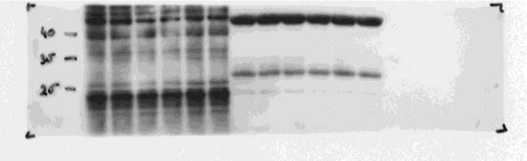

NRAS

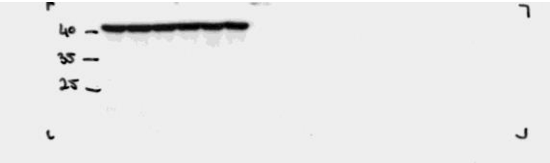

GAPDH

1st Antibody	MW [kD]	used dilution	Company	Cat #	2nd Antibody	Company	Cat #
GAPDH	37 kDa	1:1000	Cell Signalling Technologies	#2118	rabbit	Dako	P0448
KRAS	21 kDa	1:1000	LSBio	LS-C175665	mouse	Dako	P0447
HRAS	21 kDa	1:500	proteintech	18295-1-AP	rabbit	Dako	P0448
NRAS	21 kDa	1:1000	abcam	ab167136	rabbit	Dako	P0448
MRAS	21 kDa	1:200	abcam	ab176570	rabbit	Dako	P0448

SW837 2h treatment - raw data

pERK

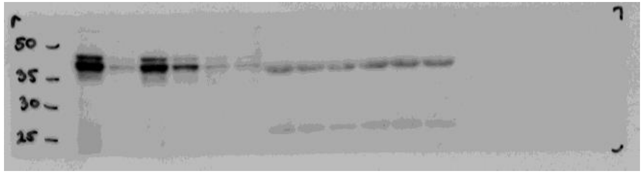

ERK

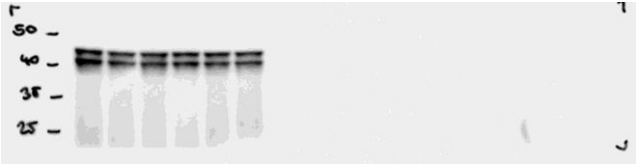

Vinculin

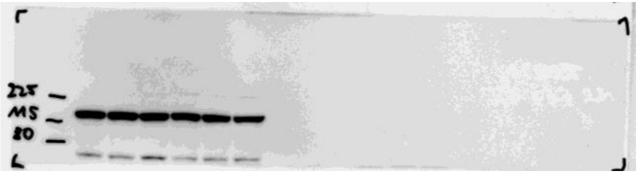

1st Antibody	MW [kD]	used dilution	Incubation	Species	Company	Cat #
Vinculin	124 kDa (it appears at 115 kDa)	1:200	ON at 4°C	rabbit	CST	#4650
pERK	44, 42 kDa	1:1000	ON at 4°C	rabbit	CST	#4370
ERK	44, 42 kDa	1:1000	ON at 4°C	rabbit	CST	#9102

SW837_48h treatment-raw data

KRAS

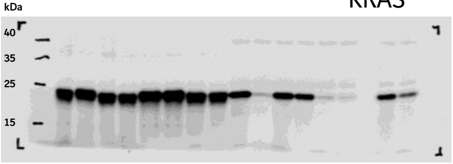

HRAS

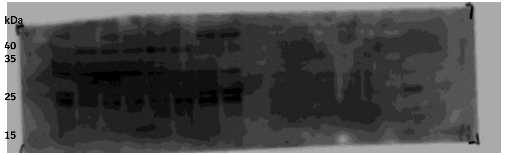

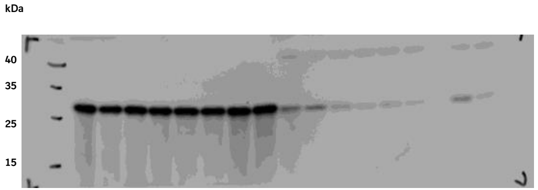

MRAS

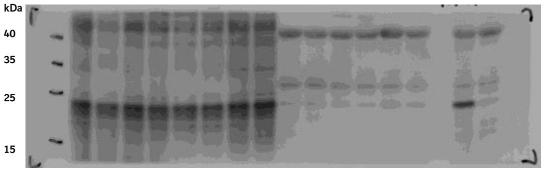

NRAS

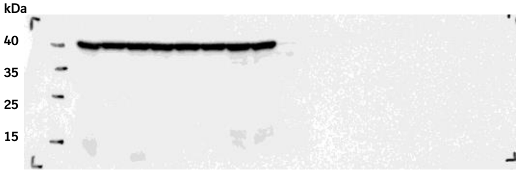

GAPDH

1st Antibody	MW [kD]	used dilution	Company	Cat #	2nd Antibody	Company	Cat #
GAPDH	37 kDa	1:1000	Cell Signalling Technologies	#2118	rabbit	Dako	P0448
KRAS	21 kDa	1:1000	LSBio	LS-C175665	mouse	Dako	P0447
HRAS	21 kDa	1:500	proteintech	18295-1-AP	rabbit	Dako	P0448
NRAS	21 kDa	1:1000	abcam	ab167136	rabbit	Dako	P0448
MRAS	21 kDa	1:200	abcam	ab176570	rabbit	Dako	P0448

SW837_48h treatment-raw data

pERK

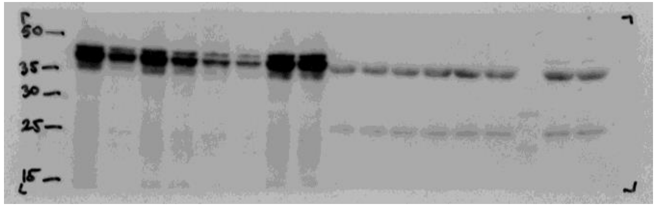

ERK

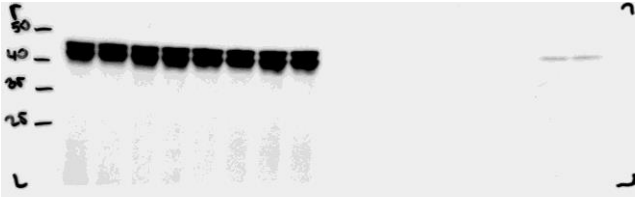

Vinculin

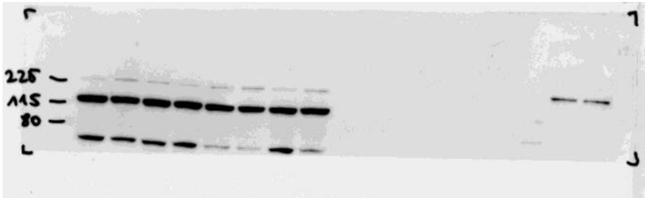

1st Antibody	MW [kD]	used dilution	Incubation	Species	Company	Cat #
Vinculin	124 kDa (it appears at 115 kDa)	1:200	ON at 4°C	rabbit	CST	#4650
pERK	44, 42 kDa	1:1000	ON at 4°C	rabbit	CST	#4370
ERK	44, 42 kDa	1:1000	ON at 4°C	rabbit	CST	#9102

Figure 3c

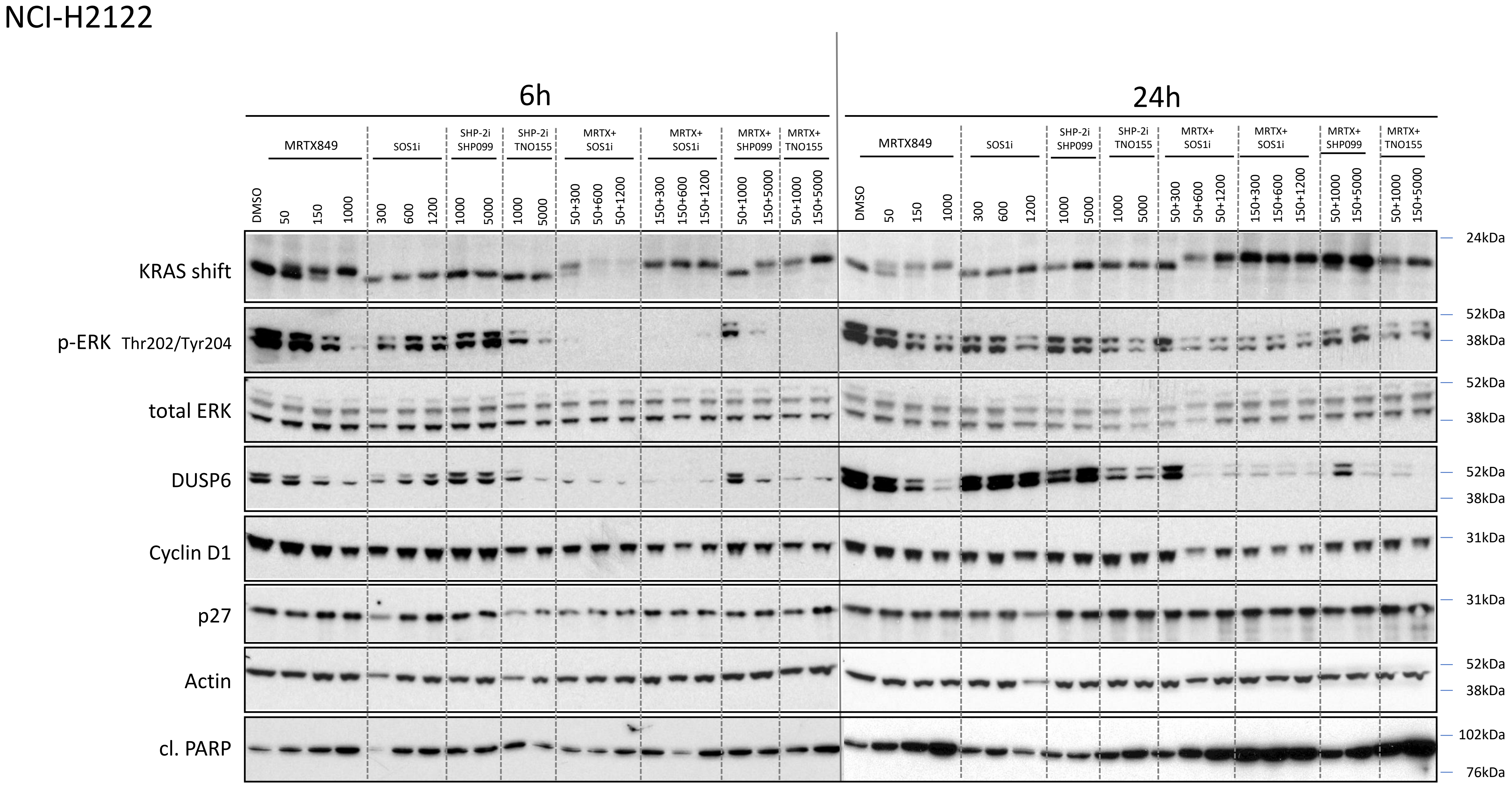
NCI-H2122
6h
24h
MRTX849
SOS1i
SHP-2i SHP099
SHP-2i TNO155
MRTX+ SOS1i
MRTX+ SHP099
MRTX+ TNO155
DMSO
50
150
1000
300
600
1200
5000
50+300
50+600
50+1200
150+300
150+600
150+1200
50+1000
150+5000
50+1000
150+5000
KRAS shift
p-ERK Thr202/Tyr204
total ERK
DUSP6
Cyclin D1
p27
Actin
cl. PARP
24kDa
52kDa
38kDa
31kDa
102kDa
76kDa

Technical Information

- If not otherwise mentioned 20 µg protein / lane loaded
- Same antibody dilutions used for 6 and 24h samples
- Exposure times to detect ECL signals are listed, and same exposures were used for 6 and 24h samples
- To safe extract, antibodies etc. we typically cut membranes to only blot the relevant region of the membrane with the respective antibodies, that allows typically 2-4 antibodies to use per membrane
- X-ray scans were either in total or often only the relevant region scanned
- Note the colour code which indicates correlating membranes
- Sometimes several exposure or several blots are shown, the once taken for the figures are indicated in bold and have a star (*)

NCI-H2122

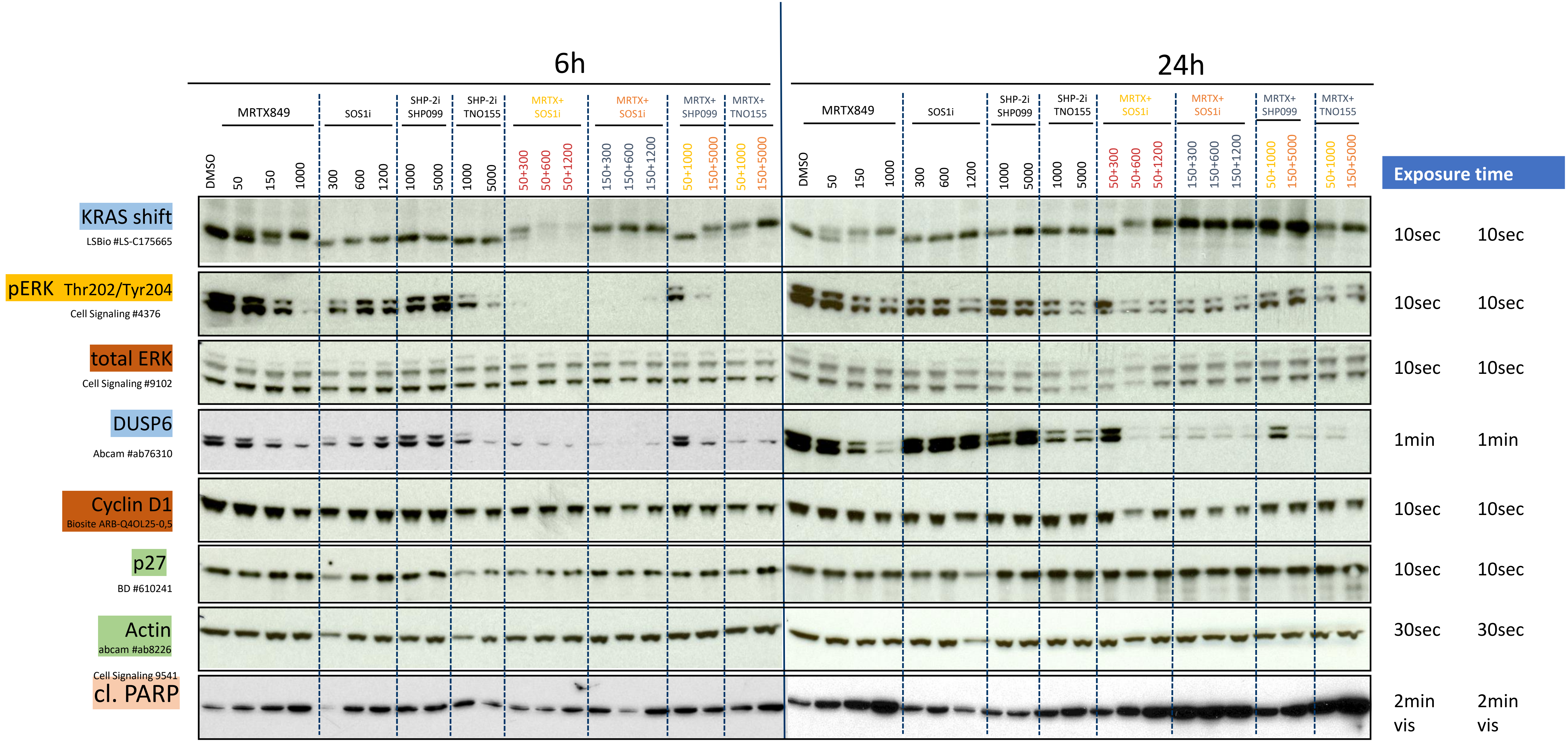
6h
24h
MRTX849
SOS1i
SHP-2i SHP099
SHP-2i TNO155
MRTX+ SOS1i
MRTX+ SHP099
MRTX+ TNO155
DMSO
50
150
1000
300
600
1200
5000
50+300
50+600
50+1200
150+300
150+600
150+1200
50+1000
150+5000
Exposure time
KRAS shift
LSBio #LS-C175665
pERK Thr202/Tyr204
Cell Signaling #4376
total ERK
Cell Signaling #9102
DUSP6
Abcam #ab76310
Cyclin D1
Biosite ARB-Q4OL25-0,5
p27
BD #610241
Actin
abcam #ab8226
Cell Signaling 9541
cl. PARP
10sec 10sec
10sec 10sec
10sec 10sec
1min 1min
10sec 10sec
10sec 10sec
30sec 30sec
2min vis 2min vis

Scanned blots of 6h treatment:

10 seconds exposure - showing how several membrane incubated with various antibodies look on one x-ray

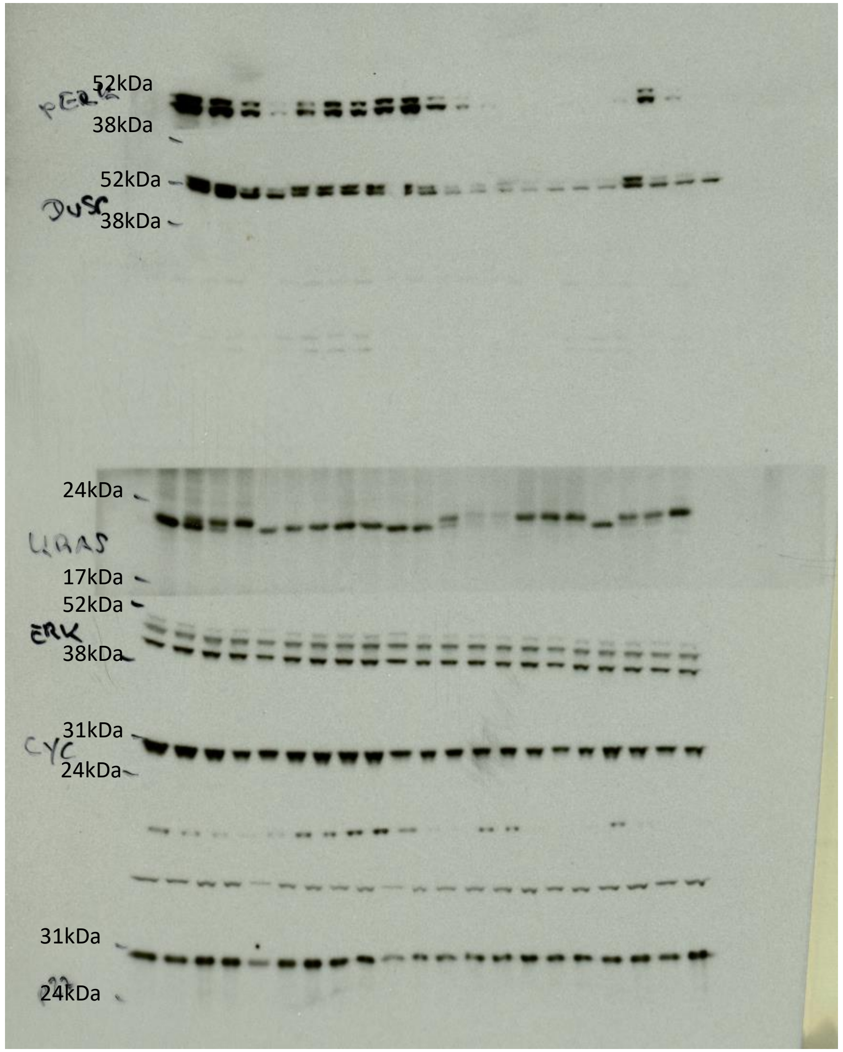

pERK 42/44kDa*

DUSP 6 42/44kDa not used for figure, blot was repeated (air bubble)

KRAS 21,7kDa*

ERK 42/44kDa*

Cyclin D1 36kDa*

Actin 42kDa – not used for figure, 30sec exposure used

p27 27kDa*

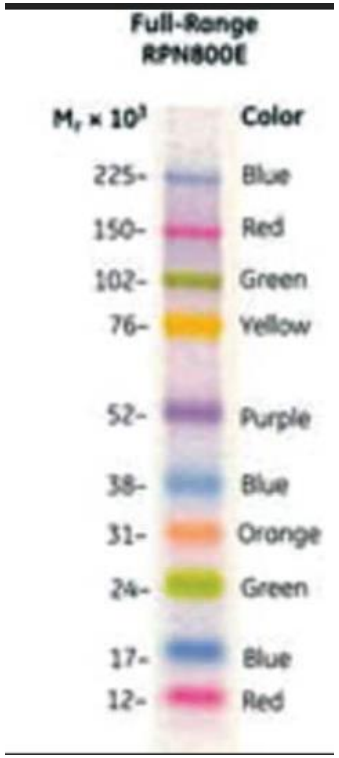

Scanned blots of 6h treatment:

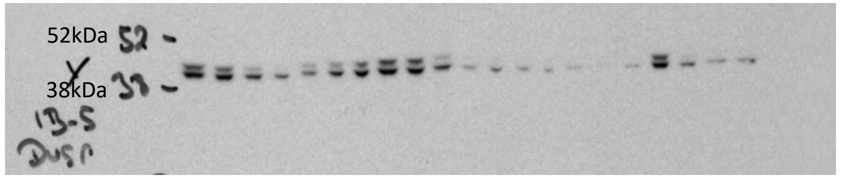

1min exposure

DUSP6* 42/44kDa

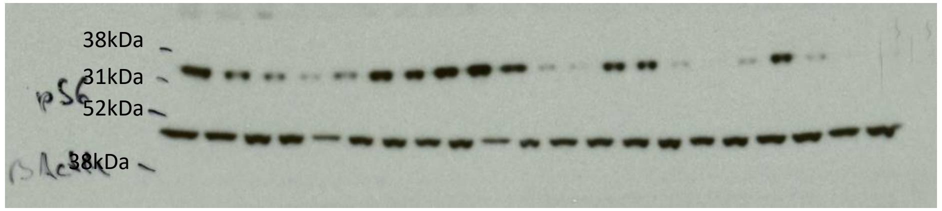

30sec exposure

Actin* 42kDa

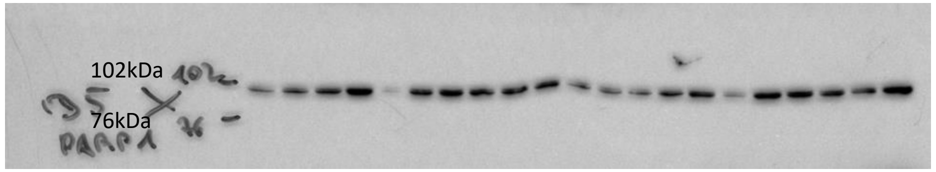

2min Visualizer exposure*

cl-.PARP* 89kDa

Scanned blots of 24h treatment:

1min exposure – showing how several membrane incubated with various antibodies look on one x-ray

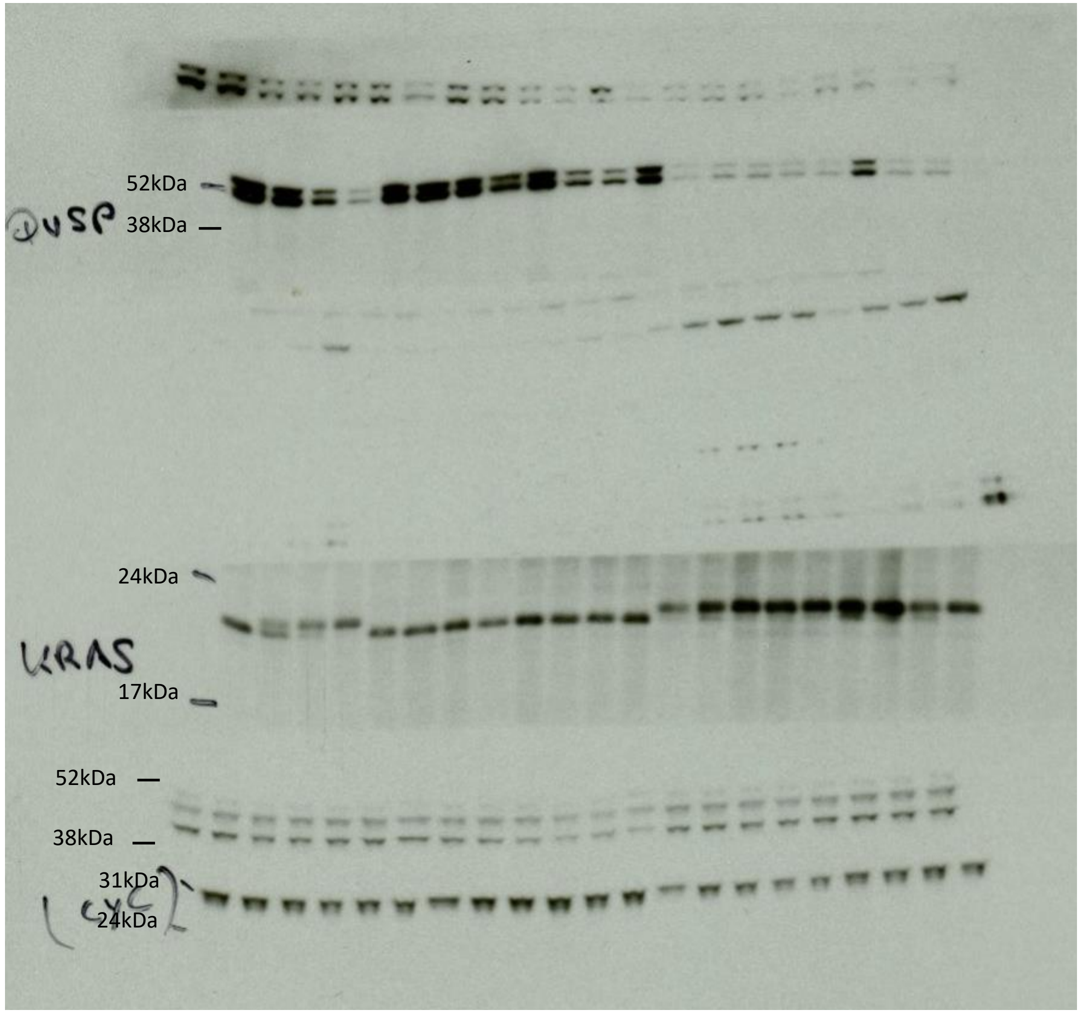

DUSP 6 42/44kDa*

KRAS 21,7kDa (not used 10 sec exp. used)

ERK 42/44kDa (not used 10 sec exp. used)

Cyclin 36kDa (not used, 10 sec exp. used)

Scanned blots of 24h treatment:

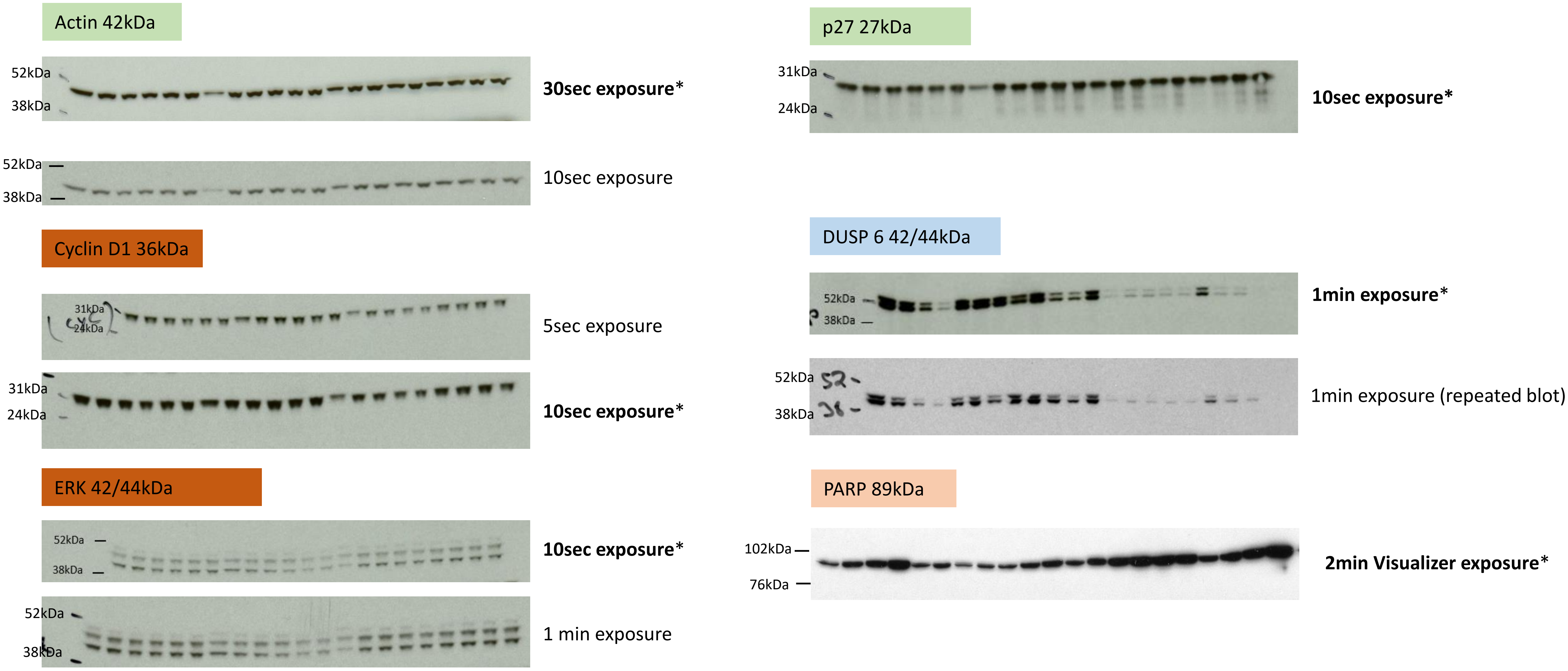

Scanned blots of 24h treatment:

KRAS 21,7kDa

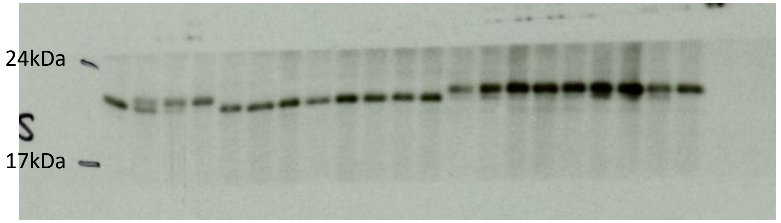

10sec exposure*

pERK 42/44kDa

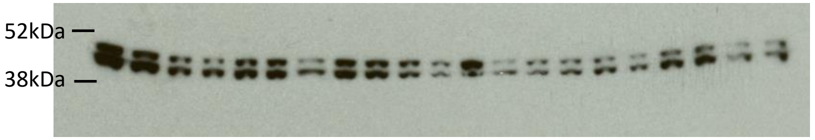

10sec exposure*

Extended Data Fig 1c

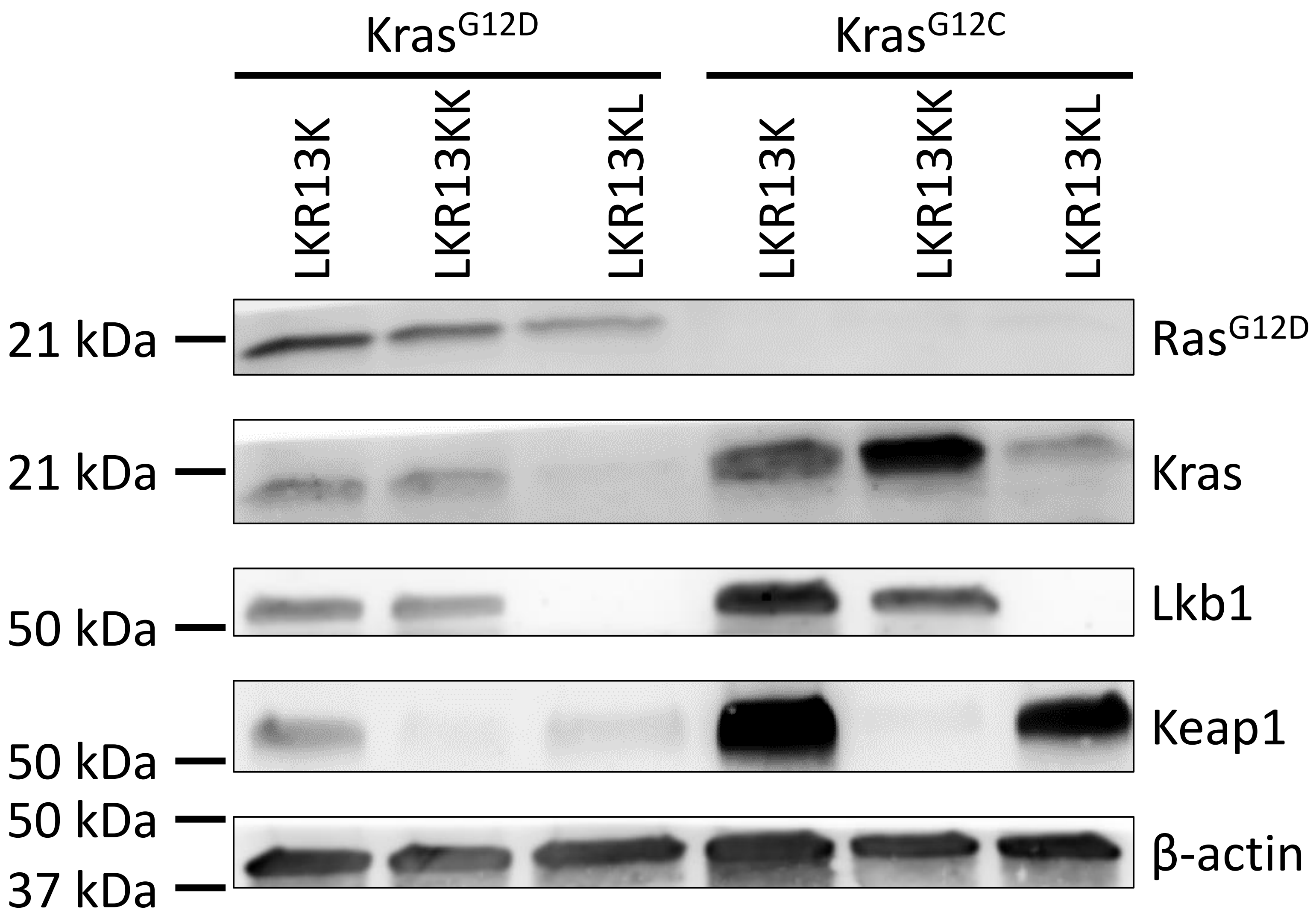

Kras^{G12D}
Kras^{G12C}
LKR13K
LKR13KK
LKR13KL
LKR13K
LKR13KK
LKR13KL
21 kDa
Ras^{G12D}
21 kDa
Kras
Lkb1
50 kDa
Keap1
50 kDa
50 kDa
β-actin
37 kDa

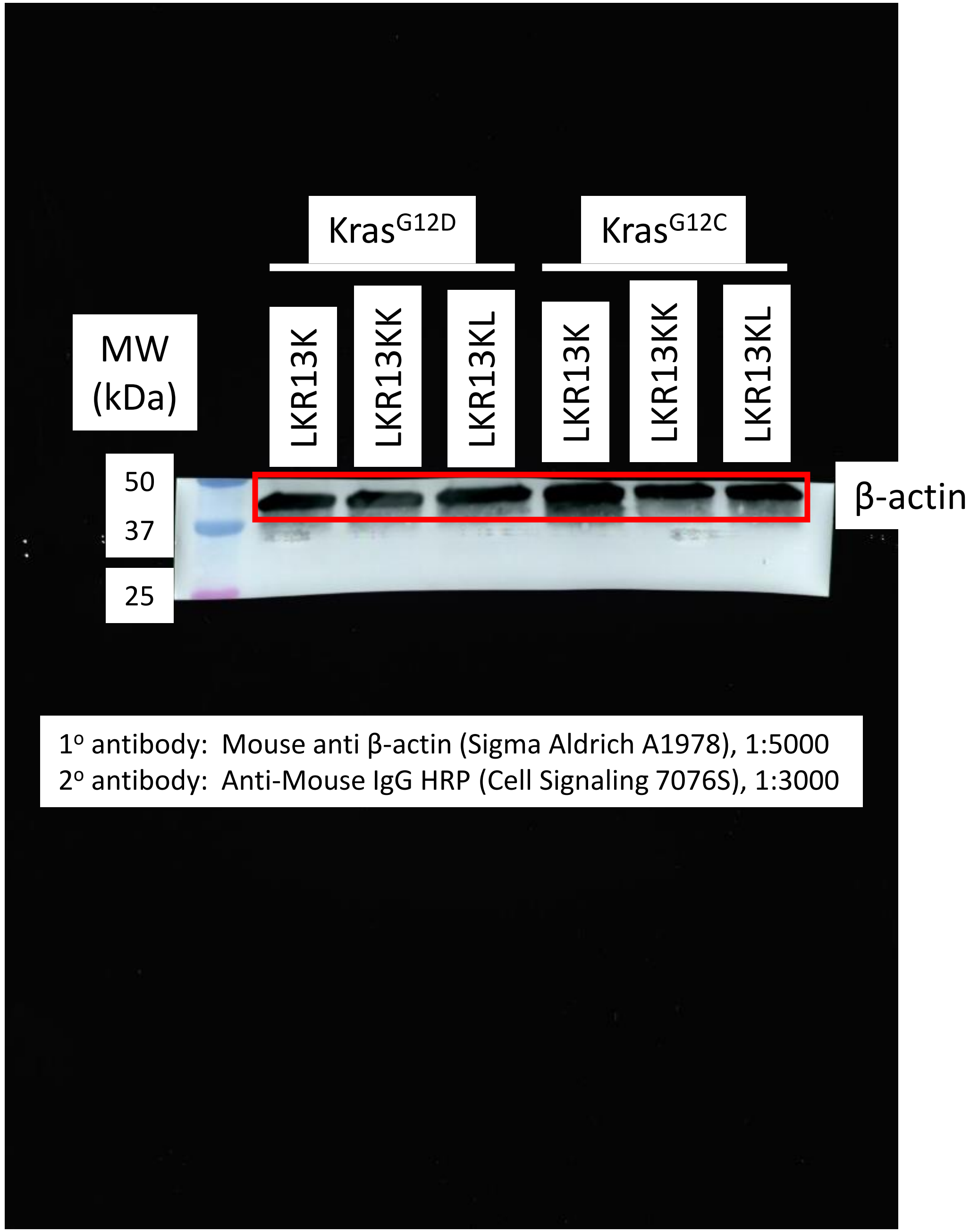
Kras^{G12D}
Kras^{G12C}
MW (kDa)
LKR13K
LKR13KK
LKR13KL
LKR13K
LKR13KK
LKR13KL
50
37
25
β-actin
1° antibody: Mouse anti β-actin (Sigma Aldrich A1978), 1:5000
2° antibody: Anti-Mouse IgG HRP (Cell Signaling 7076S), 1:3000

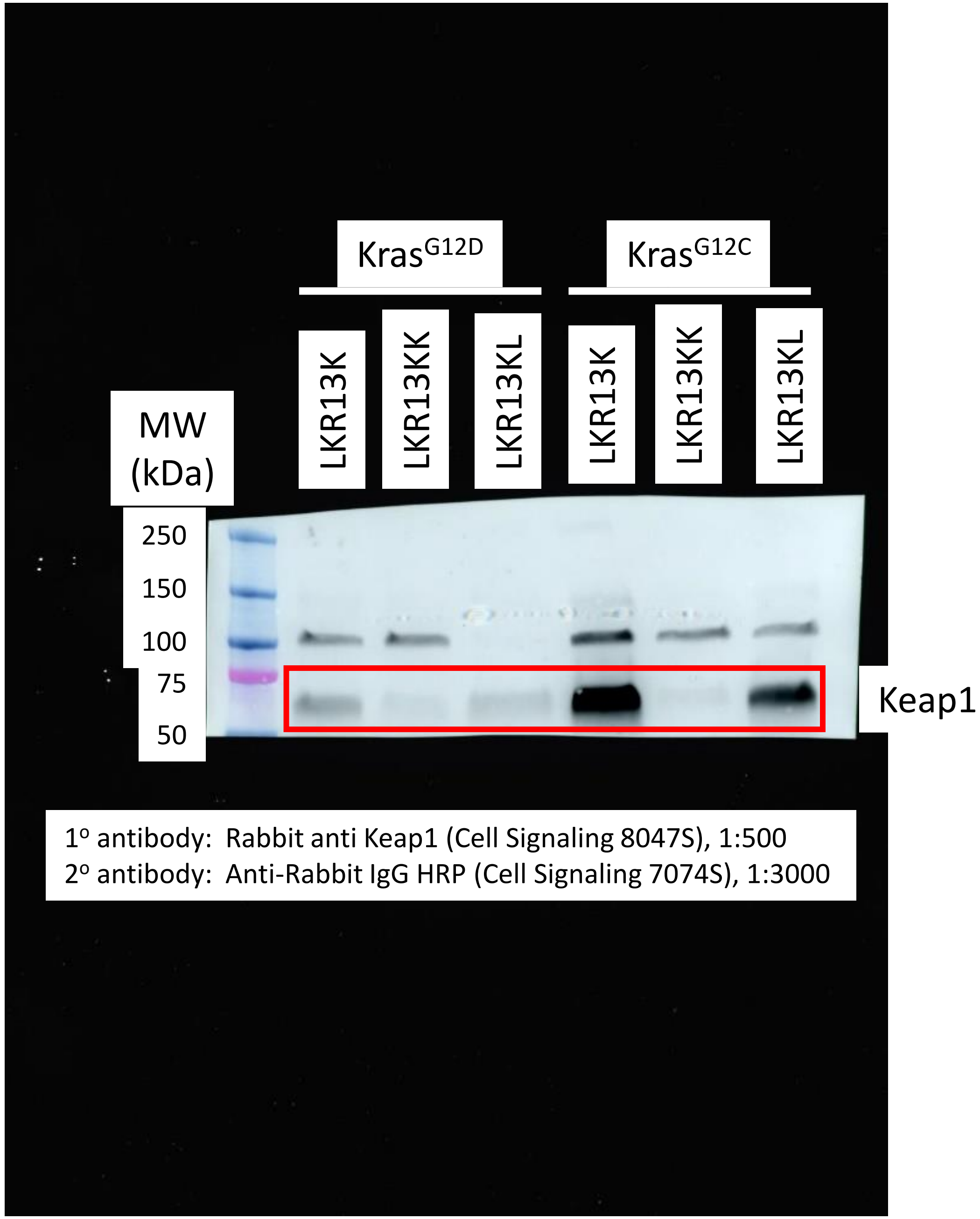
Kras^{G12D}
Kras^{G12C}
MW (kDa)
LKR13K
LKR13KK
LKR13KL
LKR13K
LKR13KK
LKR13KL
250
150
100
75
50
Keap1
1° antibody: Rabbit anti Keap1 (Cell Signaling 8047S), 1:500
2° antibody: Anti-Rabbit IgG HRP (Cell Signaling 7074S), 1:3000

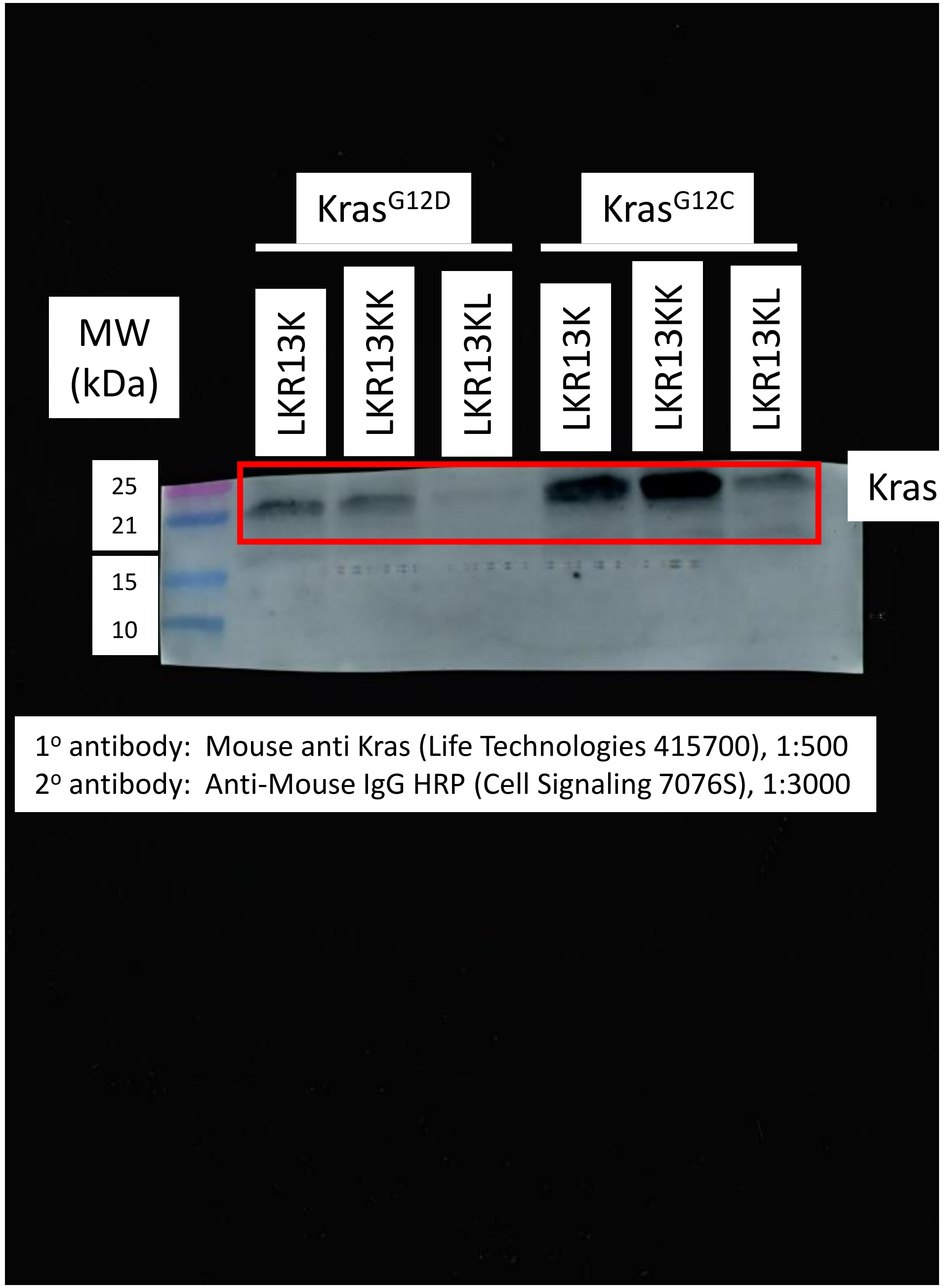
Kras^{G12D}
Kras^{G12C}
MW (kDa)
LKR13K
LKR13KK
LKR13KL
LKR13K
LKR13KK
LKR13KL
25
21
15
10
Kras
1° antibody: Mouse anti Kras (Life Technologies 415700), 1:500
2° antibody: Anti-Mouse IgG HRP (Cell Signaling 7076S), 1:3000

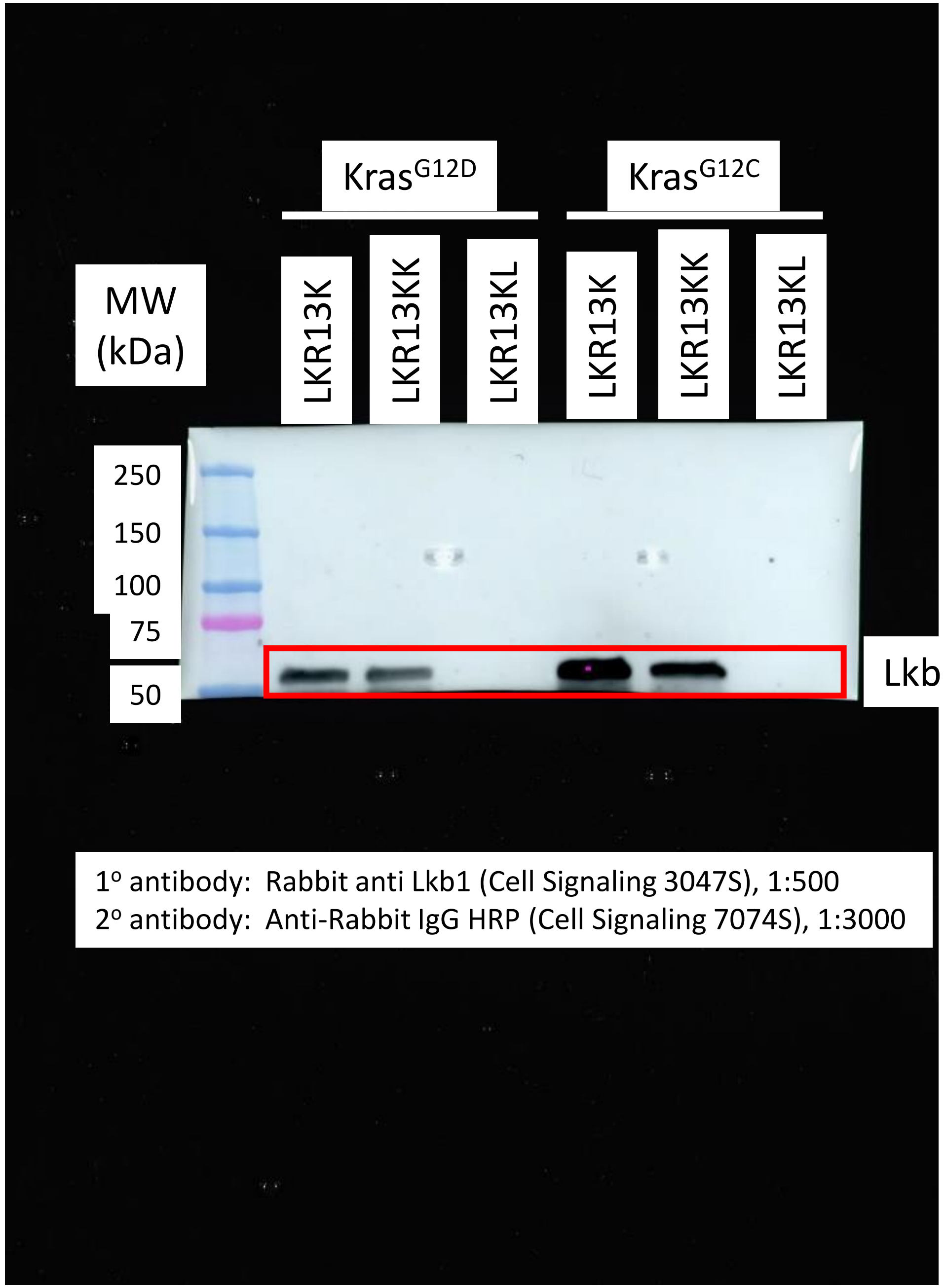
Kras^{G12D}
Kras^{G12C}
MW (kDa)
LKR13K
LKR13KK
LKR13KL
LKR13K
LKR13KK
LKR13KL
250
150
100
75
50
Lkb1
1° antibody: Rabbit anti Lkb1 (Cell Signaling 3047S), 1:500
2° antibody: Anti-Rabbit IgG HRP (Cell Signaling 7074S), 1:3000

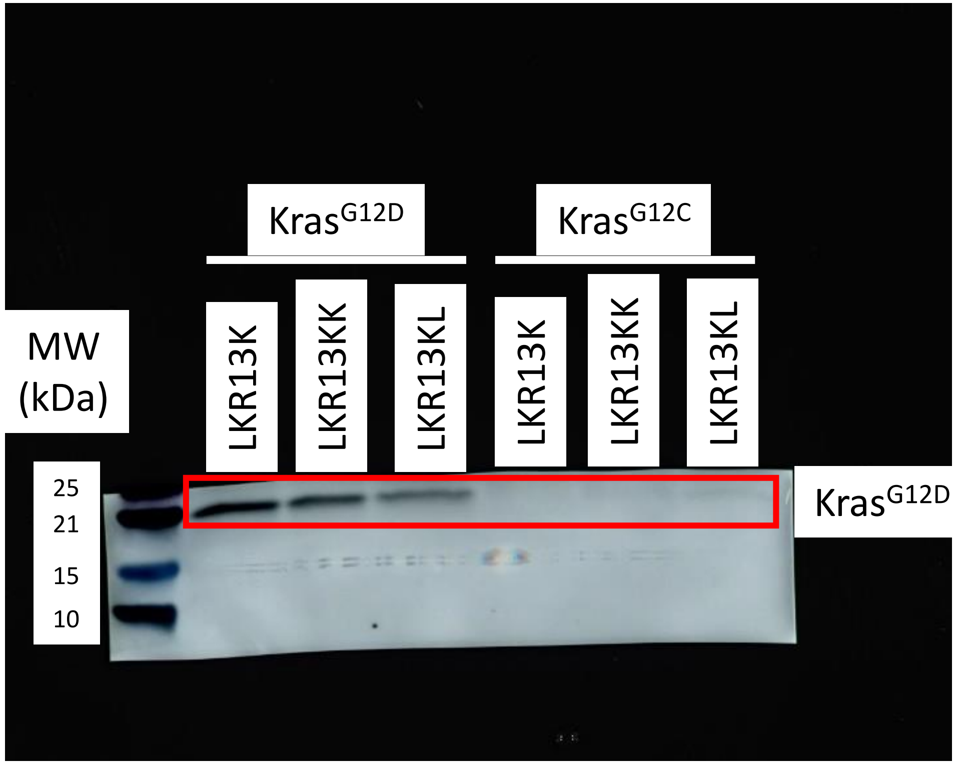

1° antibody: Rabbit anti Kras^{G12D} (Life Technologies MA536256), 1:500
2° antibody: Anti-Rabbit IgG HRP (Cell Signaling 7074S), 1:3000

Extended Data Figure 3

Technical Information

- If not otherwise mentioned 20 µg protein / lane loaded
- Same antibody dilutions used for 6 and 24h samples
- Exposure times to detect ECL signals are listed, and same exposures were used for 6 and 24h samples
- To safe extract, antibodies etc. we typically cut membranes to only blot the relevant region of the membrane with the respective antibodies, that allows typically 2-4 antibodies to use per membrane
- X-ray scans were either in total or often only the relevant region scanned
- Note the colour code which indicates correlating membranes
- Sometimes several exposure or several blots are shown, the once taken for the figures are indicated in bold and have a star (*)

SW837

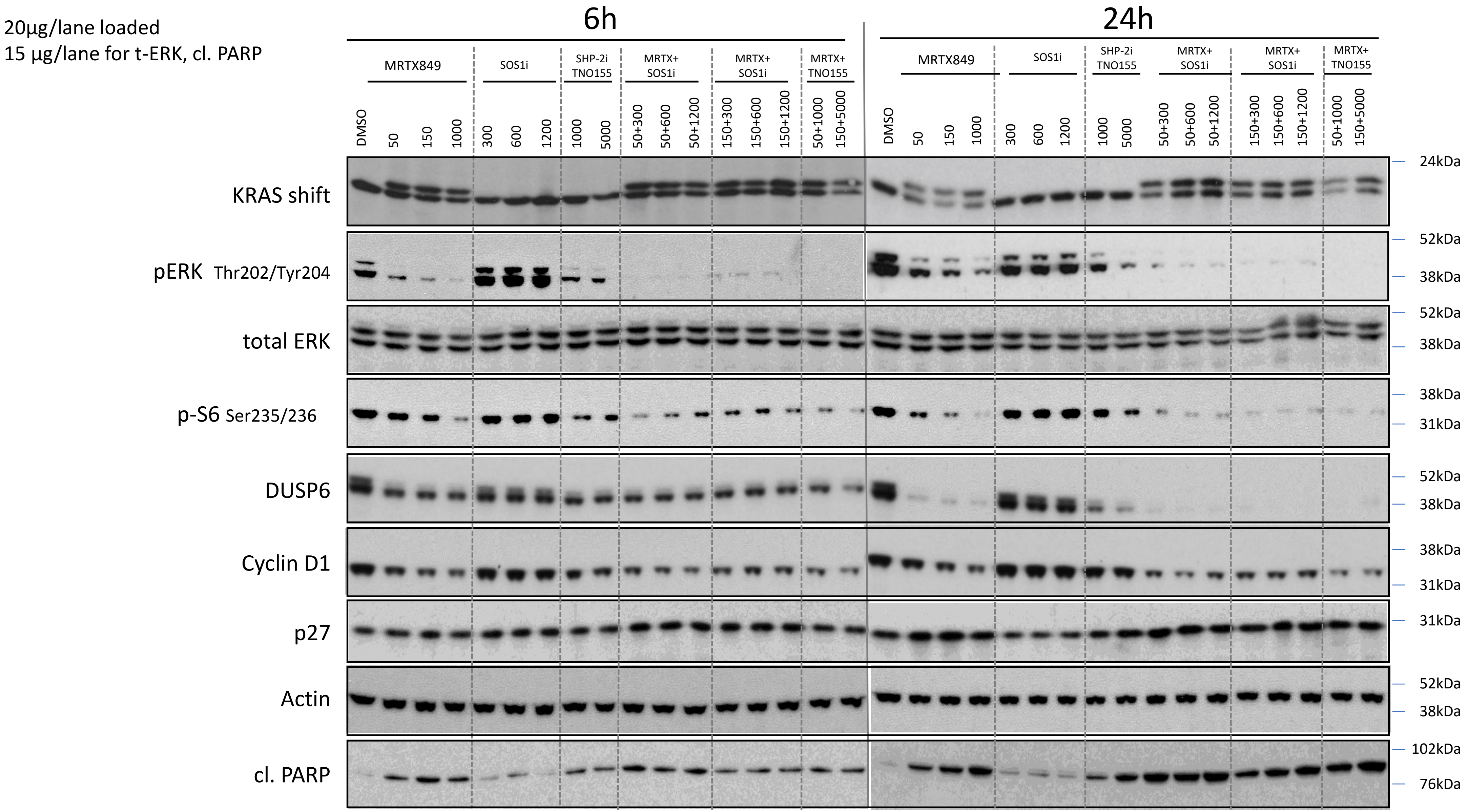

20µg/lane loaded
15 µg/lane for t-ERK, cl. PARP
6h
24h
MRTX849
SOS1i
SHP-2i TNO155
MRTX+ SOS1i
MRTX+ TNO155
DMSO
50
150
1000
300
600
1200
5000
50+300
50+600
50+1200
150+300
150+600
150+1200
50+1000
150+5000
KRAS shift
pERK Thr202/Tyr204
total ERK
p-S6 Ser235/236
DUSP6
Cyclin D1
p27
Actin
cl. PARP
24kDa
52kDa
38kDa
31kDa
102kDa
76kDa

SW837

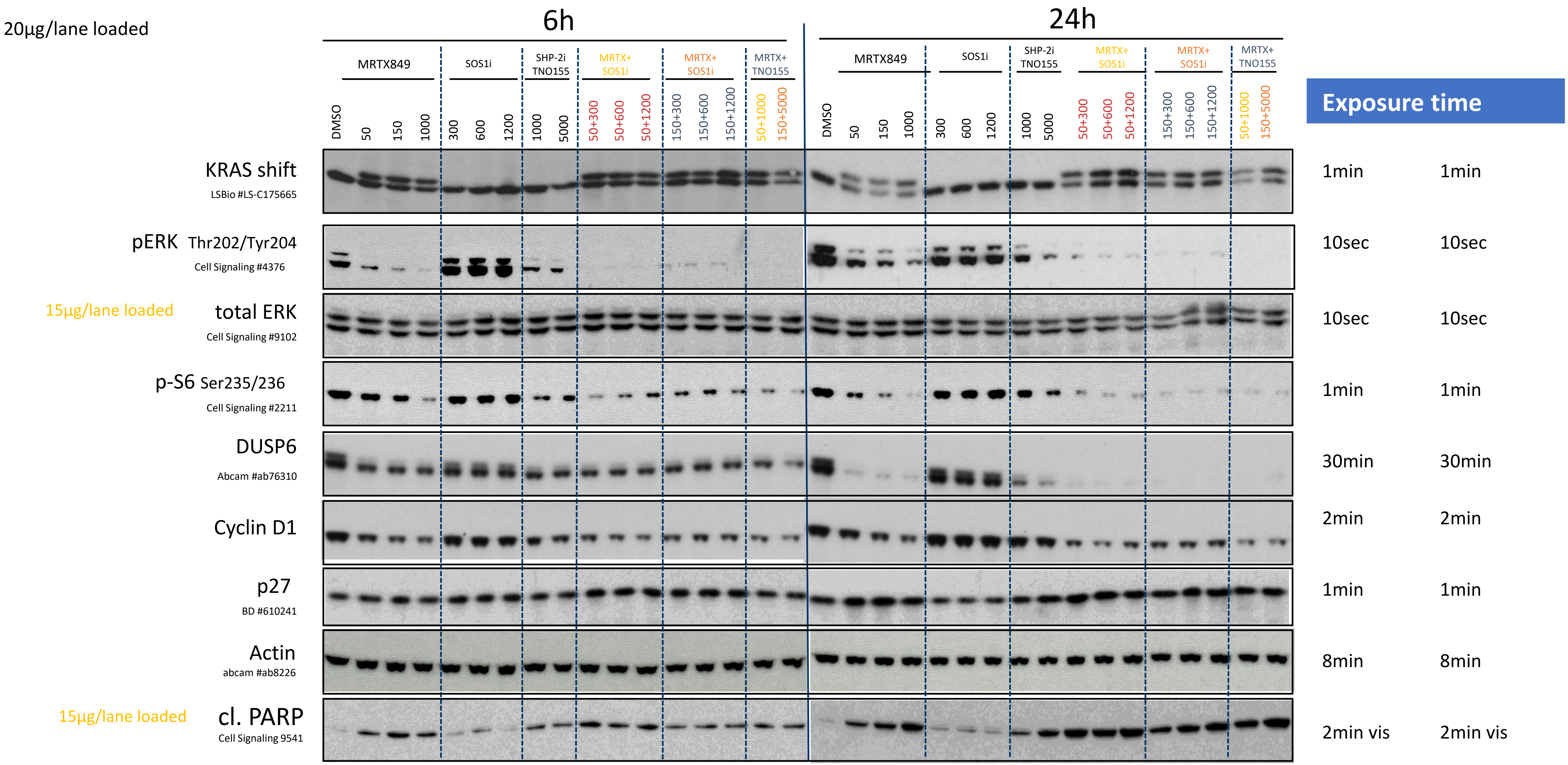

20µg/lane loaded
6h
24h
MRTX849
SOS1i
SHP-2i TNO155
MRTX+ SOS1i
MRTX+ SOS1i
MRTX+ TNO155
DMSO
50
150
1000
300
600
1200
1000
5000
50+300
50+600
50+1200
150+300
150+600
150+1200
50+1000
150+5000
KRAS shift
LSBio #LS-C175665
pERK Thr202/Tyr204
Cell Signaling #4376
15µg/lane loaded
total ERK
Cell Signaling #9102
p-S6 Ser235/236
Cell Signaling #2211
DUSP6
Abcam #ab76310
Cyclin D1
p27
BD #610241
Actin
abcam #ab8226
15µg/lane loaded
cl. PARP
Cell Signaling 9541
Exposure time
1min 1min
10sec 10sec
10sec 10sec
1min 1min
30min 30min
2min 2min
1min 1min
8min 8min
2min vis 2min vis

Scanned blots of 6h treatment:

Actin 42kDa

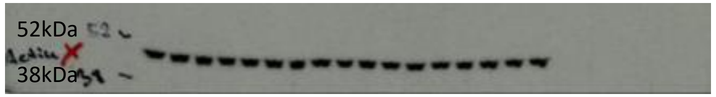

8min exposure *

p27 27kDa

31kDa
24kDa

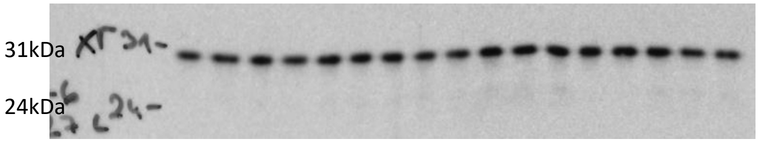

1min exposure *

pS6 32kDa

38kDa
31kDa

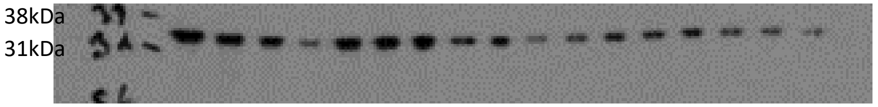

10sec exposure *

31kDa

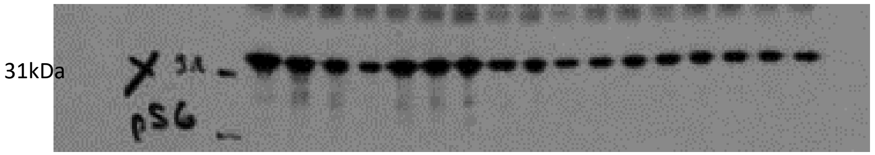

31kDa

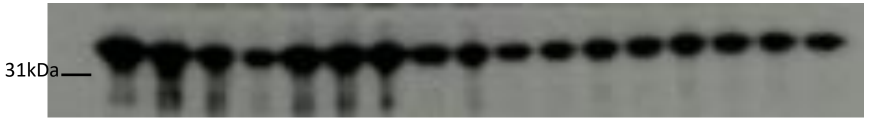

5min exposure

Cyclin D1 36kDa

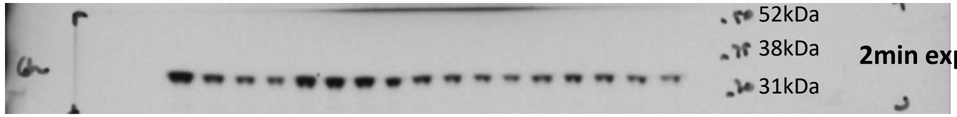

2min exposure *

Scanned blots of 6h treatment:

KRAS 21,7kDa

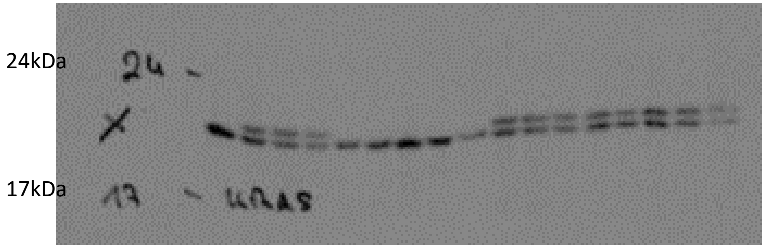

10sec exposure

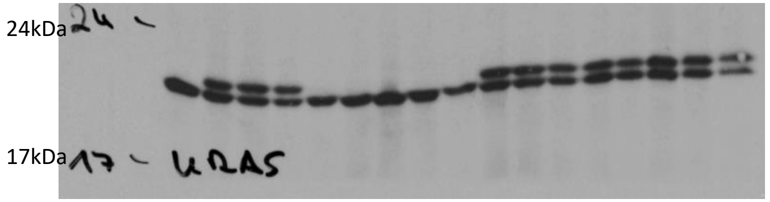

1min exposure *

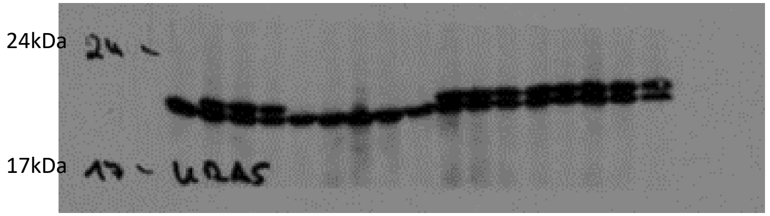

5min exposure

ERK 42/44kDa

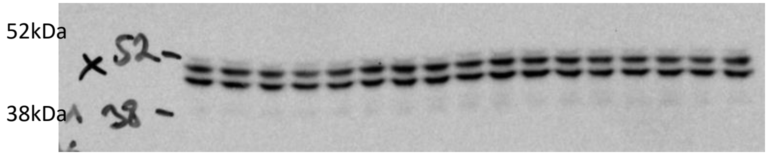

10sec exposure *

pERK 42/44kDa

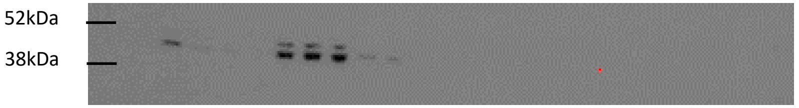

3sec exposure

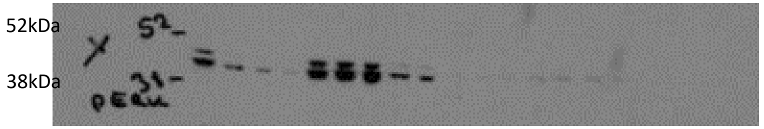

10sec exposure *

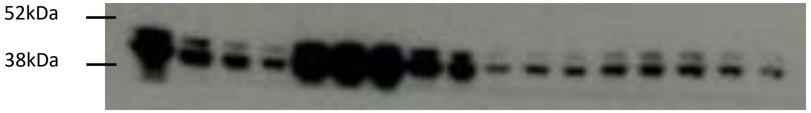

DUSP6 42/44kDa

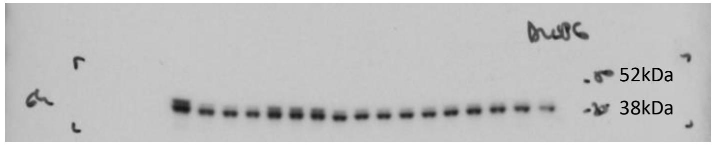

30min exposure *

Cl-PARP 89 kDa

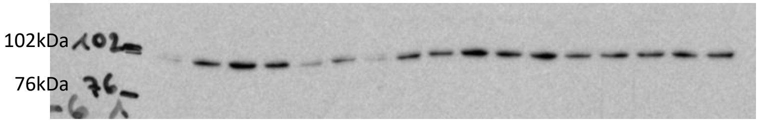

2min exposure *

Visualizer

Scanned blots of 24h treatment:

ERK 42/44kDa

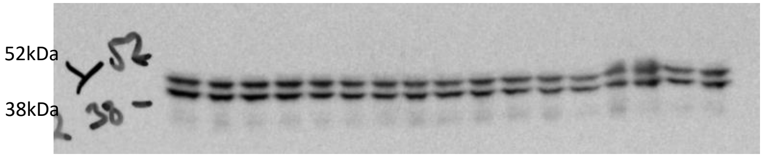

10sec exposure *

p27 27kDa

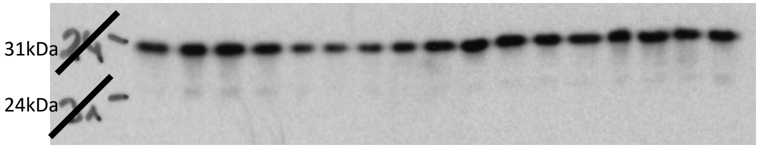

reverse and eroneous hand-written label; for reference of p27 protein migration towards position of markers please see slides 21 and 32.

1min exposure *

Cl-PARP 89 kDa

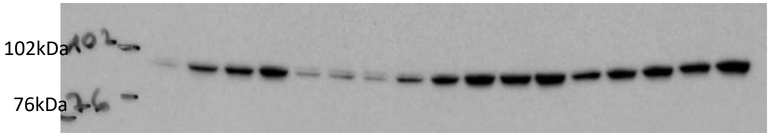

2min exposure visualizer *

pS6 32kDa

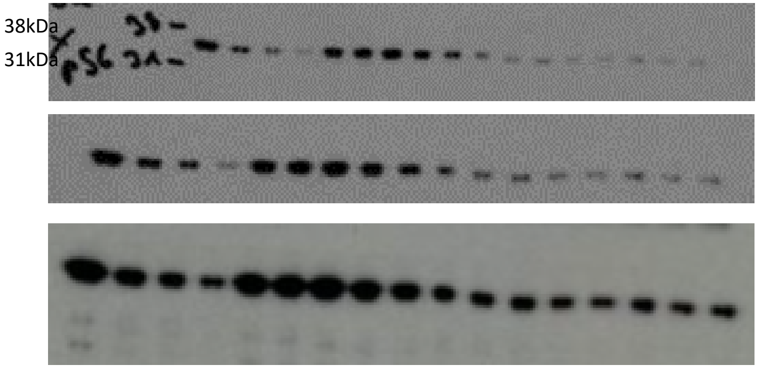

1 min exposure *

5 min exposure

8 min exposure

Cyclin D1 36kDa

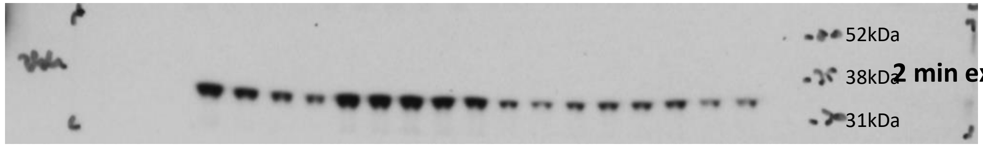

2 min exposure *

Scanned blots of 24h treatment:

DUSP6 42/44kDa

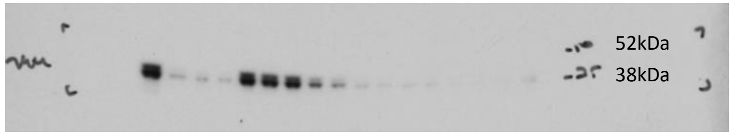

30min exposure *

Actin 42kDa

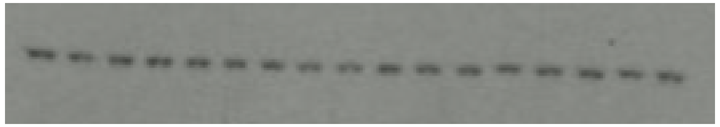

3 min exposure

52kDa

38kDa

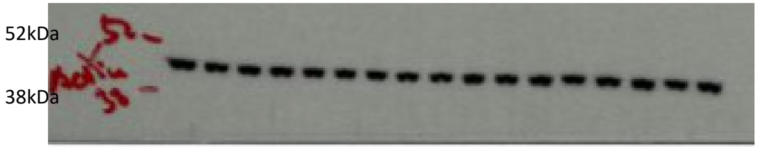

8 min exposure *

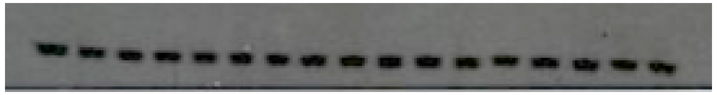

10 min exposure

KRAS 21,7kDa

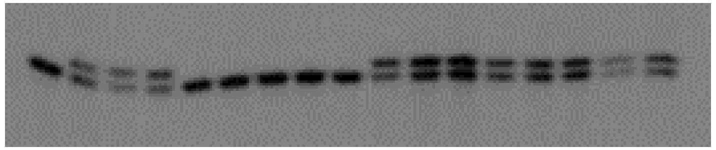

1 min exposure *

24kDa

17kDa

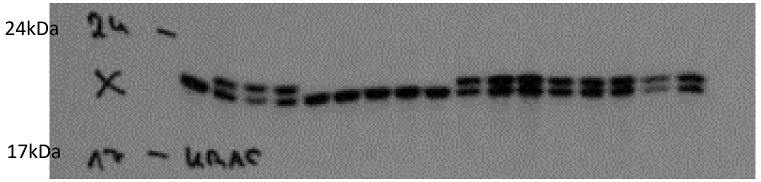

5 min exposure

pERK 42/44kDa

52kDa

38kDa

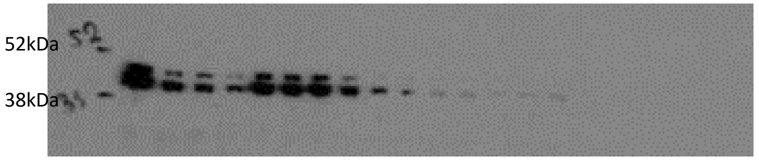

10sec exposure *

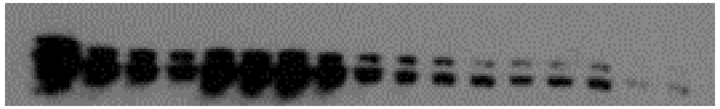

5 min exposure

Extended Data 7f

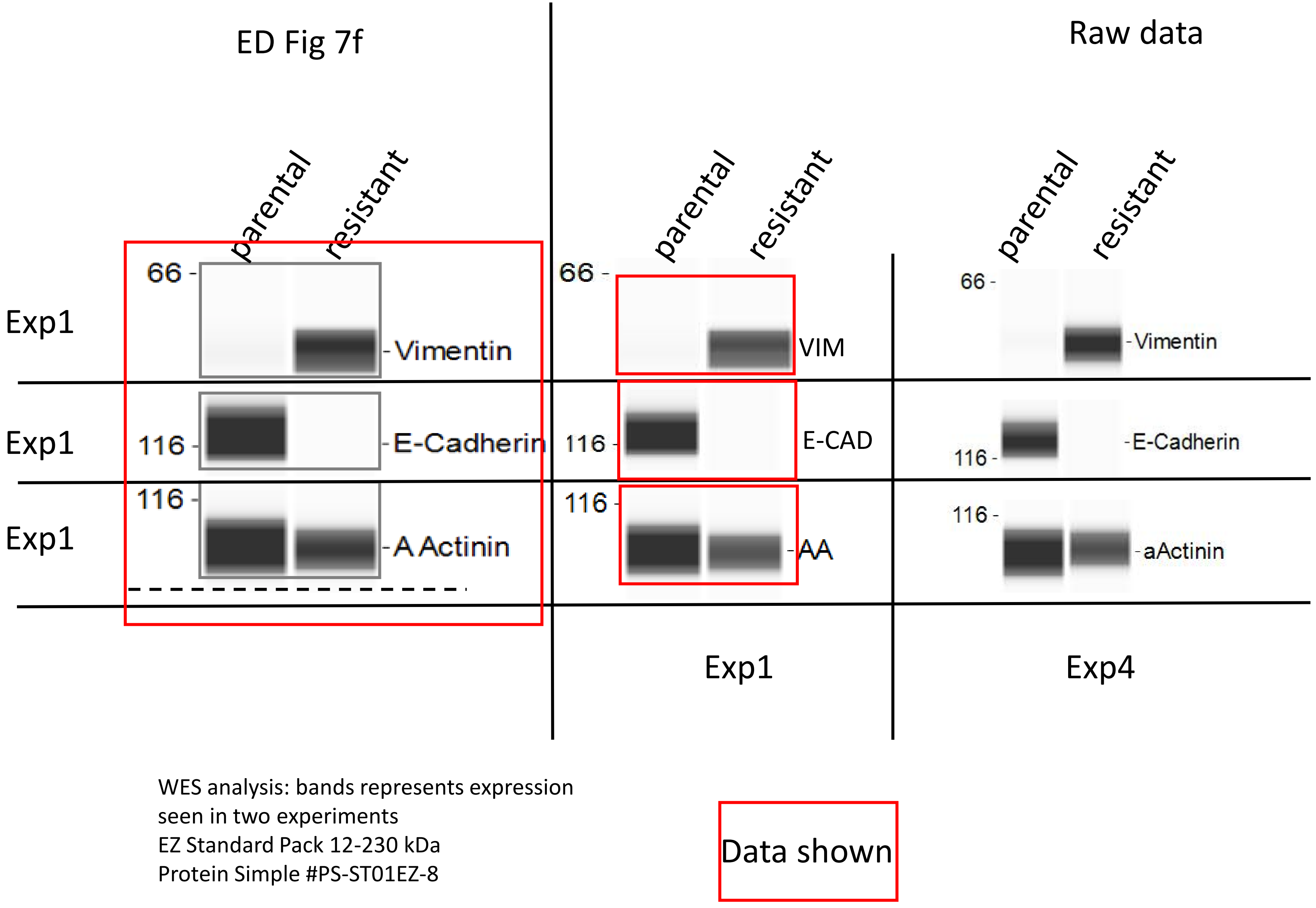

WES analysis: bands represents expression
seen in two experiments
EZ Standard Pack 12-230 kDa
Protein Simple #PS-ST01EZ-8

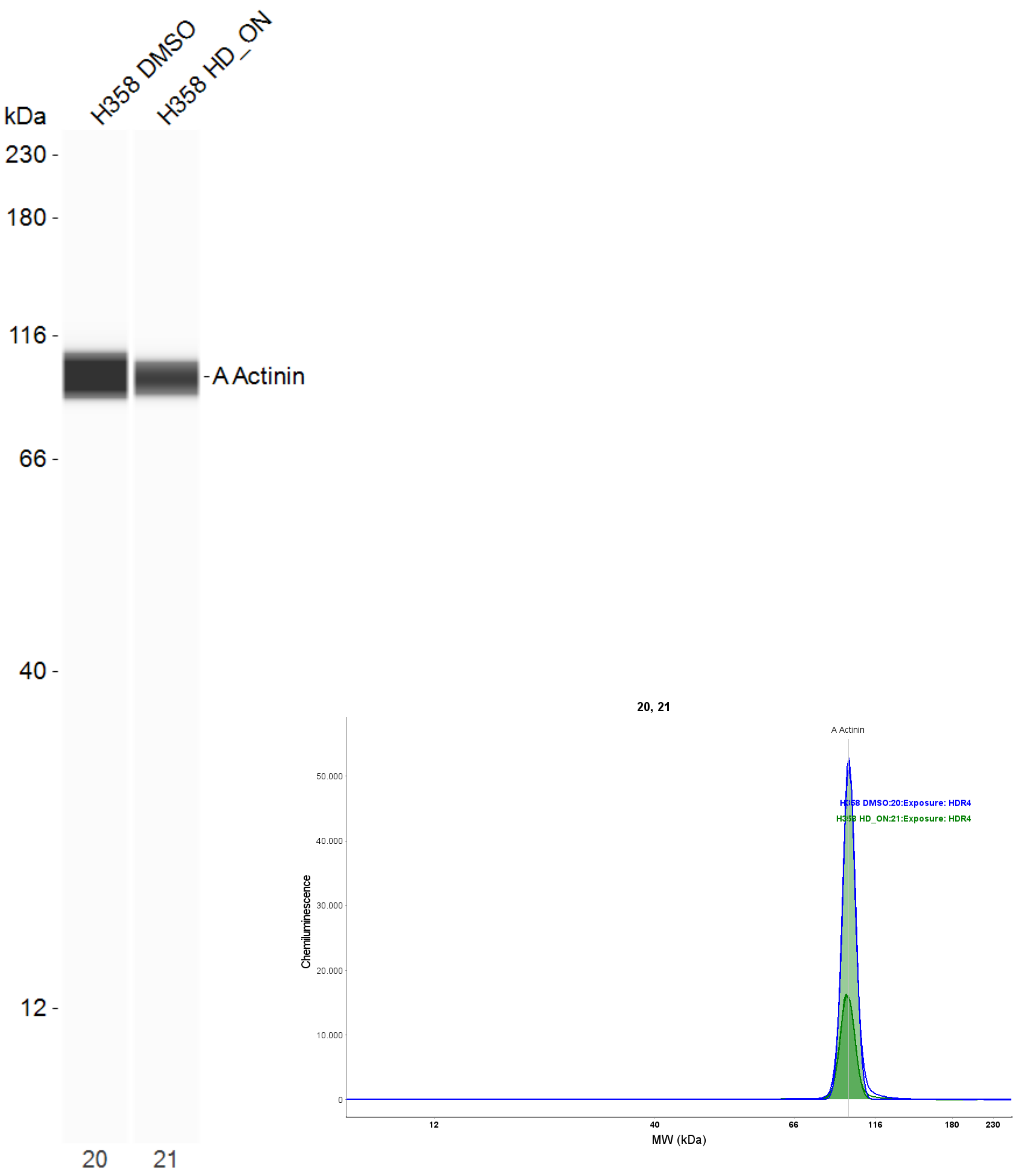
H358 DMSO
H358 HD_ON
kDa
230
180
116
66
40
12
A Actinin
20
21
20, 21
A Actinin
H358 DMSO:20:Exposure: HDR4
H358 HD_ON:21:Exposure: HDR4
Chemiluminescence
MW (kDa)

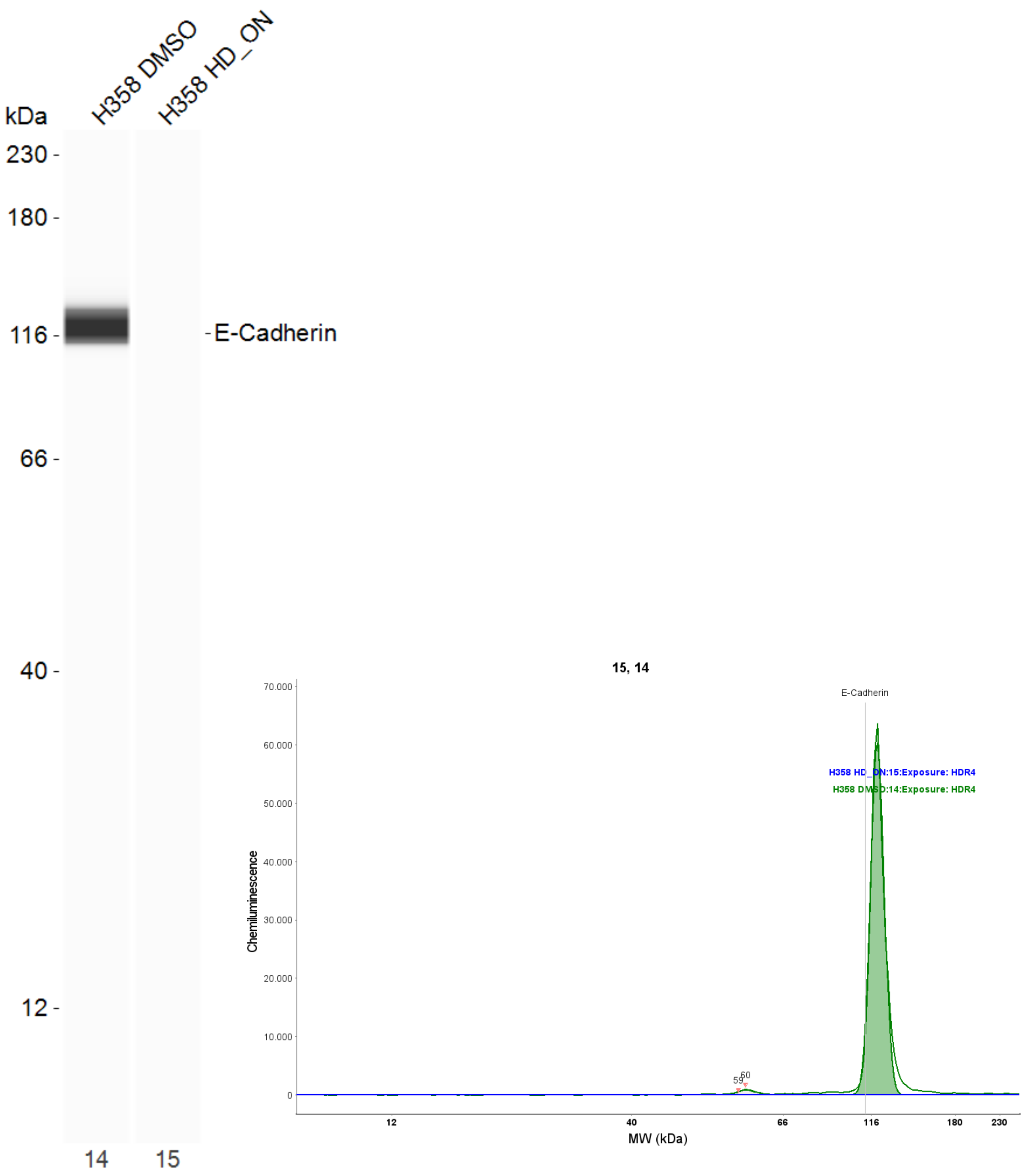
H358 DMSO
H358 HD_ON
kDa
230
180
116
66
40
12
E-Cadherin
14
15
15, 14
E-Cadherin
H358 HD_ON:15:Exposure: HDR4
H358 DMSO:14:Exposure: HDR4
Chemiluminescence
MW (kDa)

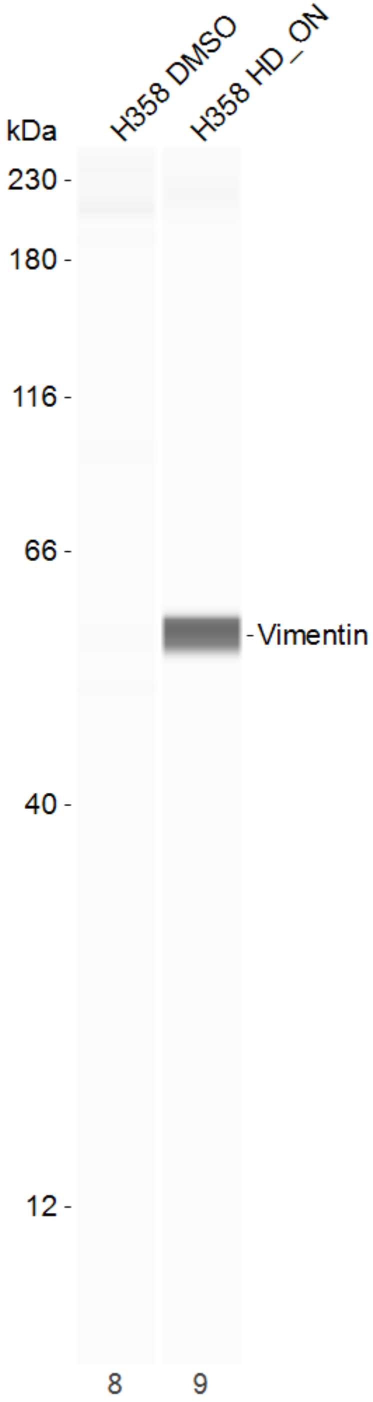
H358 DMSO
H358 HD_ON
kDa
230
180
116
66
40
12
Vimentin
8
9

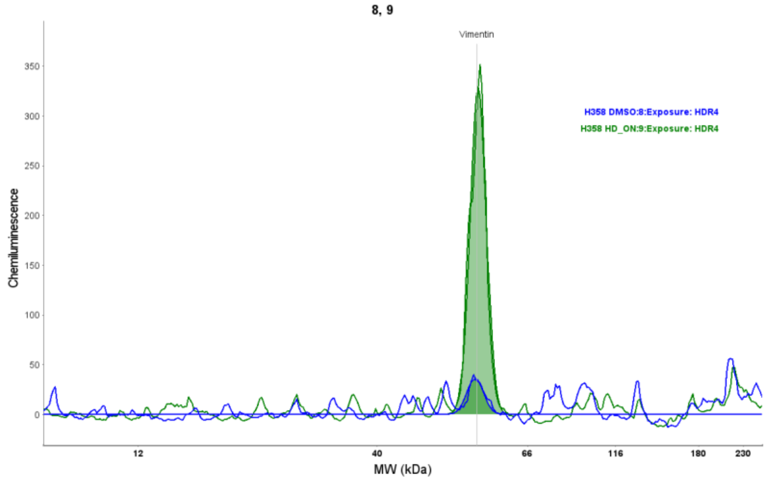
8, 9
Vimentin
H358 DMSO:8:Exposure: HDR4
H358 HD_ON:9:Exposure: HDR4
Chemiluminescence
MW (kDa)
